# Nucleotide polymorphism assay for the identification of west African group *Bacillus anthracis:* a lineage lacking anthrose

**DOI:** 10.1186/s12866-019-1693-2

**Published:** 2020-01-07

**Authors:** Diansy Zincke, Michael H. Norris, Berzhan Kurmanov, Ted L. Hadfield, Jason K. Blackburn

**Affiliations:** 1 Spatial Epidemiology & Ecology Research Laboratory, Department of Geography, University of Florida, Gainesville, FL USA; 2 Emerging Pathogens Institute, University of Florida, Gainesville, FL USA

**Keywords:** *Bacillus anthracis*, SNP, Anthrose, Anthrax, West Africa, Genotyping

## Abstract

**Background:**

The exosporium of the anthrax-causing *Bacillus anthracis* endospores display a tetrasaccharide composed of three rhamnose residues and an unusual sugar termed anthrose. Anthrose is a proposed potential target for immunotherapy and for specific detection of *B. anthracis*. Although originally thought to be ubiquitous in *B. anthracis*, previous work identified an anthrose negative strain from a West African lineage isolated from cattle that could represent a vaccine escape mutant. These strains carry genes required for expression of the anthrose operon but premature stop codons resulting from an 8-bp insertion in BAS3320 (an amino-transferase) and a C/T substitution at position 892 of the BAS3321 (a glycosyltransferase) gene prevent anthrose expression. Various other single nucleotide polymorphisms (SNPs) have been identified throughout the operon and could be the basis for detection of anthrose-deficient strains.

**Results:**

In this study, we evaluated rhAmp genotypic assays based on SNPs at positions 892 and 1352 of BAS3321 for detection and differentiation of anthrose negative (Ant^−^) West African strains. Discrimination of anthrose negative West African isolates was achieved with as low as 100 fg of DNA, whereas consistent genotyping of Sterne necessitated at least 1 pg of DNA.

**Conclusions:**

Screening of a global panel of *B. anthracis* isolates showed anthrose-expressing alleles are prevalent worldwide whereas the anthrose-deficient phenotype is to date limited to West Africa. Our work also revealed a third, previously unreported anthrose genotype in which the operon is altogether missing from a Polish *B. anthracis* isolate.

## Background

Anthrax, caused by the Gram-positive spore former *Bacillus anthracis*, is a worldwide zoonotic disease primarily affecting herbivores and livestock [1]. The most common route of exposure in grazing animals is ingestion of spores found in soil, grass and root materials. Humans are most often infected through contact with contaminated animal products (cutaneous anthrax) or through ingestion of contaminated meat (gastrointestinal anthrax). Human disease is most successfully controlled through preventative livestock vaccination [2, 3].

The outermost layer of the *B. anthracis* exosporium consists of hair-like filaments formed by trimers of BclA, a highly immunogenic collagen-like glycoprotein, termed the exosporium nap [4–6]. Previous work identified two oligosaccharides, a 324-Da disaccharide and a 715-Da tetrasaccharide associated with BclA [7]. Specifically, the central region of BclA contains collagen-like repeats binding multiple copies of the tetrasaccharide through N-acetylgalactosamine (GalNAc) moieties. The tetrasaccharide is composed of three rhamnose residues and a nonreducing terminal sugar (2-O-methyl-4-(3-hydroxy-3-methylbutamido)-4,6-dideoxy-D-glucose) that was previously undescribed [7]. Based on initial limited evidence, this novel sugar termed anthrose, was identified as a unique feature of the *B. anthracis* endospore and thus a potential target for immunotherapy and diagnosis [7–10].

Subsequent work examined the immunogenic character of the tetrasaccharide and the anthrose moiety. Specifically, Tamborrini and colleagues used a synthetic tetrasaccharide conjugated to the keyhole-limpet-hemocyanine (KLH) carrier protein to elicit IgG antibodies reacting with the tetrasaccharide after immunization in mice [8, 9]. The tetrasaccharide specific IgG antibodies were also shown to bind *B. anthracis* spores [9]. Similarly, Mehta et al. [11] reported serum of rabbits immunized with Sterne spores reacted with a synthesized anthrose-containing trisaccharide conjugated to protein carrier KLH, but not with the native KLH. The work further identified a moiety of anthrose, 4″-(3-methylbutyryl), essential for binding of the anthrose-containing trisaccharide to anti-spore antiserum. Others studies similarly point to the immunogenic nature of the anthrose-containing tetrasaccharide [10, 12, 13].

**Fig. 1:**
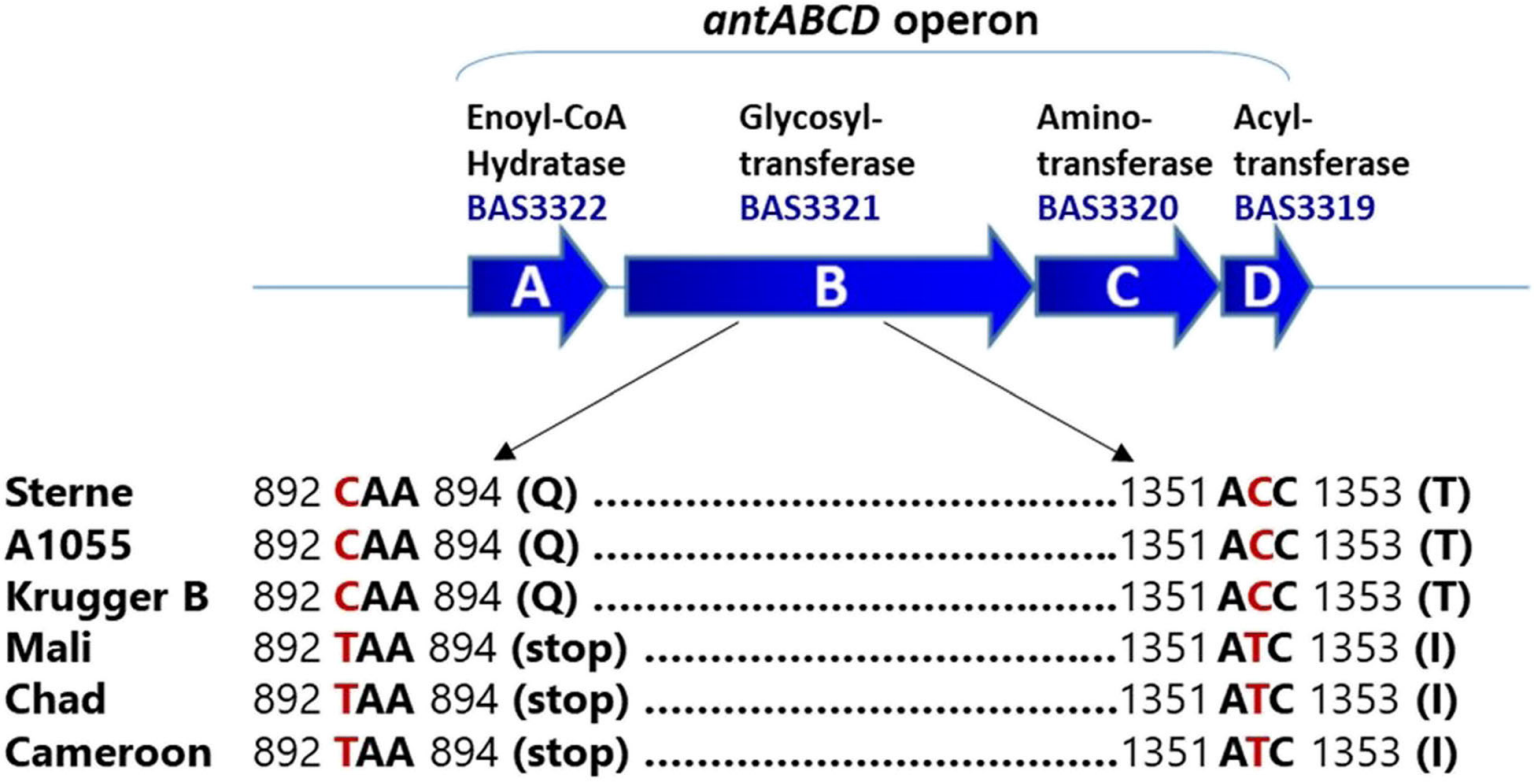
The previous works of Dong et al. [14] and Tamborrini et al. [16] showed anthrose is encoded by a four-gene operon and the West African lineages display a number of mutations, such as the two SNPS shown at positions 892 and 1352 of the BAS3321 gene (red), which prevent anthrose expression. These same mutations are present in Nigerian strains [17]

The anthrose biosynthetic operon was previously characterized and appears to be ubiquitous in *B. anthracis* [14, 15]. All sequenced *B. anthracis* genomes in NCBI (129/129) carry the complete *antABCD* operon with percent identities ranging from 97 to 100% when compared to Sterne (Additional file 1: Table S1). Recent work, however, identified isolates from Mali, Chad, and Cameroon failing to express anthrose [16]. These strains carry the *antABCD* operon but premature stop codons resulting from an 8-bp insertion in BAS3320 and a SNP in BAS3321 prevent anthrose expression (Fig. 1). A second non-synonymous substitution at position 1352 of BAS3321 was also identified in these western African strains (Fig. 1). The authors concluded these isolates were part of a novel and distinct *B. anthracis* lineage limited to western Africa [16].

The existence of a unique western African lineage was originally reported by two different groups [18, 19]. Specifically, Lista et al. subtyped strains from Cameroon into a novel lineage termed E, using a 25 loci multi-locus variable number tandem repeat system (MLVA-25) [18]. Prior to that, Maho and colleagues investigated the genetic diversity of Chadian strains isolated from cattle carcasses by MLVA-8 and direct-repeat markers [19]. These isolates formed a new genetic group within the A clade designated as Aβ. Similarly, work examining bovine strains from different areas of Cameroon placed them in the new Aβ cluster of the A branch along with the previously studied Chadian strains [20]. The authors further noted two previously examined Cameroon strains by Lista et al. had MLVA-8 profiles identical to the newly examined Cameroon strains. Recent work similarly MLVA-25 genotyped Nigerian strains isolated from cattle and additional Chadian strains and assigned them to the West African Group (WAG; synonymous with E/Aβ) [21]. A representative isolate from Nigeria was sequenced and found to carry the same anthrose deficient genotype described in isolates from Mali, Cameroon and Chad [16, 21].

The anthrose deficient genotype is thus far limited to WAG isolates, as this characteristic is not observed elsewhere. The SNPs identified in the *antABCD* operon of such strains represent a tool for discrimination of the WAG lineage. Here we evaluate two SNPs resulting in nonsynonymous substitutions in the WAG *B. anthracis*. Using a rhAmp genotyping assay (Integrated DNA Technologies, Iowa), SNPs at positions 892 and 1352 of the BAS3321 gene of the anthrose operon were interrogated (Fig. 1). The rhAmp technology uses blocked primers to prevent extension and minimize non-specific amplification. Extension is conditioned upon cleavage and removal of the blocking group by RNase H2, which itself requires binding of primer to its perfect complement.

Our work describes the development of two SNP assays for differentiation of the *B. anthracis* WAG lineage. Conservation of the *antABCD* operon, with special attention to the 892 and 1352 *antB* SNPs, was examined in all available *B. anthracis* genomes in GenBank. The sensitivity of each assay was established and a diverse panel of *B. anthracis* strains including representatives of several major lineages as well as actively circulating strains in livestock and wildlife, was screened. In addition, we tested *Bacillus cereus* biovar *anthracis* (Bcbva) isolates from Côte d’Ivoire. Similar to WAG, Bcbva circulates in western Africa and causes anthrax-like disease due to the presence of pXO1- and pXO2-like plasmids [22, 23]. Giving the strong conservation between the anthrose operons of both pathogens (99.1% identity), we examined whether the anthrose SNP assays could be used to differentiate Bcbva from other local non-anthrose expressing strains.

## Results

### Bioinformatics analyses

All sequenced *B. anthracis* genomes available in GenBank were screened for the presence of the anthrose operon. The operons were remarkably conserved sharing 97–100% homology with Sterne (Additional file 1: Table S1 and Additional file 2: Figure S1). The unique WAG SNPs were not identified in any of the currently sequenced *B. anthracis* strains in GenBank. Interestingly, a strain isolated from an injecting heroin user (str. Heroin Ba4599, accession no. AGQP01000002.1,

https://www.ncbi.nlm.nih.gov/nuccore/AGQP01000002.1/) had a G/T substitution at position 418 of the *antB* gene resulting in a premature stop codon unobserved in the other genomes [24].

### rhAmp genotyping assays

**Fig. 2:**
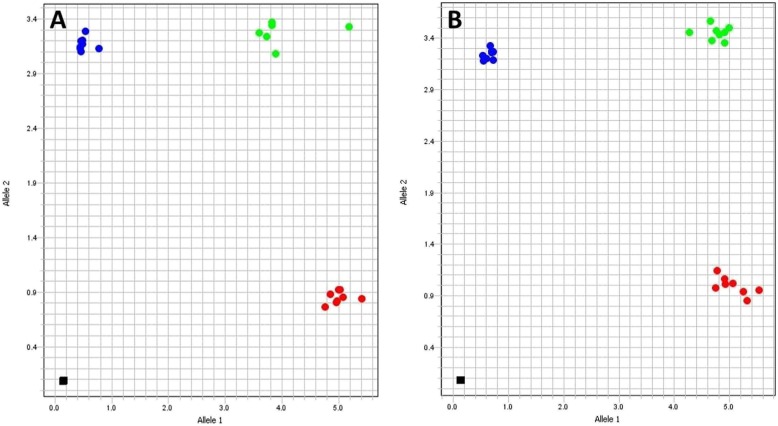
Evaluation of rhAmp genotyping assays. The 892 (Panel **a**) and 1352 (Panel **b**) genotyping assays were initially tested with 1 and 5 ng of DNA from both Sterne and a western African isolate in multiple replicates. The red dots along the *x* axis illustrate the Sterne-specific genotype with 1 and 5 ng samples clustering closely. Replicates of the Nigerian isolate clustered at the top left corner of the *y* axis in a concentration-independent manner (blue cluster). Heterozygous controls, consisting of equivalent amounts of Sterne and Nigerian DNA, were tested and are depicted in green at the top right corner of each plot. Black squares near the plot origin represent negative controls

The assays were initially tested with 1 and 5 ng of DNA from Sterne and from a previously sequenced WAG isolate. Primers specific for the anthrose-positive allele consistently yielded higher and stronger amplification with Sterne than with Nigerian DNA. On the other hand, the anthrose-deficient allele was preferentially amplified in the Nigerian background. Both DNA concentrations produced clear separation of genotypes (Fig. 2a, b). Cycle threshold (*C*_*T*_) values were slightly lower for the Nigerian strain across both assays (Additional file 3: Table S2).

### Level of detection of rhAmp genotyping assays

**Fig. 3:**
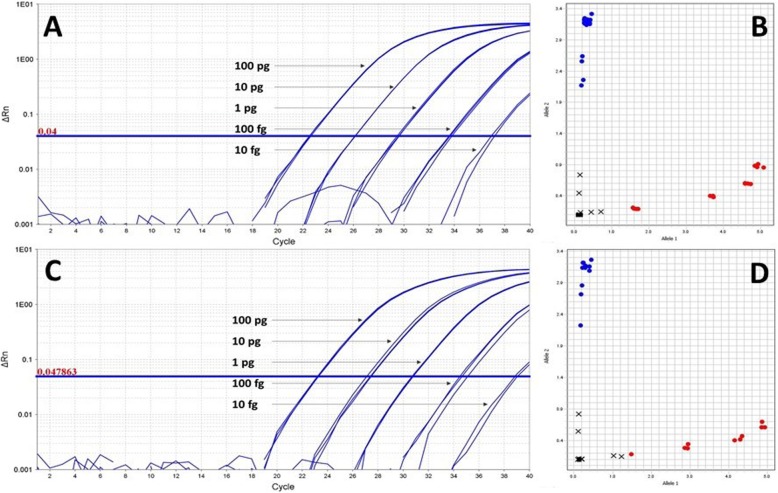
Real-time amplification plots of the 892 (**a**) and 1352 (**c**) rhAmp genotyping assays showing dilution curves of *B. anthracis* Sterne DNA. Ten-fold serial dilutions of Sterne DNA ranging from 10 fg to 100 pg were used to establish the limit of detection of each assay in triplicate (only fluorescence from Sterne specific allele primer is shown). Both assays consistently detected Sterne DNA at levels of 100 fg or higher but failed to produce consistent amplification with 10-fg samples. Similarly, a non-Sterne strain (Nigeria) exhibited a 100-fg limit of detection, with 10-fg samples producing sporadic amplification (data not shown). The corresponding endpoint allelic discrimination plots for the 892 (**b**) and 1352 (**d**) assays depict Sterne as red clusters and non-Sterne Nigeria DNA in blue. In both assays, non-Sterne DNA (blue clusters) is distinct from Sterne (red clusters). The genotypic separation of Nigeria DNA is achieved with as low as 100 fg, with 10 fg-samples being called undetermined (black X). Discrimination of Sterne is achieved with 1 pg of DNA in both assays (Panels **b** and **d**). Black squares near the plot origin represent negative controls. Undetermined calls are depicted as black Xs

rhAmp genotyping assays consistently detected *B. anthracis* and Nigerian samples with as low as 100 fg or ~ 17 genome equivalents (GE) of DNA, but only sporadically detected at the 10-fg level (Fig. 3a, c). Non-Sterne DNA was reliably and correctly genotyped with as little as 100 fg of DNA in both assays, whereas consistent discrimination of Sterne required 1 pg of DNA (Fig. 3b, d), with sporadic genotyping occurring at the 100-fg level. Ten-fg samples from both Sterne and non-Sterne strains, displaying only infrequent amplification, could not be discriminated by the QuantStudio 7 software. *C*_*T*_ values are shown in Additional file 3: Table S3.

Plasmid controls carrying the *antABCD* operons of Sterne or a WAG strain similarly failed to amplify and discriminate with 1.77 GE of plasmid (equivalent to 10 fg of *B. anthracis* chromosomal DNA) (Additional file 4: Figure S2). The clear separation of genotypes observed with 17.7 GE of plasmid DNA (equal to 100 fg of *B. anthracis* chromosomal DNA) shows that plasmid controls carrying the Sterne or WAG anthrose operons can be successfully used to differentiate between WAG and Sterne anthrose genotypes (Additional file 4: Figure S2B and S2D).

### Evaluation of diversity panel by rhAmp genotyping assays

A group of 49 different *B. anthracis* strains, including DNA extracted from Sterne 34F2 spores (Colorado Serum Company), was used to evaluate the two SNP assays. The panel was comprised of isolates from both laboratory and environmental sources and included representatives from the A, B and C lineages (Table 1). The 892 assay exhibited *C*_*T*_ values ranging from 18.8 to 22.7 with a mean of 20.4 and SD of 1.15 for the Sterne-like anthrose allele; for the WAG-like allele the mean *C*_*T*_ was 19.5, the SD was 0.33 and the range was from 19.0 to 19.9. For the 1352 assay, the samples displaying Sterne-like anthrose alleles had a mean *C*_*T*_ value of 21.1 with and SD was 1.1 and a range from 18.8 to 24.2; the WAG-like samples exhibited a mean *C*_*T*_ of 17.9 with an SD of 0.62 and a range from 18.8 to 24.2.

**Table 1:** Strains used in this study

A list/ original ID	UF ID	Description	Lineage	Strain details	Anthrose status^a^
A0077	UF00979		A.Br.001/002	Australia	Pos
A0987	UF00175		A.Br.005.006	Botswana	Pos
A0020	UF00552	Ames	A3b	CAMR/Porton	Pos
A0034	UF00503		A.Br.001/002	China	Pos
A0536	UF00950		A.Br.001/002	China	Pos
A0537	UF00965		A.Br.001/002	China	Pos
A0032	UF00502		A.Br.008/009	China	Pos
A0610	UF00791		A.Br.008/009	China	Pos
	Sterne	Sterne 34F2	GT59 (Lista et al. 2006)	Colorado Serum Co.	Pos
N/A	UF01137	WNA	A1.a	Colorado State Univ.	Pos
A0897	UF00727		A.Br.008/009	Ethiopia	Pos
A0389	UF00930		A.Br.001/002	Indonesia	Pos
A0084	UF00980	Vollum 1	A4	South Africa	Pos
A1040	UF00147	Western North America	A1.a	South Dakota	Pos
A2075	UF01105	Ames	A.Br.005.006	Tanzania	Pos
A0009	UF00553	Laboratory-Sterne	A3b		Pos
A0462	UF00738	Ames	A3b		Pos
A2017	UF01114	Sterne	A		Pos
A2076	UF01106	Ames	A3b		Pos
A2006	UF01096	Vollum	A4		Pos
A2073	UF01103	Vollum	A4		Pos
A3007	UF01043	WNA	A1.a		Pos
A2063	UF01062		West Africa Group (WAG)	Nigeria	Neg
A2064	UF01063		WAG	Nigeria	Neg
A2067	UF01075		WAG	Nigeria	Neg
	UF01052		WAG	Nigeria	Neg
A0402	UF00926		B.Br.CNEVA	France	Pos
A0333	UF00621		B.Br.CNEVA	Germany	Pos
A0451	UF00438		B.Br.001/002	Mozambique	Pos
A1085	UF00895		B.Br.CNEVA	Poland	Pos
A1088	UF00910		B.Br.CNEVA	Poland	Absent^b^
A0104	UF00839		B.Br.001/002	South Africa	Pos
A1055	UF00603		C.Br.A1005	USA	Pos
A0051	UF00343	Pasteur	A1.a (GT3, Lista et al. 2006)	CAMR/Porton	Pos
A0530	UF00878			Botswana	Pos
A1202	UF00049			Argentina	Pos
A1143	UF00393			Argentina	Pos
A1192	UF00055			Argentina	Pos
HHG80	UF01135			Etosha Natl Park (ENP), Namibia	Pos
14–1	UF02162			ENP, Nambia	Pos
68–1	UF02185			ENP, Nambia	Pos
A1073	UF00232			Chile	Pos
A0455	UF00408			Mozambique	Pos
A1052	UF00825			Belgian Congo	Pos
A0588	UF00933		A.Br.001/002	Gansu, China	Pos
A0542	UF00959		A.Br.001/002	Qingdao, China	Pos
A0549	UF00963		A.Br.001/002	Qingdao, China	Pos
A0538	UF00964		A.Br.001/002	Henan, China	Pos
A0599	UF00539		A.Br.001/002	Xinjiang, China	Pos
	Bc0001	Bcbva^c^		Côte d’Ivoire (Taï Natl Park)	Pos
	Bc0002	Bcbva		Côte d’Ivoire (Taï)	Pos
	Bc0007	Bcbva		Côte d’Ivoire (Taï)	Pos
	Bc0009	Bcbva		Côte d’Ivoire (Taï)	Pos
	Bc0011	Bcbva		Côte d’Ivoire (Taï)	Pos

^a^Positive indicates the strain carries the C SNP at positions 892 and 1352 of *antB* gene, whereas negative status corresponds to T substitutions at the same locations

^b^The anthrose operon could not be amplified in this strain

^c^*Bcbva Bacillus cereus* biovar *anthracis*

**Fig. 4:**
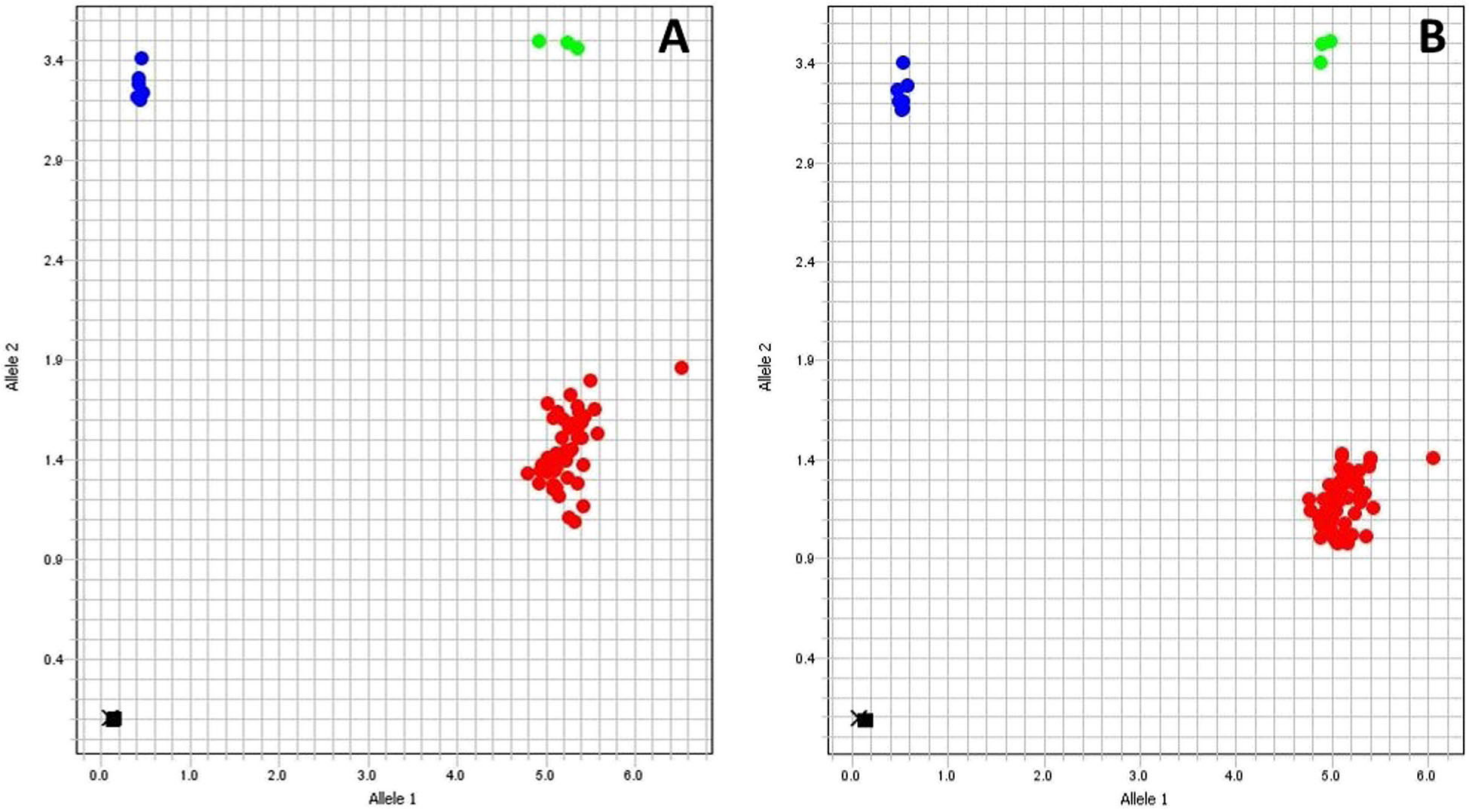
Evaluation of *B. anthracis* diversity panel. The discriminatory power of the 892 (**a**) and 1352 (**b**) genotyping assays was evaluated by testing a global panel of *B. anthracis* strains. The red dots along the *x* axis illustrate the Sterne-specific genotype (*n* = 44). Plasmid control replicates carrying the Sterne anthrose operon also clustered in this group (*n* = 3, red). The four Nigerian isolates (depicted in blue) clustered at the top left corner of the *y* axis along with plasmid control replicates carrying the anthrose operon from one of the western African strains (*n* = 3, blue). Heterozygous controls (green clusters) were tested by combining equivalent amounts of Sterne and Nigerian DNA and can help the software make appropriate determination when dealing with large panel of strains. Interestingly, UF00910 from Poland failed to amplify with either of the anthrose alleles after repeated attempts (depicted as X at plot origins). Black squares near the plot origin represent negative controls

The allelic discrimination plots show unequivocal separation of anthrose-producing (red cluster) and anthrose-non-producing strains (blue) (Fig. 4a, b). Although the panel included isolates from multiple regions of Africa, North and South America, Europe, Asia and Australia, only the strains from Nigeria were genotyped as anthrose-deficient strains.

Interestingly, one strain, UF00910 from Poland failed to produce amplification with primers specific for either the anthrose-positive or anthrose-negative alleles, shown as X at plot origins (Fig. 4a, b). UF00910 was strongly positive for both pXO1 and pXO2 plasmids and the Ba-1chromosomal marker [25]. The strain was further tested by conventional PCR with primers targeting the *antC* gene or the entire anthrose operon. Gradient PCRs failed to produce amplification of either *antC* or *antABCD* operon, while yielding appropriately sized products in the Sterne positive control (Additional file 5: Figure S3 and Additional file 6: Figure S4). Subsequent whole genome sequencing of this strain confirmed *B. anthracis*, the presence of pXO1 and pXO2, as well as a 59,157 bp deletion in the chromosome encompassing the anthrose operon and surrounding regions (to be published elsewhere).

### Evaluation of Bcbva strains by rhAmp genotyping assays

**Fig. 5:**
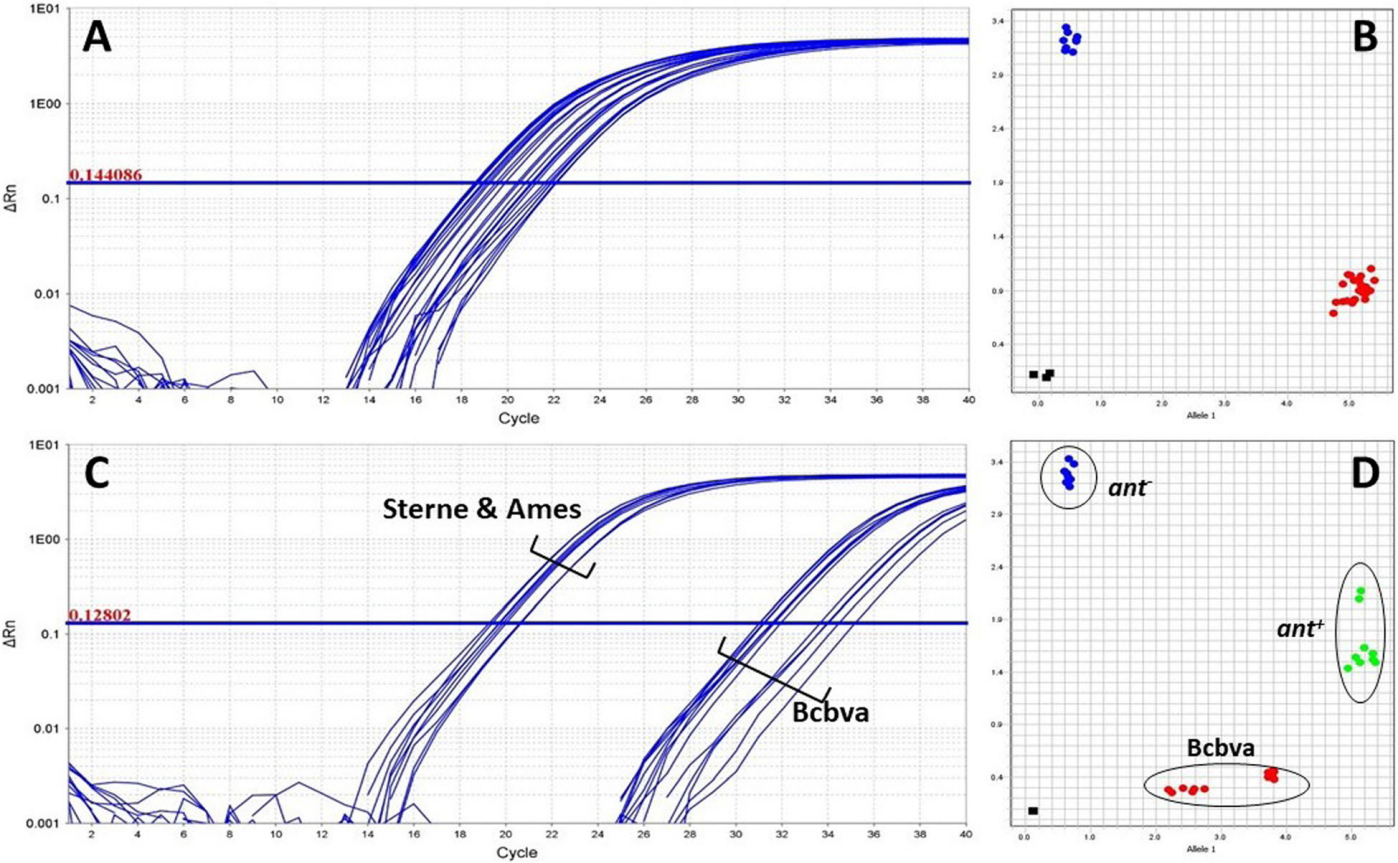
Evaluation of Bcbva isolates by rhAmp genotyping assays. Five Bcbva, three Sterne-like, and four western African isolates were tested in triplicate with ~ 1.7 × 10^5^ GE (equivalent to 1 ng of *B. anthracis* chromosomal DNA). Discrimination of western African strains remained unaltered by the presence of Bcvba in both assays and is depicted in blue along the top left corner of the *y* axis (Panels **b** and **d**). The 1352 assay produced Sterne-like amplification and discrimination of Bcbva strains (Panels **a** and **b**). Bcbva clustered with the three anthrose-positive (Sterne vaccine strain, laboratory Sterne and Ames) as illustrated by the red cluster along the *x* axis (**b**). Mutations in the primer sequences of the 892SNP, led to delayed amplification of the anthrose-producing allele in Bcbva as compared Sterne (Panel **c**). The decrease in fluorescence signal thus resulted in distinct clusters of Bcbva along the *x* axis (red dots) that were clearly separated from the higher fluorescence of the true Sterne cluster (Panel **d**). The true Sterne-like isolates, however, were mistyped as heterozygous (green cluster)

**Fig. 6:**
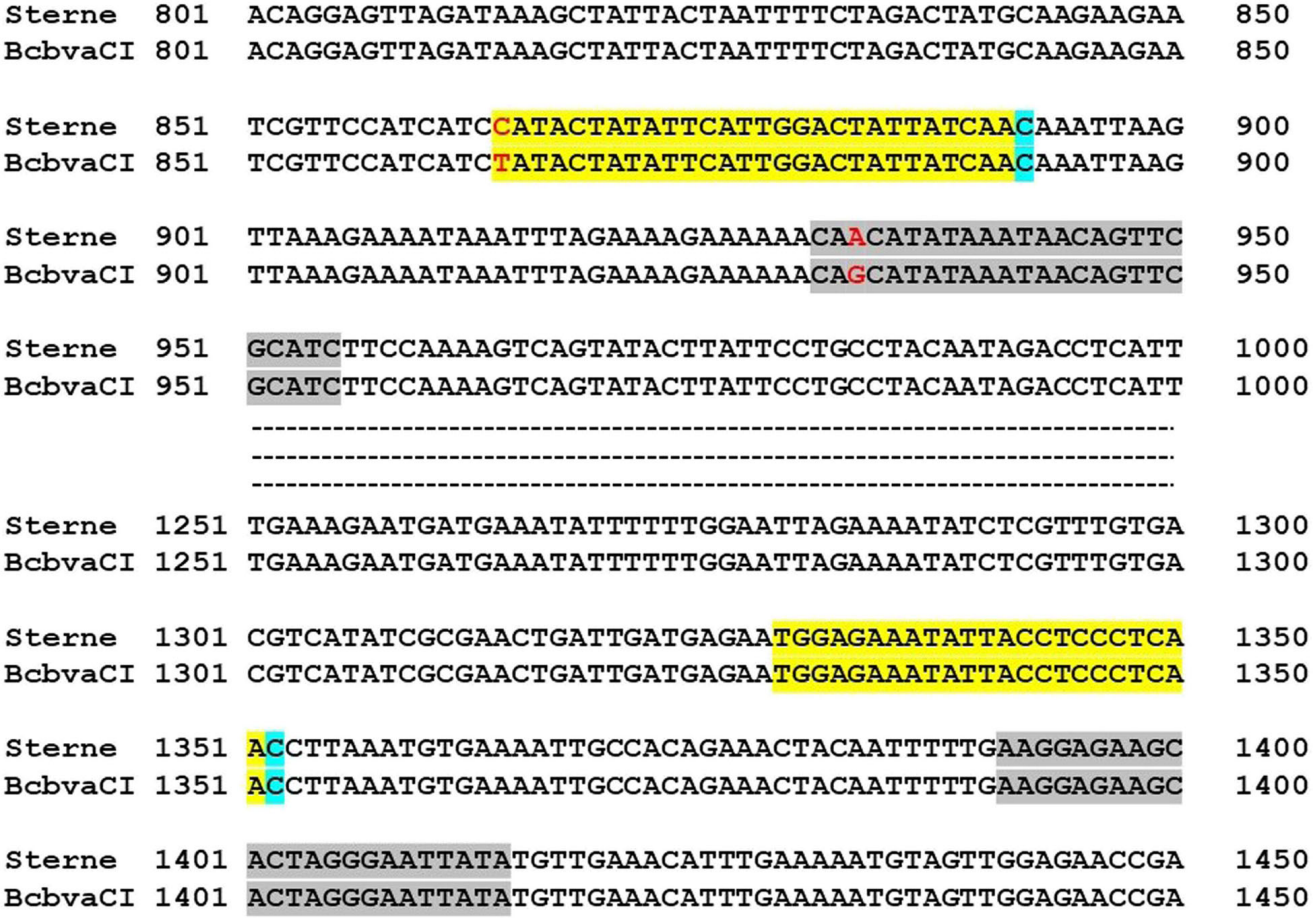
Comparison of BAS3321 rhAmp assay regions in Sterne and BcbvaCI. Forward and reverse primers developed for rhAmp assays are depicted in yellow and grey, respectively, with location of anthrose SNPs shown in blue. Two SNPs are located at the 5′ end of 892 forward primer and at position 933 of the 892 reverse primer (red). The anthrose operon and BAS3321 gene of Sterne are remarkedly conserved in Bcvba, exhibiting 99.1 and 99% identity, respectively with their homologs

Recent work in our lab identified Bcbva in the bones of deceased primates from Tai National Park in Côte d’Ivoire (Unpublished data). Given the high similarity (99.1% identity) between the Sterne anthrose operon and its homolog in the only available sequenced Bcbva strain (str. CI) [23], we investigated whether our anthrose SNP assays could be used to differentiate Bcbva from other local non-anthrose expressing *B. anthracis*. All Bcbva strains exhibited Sterne-like amplification of the 1352 SNP (Fig. 5a). Average *C*_*T*_ values for the anthrose-producing allele range from 18.7–21.5 in Bcbva and from 20 to 21 in *B. anthracis* Sterne-like strains. Accordingly, Bcbva isolates clustered with the three anthrose-positive strains tested, namely Sterne vaccine strain, laboratory Sterne, and Ames (red cluster, Fig. 5b). An alignment of the region around the 1352 SNP, including primer sequences, revealed 100% conservation between Sterne and BcbvaCI (Fig. 6).

The 892 assay was not as efficient for Bcbva. Specifically, Bcbva isolates exhibited a delayed amplification of Sterne-like alleles (Fig. 5c). Average *C*_*T*_ values ranged from 30.9 to 34.1 in Bcbva, whereas in the Sterne-like group *C*_*T*_ values did not exceed 20. The alteration in *C*_*T*_ values is likely the result of an A to G substitution at position 933 of *antB*, corresponding to the 3’end region of the 892 reverse primer sequence (Fig. 6). Bcbva was still positively genotyped for the anthrose-expressing allele, but Bcbva produced a separate and distinct cluster along the *x* axis (red clusters) that was clearly removed from the *B. anthracis* Sterne-like group (Fig. 5d). Genotyping of true anthrose positives, represented by Sterne vaccine strain, laboratory Sterne and Ames, was hindered, resulting in false heterozygous calls in repeated experiments (green cluster). Addition of a heterozygous control to the panel, composed of equal parts Sterne and Nigeria DNA, failed to resolve the true anthrose positive strains as homozygous for the anthrose-expressing allele. The C892T SNP assay is thus not well suited for discrimination of true *B. anthracis* anthrose-positive strains in the presence of Bcbva, with removal of Bcbva from the analysis resulting in correct genotype calls for that group (Data not shown).

## Discussion

This work describes the development of two SNP assays for differentiation of the *Bacillus anthracis* WAG lineage. Here we specifically show that SNPs in the BAS3321 gene of the *antABCD* operon can be used to successfully differentiate between Ant^+^ and Ant^−^ strains of *B. anthracis*. Evaluation with a globally diverse panel of isolates produced clear separation of the two genotypes. Although we tested isolates from different regions of Africa (Tanzania, Botswana, South Africa, Mozambique, Ethiopia, Namibia), and various countries around the world, only those from West Africa had the unique T892 and T1352 SNPs. While the selective pressures leading to this specific phenotype are unknown, our study suggests the anthrose-deficient genotype, whereby mutations in the *antABCD* operon prevent expression of the saccharide, appears to be limited to western Africa, as previously reported in isolates from Mali, Cameroon, Chad and Nigeria [16, 21]. The *antB* Sterne-like allele, on the other hand, is prevalent worldwide.

Interestingly, a third anthrose genotype not previously described, was also identified by this study. Specifically, an isolate from Poland did not yield amplification with either of the anthrose-positive or anthrose-negative alleles. Similarly, neither the *antC* gene nor the *antABCD* operon could be amplified in this background. Whole genome sequencing verified the conventional PCR data and showed significant variation in the genome of this isolate. Alignment of all NGS reads to Ames showed a ~ 59 kbp deletion in the genome, including the anthrose operon. It is worthwhile to note this isolate, falls within the B clade, separate from WAG (E/Aβ clade) and from Sterne and Ames, both in the A clade [26]. This genotype has not been previously reported and could constitute a distinct and novel sub-lineage of *B. anthracis*.

Lastly, we evaluated the assay in Bcbva, which also circulates in West Africa, in order to determine whether the SNPs could be used to discriminate local *B. anthracis* Ant^−^ strains from Ant^+^ Bcbva. Bcbva displays a remarkably conserved anthrose operon (99.1% homology) with Sterne. The regions around the 1352 SNP, including SNP and primer sequences, are identical to Sterne (Fig. 6). While the C892 SNP is also conserved in Bcbva, there is a C/T change at the 5′ end of the forward primer and A/G substitution within the reverse primer sequence (Fig. 6). In particular, the SNP at the 3′ end of the reverse primer is likely to have a greater adverse effect on amplification resulting in less efficient terminal extension and higher *C*_*T*_ values for the anthrose-expressing allele as compared to its counterpart in Sterne-like strains. Although the 892 assay genotypes Bcbva as anthrose positive, there is clear and reproducible separation from the Sterne cluster, and discrimination of the latter is lost. The 1352 SNP might thus be a better tool for differentiation of Bcbva from WAG isolates. This is significant as both pathogens circulate and cause anthrax across West Africa and both have poorly defined geographic distributions [21, 27]. Here we show our assay can distinguish Bcbva from WAG *B. anthracis*. Initial positive identification of Bcbva necessitates screening presumptive isolates by using the Island IV [28] and Ba-1 marker qPCR assays to differentiate Bcbva and *B. anthracis*, respectively [25]. Further qPCR assays are required to verify presence of virulence plasmids by targeting the *lef* (pXO1 and pXO1-like) and *capB* (pXO2 and pXO2-like) genes [25].

These rhAmp genotypic assays provide a quick and simple way to discriminate West African strains in the WAG lineage from other lineages and aid in the detection of local *B. anthracis* strains in West African countries. Additionally, these SNPs may provide a further tool to differentiate Bcbva, which possess anthrose 892 and 1352 Sterne-like alleles, from regional WAG *B. anthracis*. Bcbva is also reported in West Africa and can be difficult to diagnose with classical microbiology.

The significance of anthrose deficiency in a *B. anthracis* lineage that circulates in West Africa is not yet well understood. Tamborrini et al. hypothesized that the emergence of this specific phenotype in strains from Mali, Chad and Cameroon was indicative of vaccine escape mutants due to veterinary vaccination [16]. Pastoralists from Chad have reported failure of vaccination and their lessened efficacy as compared to previous years, anecdotally attributing outcomes to vaccine quality [29, 30]. While a link between vaccination failure and anthrose deficiency has not been established, studies have illustrated the strong immunogenic character of anthrose and its recognition by sera of vaccine immunized animals [11, 16]. In particular, sera from mice immunized with live or irradiated spores *B. anthracis* 34F2, the commonly used strain for livestock vaccine, recognized a synthetic anthrose-containing trisaccharide and identified a moiety of anthrose essential for this interaction [11]. Similarly, immunization of cattle with Sterne 34F2 in Chad elicited the production of IgG antibodies that recognized anthrose, the anthrose-rhamnose disaccharide and the anthrose-containing tetrasaccharide but not trirhamnose [16]. Anthrose might thus be a key spore-associated antigen, enhancing or augmenting protection afforded by protective-antigen. Livestock vaccination acting as selective pressure to eliminate anthrose is an intriguing theory that warrants further exploration.

The emergence of SNPs eliminating anthrose expression is also interesting in the context of the slow evolutionary rate of this pathogen. *B. anthracis* is considered a slow evolving pathogen that alternates between long dormant periods in the soil and short vegetative phases of about 20–40 generations [31–33]. Current work in our lab is focusing on the sequencing and characterization of the available Ant^−^ isolates.

Until recently, the geography of these Ant^*−*^ WAG *B. anthracis* and Bcbva seems restricted to areas in West and Central Africa. Classical *Bacillus anthracis* outbreaks are frequent in the region, human mortality rates are among the highest globally [34, 35], but sampling for either pathogen (*B. anthracis* or Bcbva) is limited [17, 36]. Our SNP assay targeting *ant*^−^ non-sense SNP mutations identified an additional Ant^−^ strain from the B-group isolated in Poland. Furthermore, a bioinformatics approach was used to genotype *B. anthracis* Ba4599 as Ant^*−*^*.* Ba4599 caused an anthrax outbreak among heroin users in Europe associated with unusually high mortality (28.5%). Normally < 2% mortality is seen in treated cutaneous infections [37]. The larger picture emerging is representative of several lineages of *B. anthracis* across various geographical areas undergoing convergent evolution towards anthrose deficiency. The assay developed here will aid in the discrimination of Ant^*+*^ and Ant^*−*^ *B. anthracis* in areas of both high and low surveillance. It is a cost-effective genotyping assay that is especially useful in areas with limited genome sequencing resources but qPCR capability.

## Conclusions

The assays described in this paper will allow for the prompt identification of the WAG lineage and increase our understanding of the molecular epidemiology of *B. anthracis* in western Africa. Thus far, WAG has only been identified in this region of the world. These SNPs provide a quick and useful tool for surveillance to monitor spread and prevalence of this group across the region and beyond its currently known geographic distribution. Our work here is the first to use such SNPs to differentiate WAG from other *B. anthracis* lineages, without the need for labor intense sequencing of the whole genome or variable number tandem repeat markers. This assay would be particularly useful in areas with access to a real-time PCR system but where sequencing capabilities are not readily available. In our experience, real time PCR systems are currently more widely available than sequencing capacity throughout the region.

## Methods

### Bioinformatics analyses

A total of 354 whole genome shotgun entries were located in NCBI by typing “anthracis” in the term query (https://www.ncbi.nlm.nih.gov/Traces/wgs/?page=1&view=wgs&search). Of these, 176 entries were found to be unique, nonredundant records and were selected for analysis (Additional file 1: Table S1). Forty-seven records lacking clear *B. anthracis* markers were removed [25]. Genome contigs were screened by the Basic Local Alignment Search Tool for the presence of the anthrose operon using Sterne *antABCD* as reference. DNA sequences were aligned using CLC Sequence Viewer (Qiagen).

### Bacterial strains and plasmids

In addition to screening available genomes, a diverse and globally representative panel of 49 *B. anthracis* strains from the Martin E. Hugh-Jones Collection housed at the University of Florida was examined in this study (Table 1). This panel included representatives of each major lineage as well as actively circulating strains in livestock and wildlife. *B. anthracis* Sterne 34F2 spores were obtained from Colorado Serum Company. In addition, five Bcbva strains, recently isolated in our lab from the bones of deceased primates from Tai National Park (Côte d’Ivoire), were also tested.

Anthrose-positive and anthrose-negative control plasmids were constructed. Briefly, primers antA-Up-EcoRI (5′-AAGTGAATTCGATAGGGTATTTC-3′) and antD-Dn-NheI (5′-ATAAAGCTAGCTCCTTAC- ATAATATC-3′) were used to amplify the *antABCD* operons of Sterne and Nigerian strains. The PCR was carried out in a 25-μl reaction containing 1 ng of DNA, 0.2 mM of dNTP mix, 1.5 mM of MgCl_2_, 0.2 μM of each primer, 1X of PCR buffer and 2 U of High Fidelity Platinum Taq DNA Polymerase (Invitrogen, 11,304,102). An initial denaturation at 94 °C for 2 min was followed by 30 cycles of 94 °C for 30 s, 58 °C for 30 s and 72 °C for 6.5 min DNA was analyzed by gel electrophoresis in a 0.5% agarose gel. The observed 5.5 kb band was excised and ligated to pGEM T-Easy T/A cloning vector (Promega, A1360) using T4 DNA ligase (NEB, M020) as directed by the manufacturer. The ligation was heat-shocked into DH5α chemically competent cells and selected on LB ampicillin 100 μg/ml. Plasmid insert was verified by digestion with *Eco*RI and *Nhe*I.

### DNA isolation and quantification

DNA was extracted with the DNeasy UltraClean Microbial Kit (Qiagen, 12,224–50) inside a biosafety cabinet in a biosafety level 3 laboratory. Briefly, bacterial colonies were suspended in 1.5 ml of tryptic soy broth, whereas for broth cultures 1.5 ml of the cultures were harvested. Cells were pelleted down by centrifugation and bead beat for 10 min. DNA was extracted according to the manufacturer’s instructions and sterilized by filtering through a 0.22 μm filter. DNA was quantified using the Qubit 3 fluorometer and the Qubit dsDNA BR Assay Kit (ThermoFisher, Q32850) according to the manufacturer’s protocol.

### rhAmp genotyping assays

SNPs previously reported in the BAS3321 gene of the anthrose operon were used to develop two rhAmp genotyping assays in order to distinguish between anthrose positive and negative strains of *B. anthracis*. Two forward allele-specific primers were labeled with either FAM or Yakima Yellow (YY). YY-labeled primer preferentially binds to the SNP present in anthrose positive strains, whereas FAM-labeled primers bind to the SNP in anthrose negative strains. The genotyping reaction was performed according to the manufacturer’s instructions with modifications. Briefly, 5.3 μl of combined master mix (IDT, 1076015) and reporter mix (IDT, 1076021) were mixed with 1.5 μl of 20X rhAmp SNP assay (IDT, Custom Design). DNA and water were added to a volume of 10 μl. The reaction was run in a QuantStudio 7 Flex instrument with the cycling parameters described in Table 2.

**Table 2:** Cycling parameters for rhAmp anthrose assays in QuantStudio 7 flex

Cycle	Temperature	Time	Data collection
Pre-read stage	60 °C	30 s	On
Initial denature	95 °C	3 min	Off
PCR (40 cycles)	95 °C	10 s	Off
	60 °C	30 s	Off
	68 °C	40 s	On
Post-read stage	60 °C	30 s	On

The SNP assays were used to genotype a globally diverse panel of 49 *B. anthracis* strains with 1 ng of DNA (~ 1.7 × 10^5^ GE). DNA from Sterne 34F2 spores and five Bcbva strains were also included in the study.

### Sensitivity of rhAmp genotyping assays

To establish the sensitivity of each SNP assay, serial dilutions of DNA from both an anthrose positive (Sterne) and an anthrose negative (Nigerian) strains were tested. The tests were performed in triplicate with concentrations ranging from 10 fg to 100 pg of DNA.

Anthrose-positive and -negative control plasmids carrying the *antABCD* operons of Sterne and Nigerian strains respectively, were similarly tested in triplicate. The 10-fold serial dilutions ranged from 1.77E6 to 1.77 GE of plasmid DNA, which corresponded to 10 ng through 10 fg of *B. anthracis* chromosomal DNA.

### PCR for detection of *antC* and *antABCD* operon

A strain yielding no amplification with either of the SNP assays, was checked for the presence of *antC* and the *antABCD* operon by gradient PCR. *antC* was amplified with primers antC-Comp-EcoRI (GTATAAGCTAGCTGAGAAACAAGGAATG) and antC-Comp-NheI (ATCCAGAATTCTTTAGCTCTTCTTGAC). The PCR was carried out in a 25-μl reaction containing 1 ng of DNA, 0.25 mM of dNTP mix, 3 mM of MgCl_2_, 0.25 μM of each primer, 1% DMSO, 0.5 U of Immolase DNA Polymerase and 2.5 μl of its amplification buffer (Bioline, BIO-21046). An initial denaturation at 95 °C for 10 min was followed by 30 cycles of 95 °C for 35 s, gradient for 45 s (50–57 °C, 6 temperatures), and 72 °C for 45 s, with a final extension at 72 °C for 6 min.

Primers *antA*-Up-EcoRI (5′-AAGTGAATTCGATAGGGTATTTC-3′) and *antD*-Dn-NheI (5′-ATAAAGC- TAGCTCCTTACATAATATC-3′) were used to amplify the anthrose operon. The PCR was carried out in a 25-μl reaction containing 1 ng of DNA, 0.3 mM of dNTP mix, 1.5 mM of MgCl_2_, 0.4 μM of each primer, 5 μl of 5X LongAmp Taq Buffer and 2.5 U of LongAmp Hot Start Taq DNA Polymerase (NEB, M0534S). An initial denaturation at 94 °C for 30 s was followed by 30 cycles of 94 °C for 20 s, gradient annealing from 52 to 57.2 °C for 30 s, and 72 °C for 6.5 min, with a final extension 65 °C for 10 min. DNA from Sterne was amplified in both PCRs as positive control. Amplicons were analyzed by gel electrophoresis in a 1.5% agarose gel.

## Supplementary information

**Additional file 1:** **Table S1.** Table of unique, nonredundant records of *Bacillus anthracis* genome shotgun entries from NCBI used for analysis in this study. A total of 176 records were initially included and 47 were removed for lack of clear *B. anthracis* markers (Records removed tab). Unique records appear in the "Unique *B. anthracis*" tab.

**Additional file 2:** **Figure S1.** Alignment of all *B. anthracis* anthrose operons showing 97–100% to Sterne. The anthrose operon of all sequenced *B. anthracis* strains were aligned to sequences from Sterne using CLC viewer.

**Additional file 3:** **Table S2.** Cycle threshold values with 1 and 5 ng of DNA. **Table S3.** Cycle threshold values for dilution curve with Sterne DNA.

**Additional file 4:** **Figure S2.** Real-time amplification plots of the 892 (A) and 1352 (C) rhAmp genotyping assays showing dilution curves of anthrose control plasmids. Ten-fold serial dilutions ranging from 1.77E6 to 1.77 GE of both anthrose positive and negative plasmids were tested in triplicate (only fluorescence from Sterne specific allele primer is shown). The average *C*_*T*_ values for C892T assay were as follows: 1.77E6 GE, 15.6; 1.77E5 GE, 18.9; 1.77E4 GE, 22.4; 1.77E3 GE, 25.9; 177 GE, 29.5; 17.7 GE, 33.3; and 1.77, 36.8. The standard curve displayed a slope of − 3.5578 and *R*^2^ of 0.9997. For the 1352 assay the average *C*_*T*_ values were: 1.77E6 GE, 17.1; 1.77E5 GE, 20.8; 1.77E4 GE, 24.6; 1.77E3 GE, 28.3; 177 GE, 31.9; 17.7 GE, 35.0; and 1.77 GE, 38.3. The standard curve had a slope of − 3.5433 and *R*^2^ of 0.9988. Amplification at the 1.77-GE level was not consistent in either of the assays. The corresponding endpoint allelic discrimination plots for the 892 (B) and 1352 (D) assays depict the Sterne *antABCD* operon-carrying plasmid as red clusters and the WAG anthrose operon-carrying plasmid in blue. Undetermined calls corresponding to 1.77 GE of plasmid DNA are depicted as black Xs. Black squares near the plot origin represent negative controls.

**Additional file 5:** **Figure S3.** Gradient PCR for amplification of the *antC* gene of the anthrose operon. A 1.2-kb amplicon was observed with Sterne DNA (lanes 7–12) after amplification with primers specific for the *antC* of the Sterne anthrose operon. No products were detected with DNA from UF00910 (lanes 1–6).

**Additional file 6:** **Figure S4.** Gradient PCR for amplification of the *antABCD* operon. A 5.6-kb amplicon was observed with Sterne DNA (lanes 7–12) after amplification with primers specific for the anthrose operon of Sterne. No products were detected with DNA from UF00910 (lanes 1–6).

## Data Availability

All data generated or analyzed during this study are included in this article.

## Abbreviations

Bcbva: *Bacillus cereus* biovar *anthracis*
*C*_*T*_: Cycle threshold
GE: Genome equivalents
KLH: Keyhole-limpet-hemocyanine
MLVA: Multi-locus variable number tandem repeat analysis
SNP: Single nucleotide polymorphism
WAG: West African Group
YY: Yakima Yellow
