# Supplementary material for: Nucleotide polymorphism assay for the identification of west African group Bacillus anthracis: a lineage lacking anthrose
Source: BMC Microbiol. 2020 Jan 7;20:6. doi: 10.1186/s12866-019-1693-2 (PMC6947953; doi:10.1186/s12866-019-1693-2)

```
                          20        40        60        80
NRIV01000001.1(Sterne) ATGAAAAATGAACGATTAGTAATATGCAGTAAGAAAGGGAGTAGTGCAGTCATTACAATTCAAAACCCACCAGTCAACGCTTTAAGTTTAGAAGT
AAEO01000022.1         ATGAAAAATGAACGATTAGTAATATGCAGTAAGAAAGGGAGTAGTGCAGTCATTACAATTCAAAACCCACCAGTCAACGCTTTAAGTTTAGAAGT
ALNY01000049.1         ATGAAAAATGAACGATTAGTAATATGCAGTAAGAAAGGGAGTAGTGCAGTCATTACAATTCAAAACCCACCAGTCAACGCTTTAAGTTTAGAAGT
AGQP01000002.1         ATGAAAAATGAACGATTAGTAATATGCAGTAAGAAAGGGAGTAGTGCAGTCATTACAATTCAAAACCCACCAGTCAACGCTTTAAGTTTAGAAGT
JSZS01000049.1         ATGAAAAATGAACGATTAGTAATATGCAGTAAGAAAGGGAGTAGTGCAGTCATTACAATTCAAAACCCACCAGTCAACGCTTTAAGTTTAGAAGT
JTAG01000063.1         ATGAAAAATGAACGATTAGTAATATGCAGTAAGAAAGGGAGTAGTGCAGTCATTACAATTCAAAACCCACCAGTCAACGCTTTAAGTTTAGAAGT
ABLH01000027.1         ATGAAAAATGAACGATTAGTAATATGCAGTAAGAAAGGGAGTAGTGCAGTCATTACAATTCAAAACCCACCAGTCAACGCTTTAAGTTTAGAAGT
AAEN01000017.1         ATGAAAAATGAACGATTAGTAATATGCAGTAAGAAAGGGAGTAGTGCAGTCATTACAATTCAAAACCCACCAGTCAACGCTTTAAGTTTAGAAGT
NZ_ABKG01000001.1      ATGAAAAATGAACGATTAGTAATATGCAGTAAGAAAGGGAGTAGTGCAGTCATTACAATTCAAAACCCACCAGTCAACGCTTTAAGTTTAGAAGT
QAEL01000003.1         ATGAAAAATGAACGATTAGTAATATGCAGTAAGAAAGGGAGTAGTGCAGTCATTACAATTCAAAACCCACCAGTCAACGCTTTAAGTTTAGAAGT
NZ_AAEQ01000046.1      ATGAAAAATGAACGATTAGTAATATGCAGTAAGAAAGGGAGTAGTGCAGTCATTACAATTCAAAACCCACCAGTCAACGCTTTAAGTTTAGAAGT
AMDT01000002.1         ATGAAAAATGAACGATTAGTAATATGCAGTAAGAAAGGGAGTAGTGCAGTCATTACAATTCAAAACCCACCAGTCAACGCTTTAAGTTTAGAAGT
JMPU01000016.1         ATGAAAAATGAACGATTAGTAATATGCAGTAAGAAAGGGAGTAGTGCAGTCATTACAATTCAAAACCCACCAGTCAACGCTTTAAGTTTAGAAGT
QAEM01000001.1         ATGAAAAATGAACGATTAGTAATATGCAGTAAGAAAGGGAGTAGTGCAGTCATTACAATTCAAAACCCACCAGTCAACGCTTTAAGTTTAGAAGT
QAEN01000002.1         ATGAAAAATGAACGATTAGTAATATGCAGTAAGAAAGGGAGTAGTGCAGTCATTACAATTCAAAACCCACCAGTCAACGCTTTAAGTTTAGAAGT
JHDS02000015.1         ATGAAAAATGAACGATTAGTAATATGCAGTAAGAAAGGGAGTAGTGCAGTCATTACAATTCAAAACCCACCAGTCAACGCTTTAAGTTTAGAAGT
PVLH01000002.1         ATGAAAAATGAACGATTAGTAATATGCAGTAAGAAAGGGAGTAGTGCAGTCATTACAATTCAAAACCCACCAGTCAACGCTTTAAGTT-AGAAGT
MVOA01000067.1         ATGAAAAATGAACGATTAGTAATATGCAGTAAGAAAGGGAGTAGTGCAGTCATTACAATTCAAAACCCACCAGTCAACGCTTTAAGTTTAGAAGT
QAEH01000002.1         ATGAAAAATGAACGATTAGTAATATGCAGTAAGAAAGGGAGTAGTGCAGTCATTACAATTCAAAACCCACCAGTCAACGCTTTAAGTTTAGAAGT
RQWM01000005.1         ATGAAAAATGAACGATTAGTAATATGCAGTAAGAAAGGGAGTAGTGCAGTCATTACAATTCAAAACCCACCAGTCAACGCTTTAAGTTTAGAAGT
LGCD01000038.1         ATGAAAAATGAACGATTAGTAATATGCAGTAAGAAAGGGAGTAGTGCAGTCATTACAATTCAAAACCCACCAGTCAACGCTTTAAGTTTAGAAGT
LGCC01000044.1         ACAAAAAATGAACGATTAGTAATATGCAGTAAGAAAGGGAGTAGTGCAGTCATTACAATTCAAAACCCACCAGTCAACGCTTTAAGTTTAGAAGT
Consensus              ATGAAAAATGAACGATTAGTAATATGCAGTAAGAAAGGGAGTAGTGCAGTCATTACAATTCAAAACCCACCAGTCAACGCTTTAAGTTTAGAAGT
Conservation

                          100       120       140       160       180
NRIV01000001.1(Sterne) AGTGCAACAATTAATAAATGTATTAGAGGAAATAGAGATGGACGATGATATTGCAGTCGTAATTATAACTGGAATTGGGGGGAAAGCCTTTGTAGC
AAEO01000022.1         AGTGCAACAATTAATAAATGTATTAGAGGAAATAGAGATGGACGATGATATTGCAGTCGTAATTATAACTGGAATTGGGGGGAAAGCCTTTGTAGC
ALNY01000049.1         AGTGCAACAATTAATAAATGTATTAGAGGAAATAGAGATGGACGATGATATTGCAGTCGTAATTATAACTGGAATTGGGGGGAAAGCCTTTGTAGC
AGQP01000002.1         AGTGCAACAATTAATAAATGTATTAGAGGAAATAGAGATGGACGATGATATTGCAGTCGTAATTATAACTGGAATTGGGGGGAAAGCCTTTGTAGC
JSZS01000049.1         AGTGCAACAATTAATAAATGTATTAGAGGAAATAGAGATGGACGATGATATTGCAGTCGTAATTATAACTGGAATTGGGGGGAAAGCCTTTGTAGC
JTAG01000063.1         AGTGCAACAATTAATAAATGTATTAGAGGAAATAGAGATGGACGATGATATTGCAGTCGTAATTATAACTGGAATTGGGGGGAAAGCCTTTGTAGC
ABLH01000027.1         AGTGCAACAATTAATAAATGTATTAGAGGAAATAGAGATGGACGATGATATTGCAGTCGTAATTATAACTGGAATTGGGGGGAAAGCCTTTGTAGC
AAEN01000017.1         AGTGCAACAATTAATAAATGTATTAGAGGAAATAGAGATGGACGATGATATTGCAGTCGTAATTATAACTGGAATTGGGGGGAAAGCCTTTGTAGC
NZ_ABKG01000001.1      AGTGCAACAATTAATAAATGTATTAGAGGAAATAGAGATGGACGATGATATTGCAGTCGTAATTATAACTGGAATTGGGGGGAAAGCCTTTGTAGC
QAEL01000003.1         AGTGCAACAATTAATAAATGTATTAGAGGAAATAGAGATGGACGATGATATTGCAGTCGTAATTATAACTGGAATTGGGGGGAAAGCCTTTGTAGC
NZ_AAEQ01000046.1      AGTGCAACAATTAATAAATGTATTAGAGGAAATAGAGATGGACGATGATATTGCAGTCGTAATTATAACTGGAATTGGGGGGAAAGCCTTTGTAGC
AMDT01000002.1         AGTGCAACAATTAATAAATGTATTAGAGGAAATAGAGATGGACGATGATATTGCAGTCGTAATTATAACTGGAATTGGGGGGAAAGCCTTTGTAGC
JMPU01000016.1         AGTGCAACAATTAATAAATGTATTAGAGGAAATAGAGATGGACGATGATATTGCAGTCGTAATTATAACTGGAATTGGGGGGAAAGCCTTTGTAGC
QAEM01000001.1         AGTGCAACAATTAATAAATGTATTAGAGGAAATAGAGATGGACGATGATATTGCAGTCGTAATTATAACTGGAATTGGGGGGAAAGCCTTTGTAGC
QAEN01000002.1         AGTGCAACAATTAATAAATGTATTAGAGGAAATAGAGATGGACGATGATATTGCAGTCGTAATTATAACTGGAATTGGGGGGAAAGCCTTTGTAGC
JHDS02000015.1         AGTGCAACAATTAATAAATGTATTAGAGGAAATAGAGATGGACGATGATATTGCAGTCGTAATTATAACTGGAATTGGGGGGAAAGCCTTTGTAGC
PVLH01000002.1         AGTGCAACAATTAATAAATGTATTAGAGGAAATAGAGATGGACGATGATATTGCAGTCGTAATTATAACTGGAATTGGGGGGAAAGCCTTTGTAGC
MVOA01000067.1         AGTGCAACAATTAATAAATGTATTAGAGGAAATAGAGATGGACGATGATATTGCAGTCGTAATTATAACTGGAATTGGGGGGAAAGCCTTTGTAGC
QAEH01000002.1         AGTGCAACAATTAATAAATGTATTAGAGGAAATAGAGATGGACGATGATATTGCAGTCGTAATTATAACTGGAATTGGGGGGAAAGCCTTTGTAGC
RQWM01000005.1         AGTGCAACAATTAATAAATGTATTAGAGGAAATAGAGATGGACGATGATATTGCAGTCGTAATTATAACTGGAATTGGGGGGAAAGCCTTTGTAGC
LGCD01000038.1         AGTGCAACAATTAATAAATGTATTAGAGGAAATAGAGATGGACGATGATATTGCAGTCGTAATTATAACTGGAATTGGGGGGAAAGCCTTTGTAGC
LGCC01000044.1         AGTGCAACAATTAATAAATGTATTAGAGGAAATAGAGATGGACGATGATATTGCAGTCGTAATTACAACTGGAATTGGGGGGAAAGCCTTTGTAGC
Consensus              AGTGCAACAATTAATAAATGTATTAGAGGAAATAGAGATGGACGATGATATTGCAGTCGTAATTATAACTGGAATTGGGGGGAAAGCCTTTGTAGC
Conservation

                          200       220       240       260       280
NRIV01000001.1(Sterne) TGGAGGGGATATAAAAGAATTTCCAGGTTGGATAGGAAAAGGTGAAAAATATGCTGAGATGAAATCAATAGAATTACAAAGGCCTCTTAATCAACT
AAEO01000022.1         TGGAGGGGATATAAAAGAATTTCCAGGTTGGATAGGAAAAGGTGAAAAATATGCTGAGATGAAATCAATAGAATTACAAAGGCCTCTTAATCAACT
ALNY01000049.1         TGGAGGGGATATAAAAGAATTTCCAGGTTGGATAGGAAAAGGTGAAAAATATGCTGAGATGAAATCAATAGAATTACAAAGGCCTCTTAATCAACT
AGQP01000002.1         TGGAGGGGATATAAAAGAATTTCCAGGTTGGATAGGAAAAGGTGAAAAATATGCTGAGATGAAATCAATAGAATTACAAAGGCCTCTTAATCAACT
JSZS01000049.1         TGGAGGGGATATAAAAGAATTTCCAGGTTGGATAGGAAAAGGTGAAAAATATGCTGAGATGAAATCAATAGAATTACAAAGGCCTCTTAATCAACT
JTAG01000063.1         TGGAGGGGATATAAAAGAATTTCCAGGTTGGATAGGAAAAGGTGAAAAATATGCTGAGATGAAATCAATAGAATTACAAAGGCCTCTTAATCAACT
ABLH01000027.1         TGGAGGGGATATAAAAGAATTTCCAGGTTGGATAGGAAAAGGTGAAAAATATGCTGAGATGAAATCAATAGAATTACAAAGGCCTCTTAATCAACT
AAEN01000017.1         TGGAGGGGATATAAAAGAATTTCCAGGTTGGATAGGAAAAGGTGAAAAATATGCTGAGATGAAATCAATAGAATTACAAAGGCCTCTTAATCAACT
NZ_ABKG01000001.1      TGGAGGGGATATAAAAGAATTTCCAGGTTGGATAGGAAAAGGTGAAAAATATGCTGAGATGAAATCAATAGAATTACAAAGGCCTCTTAATCAACT
QAEL01000003.1         TGGAGGGGATATAAAAGAATTTCCAGGTTGGATAGGAAAAGGTGAAAAATATGCTGAGATGAAATCAATAGAATTACAAAGGCCTCTTAATCAACT
NZ_AAEQ01000046.1      TGGAGGGGATATAAAAGAATTTCCAGGTTGGATAGGAAAAGGTGAAAAATATGCTGAGATGAAATCAATAGAATTACAAAGGCCTCTTAATCAACT
AMDT01000002.1         TGGAGGGGATATAAAAGAATTTCCAGGTTGGATAGGAAAAGGTGAAAAATATGCTGAGATGAAATCAATAGAATTACAAAGGCCTCTTAATCAACT
JMPU01000016.1         TGGAGGGGATATAAAAGAATTTCCAGGTTGGATAGGAAAAGGTGAAAAATATGCTGAGATGAAATCAATAGAATTACAAAGGCCTCTTAATCAACT
QAEM01000001.1         TGGAGGGGATATAAAAGAATTTCCAGGTTGGATAGGAAAAGGTGAAAAATATGCTGAGATGAAATCAATAGAATTACAAAGGCCTCTTAATCAACT
QAEN01000002.1         TGGAGGGGATATAAAAGAATTTCCAGGTTGGATAGGAAAAGGTGAAAAATATGCTGAGATGAAATCAATAGAATTACAAAGGCCTCTTAATCAACT
JHDS02000015.1         TGGAGGGGATATAAAAGAATTTCCAGGTTGGATAGGAAAAGGTGAAAAATATGCTGAGATGAAATCAATAGAATTACAAAGGCCTCTTAATCAACT
PVLH01000002.1         TGGAGGGGATATAAAAGAATTTCCAGGTTGGATAGGAAAAGGTGAAAAATATGCTGAGATGAAATCAATAGAATTACAAAGGCCTCTTAATCAACT
MVOA01000067.1         TGGAGGGGATATAAAAGAATTTCCAGGTTGGATAGGAAAAGGTGAAAAATATGCTGAGATGAAATCAATAGAATTACAAAGGCCTCTTAATCAACT
QAEH01000002.1         TGGAGGGGATATAAAAGAATTTCCAGGTTGGATAGGAAAAGGTGAAAAATATGCTGAGATGAAATCAATAGAATTACAAAAGCCTCTTAATCAACT
RQWM01000005.1         TGGAGGGGATATAAAAGAATTTCCAGGTTGGATAGGAAAAGGTGAAAAATATGCTGAGATGAAATCAATAGAATTACAAAGGCCTCTTAATCAACT
LGCD01000038.1         TGGAGGGGATATAAAAGAATTTCCAGGTTGGATAGGAAAAGGTGAAAAATATGCTGAGATGAAATCAATAGAATTACAAAGGCCTCTTAATCAACT
LGCC01000044.1         TGGAGGGGATATAAAAGAAATTCCAGGTCGCATAGGAAAAGGTGAAAAATATGCTGAGATGAAATCAATAGAATTACAAAGGCCTCTTAATCAACT
Consensus              TGGAGGGGATATAAAAGAATTTCCAGGTTGGATAGGAAAAGGTGAAAAATATGCTGAGATGAAATCAATAGAATTACAAAGGCCTCTTAATCAACT
Conservation
```

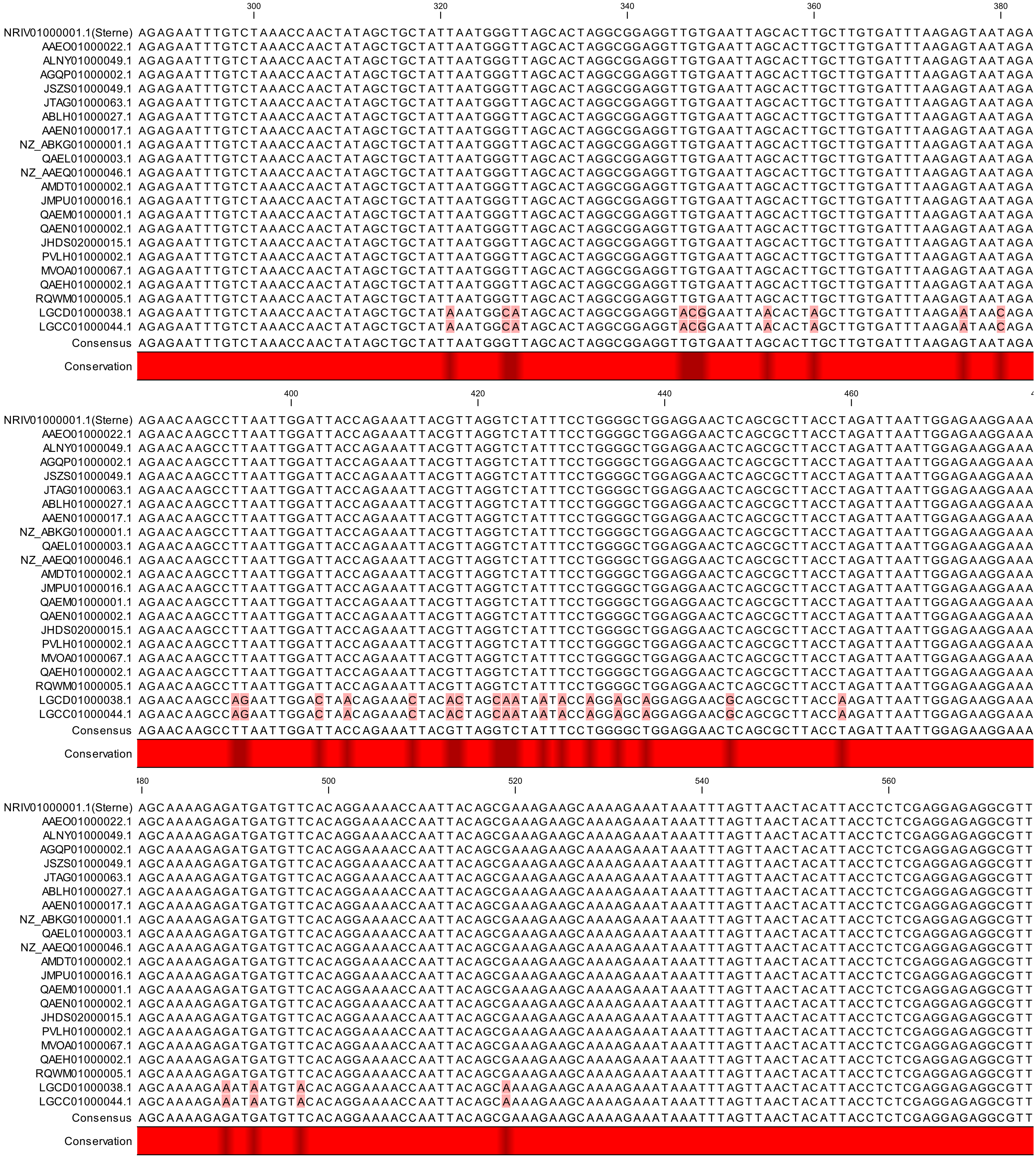
300 320 340 360 380
NRIV01000001.1(Sterne) AGAGAATTTGTCTAAACCAACTATAGCTGCTATTAATGGGTTAGCACTAGGCGGAGGTTGTGAATTAGCACTTGCTTGTGATTTAAGAGTAATAGA
AAEO01000022.1 AGAGAATTTGTCTAAACCAACTATAGCTGCTATTAATGGGTTAGCACTAGGCGGAGGTTGTGAATTAGCACTTGCTTGTGATTTAAGAGTAATAGA
ALNY01000049.1 AGAGAATTTGTCTAAACCAACTATAGCTGCTATTAATGGGTTAGCACTAGGCGGAGGTTGTGAATTAGCACTTGCTTGTGATTTAAGAGTAATAGA
AGQP01000002.1 AGAGAATTTGTCTAAACCAACTATAGCTGCTATTAATGGGTTAGCACTAGGCGGAGGTTGTGAATTAGCACTTGCTTGTGATTTAAGAGTAATAGA
JSZS01000049.1 AGAGAATTTGTCTAAACCAACTATAGCTGCTATTAATGGGTTAGCACTAGGCGGAGGTTGTGAATTAGCACTTGCTTGTGATTTAAGAGTAATAGA
JTAG01000063.1 AGAGAATTTGTCTAAACCAACTATAGCTGCTATTAATGGGTTAGCACTAGGCGGAGGTTGTGAATTAGCACTTGCTTGTGATTTAAGAGTAATAGA
ABLH01000027.1 AGAGAATTTGTCTAAACCAACTATAGCTGCTATTAATGGGTTAGCACTAGGCGGAGGTTGTGAATTAGCACTTGCTTGTGATTTAAGAGTAATAGA
AAEN01000017.1 AGAGAATTTGTCTAAACCAACTATAGCTGCTATTAATGGGTTAGCACTAGGCGGAGGTTGTGAATTAGCACTTGCTTGTGATTTAAGAGTAATAGA
NZ_ABKG01000001.1 AGAGAATTTGTCTAAACCAACTATAGCTGCTATTAATGGGTTAGCACTAGGCGGAGGTTGTGAATTAGCACTTGCTTGTGATTTAAGAGTAATAGA
QAEL01000003.1 AGAGAATTTGTCTAAACCAACTATAGCTGCTATTAATGGGTTAGCACTAGGCGGAGGTTGTGAATTAGCACTTGCTTGTGATTTAAGAGTAATAGA
NZ_AAEQ01000046.1 AGAGAATTTGTCTAAACCAACTATAGCTGCTATTAATGGGTTAGCACTAGGCGGAGGTTGTGAATTAGCACTTGCTTGTGATTTAAGAGTAATAGA
AMDT01000002.1 AGAGAATTTGTCTAAACCAACTATAGCTGCTATTAATGGGTTAGCACTAGGCGGAGGTTGTGAATTAGCACTTGCTTGTGATTTAAGAGTAATAGA
JMPU01000016.1 AGAGAATTTGTCTAAACCAACTATAGCTGCTATTAATGGGTTAGCACTAGGCGGAGGTTGTGAATTAGCACTTGCTTGTGATTTAAGAGTAATAGA
QAEM01000001.1 AGAGAATTTGTCTAAACCAACTATAGCTGCTATTAATGGGTTAGCACTAGGCGGAGGTTGTGAATTAGCACTTGCTTGTGATTTAAGAGTAATAGA
QAEN01000002.1 AGAGAATTTGTCTAAACCAACTATAGCTGCTATTAATGGGTTAGCACTAGGCGGAGGTTGTGAATTAGCACTTGCTTGTGATTTAAGAGTAATAGA
JHDS02000015.1 AGAGAATTTGTCTAAACCAACTATAGCTGCTATTAATGGGTTAGCACTAGGCGGAGGTTGTGAATTAGCACTTGCTTGTGATTTAAGAGTAATAGA
PVLH01000002.1 AGAGAATTTGTCTAAACCAACTATAGCTGCTATTAATGGGTTAGCACTAGGCGGAGGTTGTGAATTAGCACTTGCTTGTGATTTAAGAGTAATAGA
MVOA01000067.1 AGAGAATTTGTCTAAACCAACTATAGCTGCTATTAATGGGTTAGCACTAGGCGGAGGTTGTGAATTAGCACTTGCTTGTGATTTAAGAGTAATAGA
QAEH01000002.1 AGAGAATTTGTCTAAACCAACTATAGCTGCTATTAATGGGTTAGCACTAGGCGGAGGTTGTGAATTAGCACTTGCTTGTGATTTAAGAGTAATAGA
RQWM01000005.1 AGAGAATTTGTCTAAACCAACTATAGCTGCTATTAATGGGTTAGCACTAGGCGGAGGTTGTGAATTAGCACTTGCTTGTGATTTAAGAGTAATAGA
LGCD01000038.1 AGAGAATTTGTCTAAACCAACTATAGCTGCTATAAATGGCATAGCACTAGGCGGAGGTACGGAATTAACACTAGCTTGTGATTTAAGAATAACAGA
LGCC01000044.1 AGAGAATTTGTCTAAACCAACTATAGCTGCTATAAATGGCATAGCACTAGGCGGAGGTACGGAATTAACACTAGCTTGTGATTTAAGAATAACAGA
Consensus AGAGAATTTGTCTAAACCAACTATAGCTGCTATTAATGGGTTAGCACTAGGCGGAGGTTGTGAATTAGCACTTGCTTGTGATTTAAGAGTAATAGA
Conservation
400 420 440 460
NRIV01000001.1(Sterne) AGAACAAGCCTTAATTGGATTACCAGAAATTACGTTAGGTCTATTTCCTGGGGCTGGAGGAACTCAGCGCTTACCTAGATTAATTGGAGAAGGAAA
AAEO01000022.1 AGAACAAGCCTTAATTGGATTACCAGAAATTACGTTAGGTCTATTTCCTGGGGCTGGAGGAACTCAGCGCTTACCTAGATTAATTGGAGAAGGAAA
ALNY01000049.1 AGAACAAGCCTTAATTGGATTACCAGAAATTACGTTAGGTCTATTTCCTGGGGCTGGAGGAACTCAGCGCTTACCTAGATTAATTGGAGAAGGAAA
AGQP01000002.1 AGAACAAGCCTTAATTGGATTACCAGAAATTACGTTAGGTCTATTTCCTGGGGCTGGAGGAACTCAGCGCTTACCTAGATTAATTGGAGAAGGAAA
JSZS01000049.1 AGAACAAGCCTTAATTGGATTACCAGAAATTACGTTAGGTCTATTTCCTGGGGCTGGAGGAACTCAGCGCTTACCTAGATTAATTGGAGAAGGAAA
JTAG01000063.1 AGAACAAGCCTTAATTGGATTACCAGAAATTACGTTAGGTCTATTTCCTGGGGCTGGAGGAACTCAGCGCTTACCTAGATTAATTGGAGAAGGAAA
ABLH01000027.1 AGAACAAGCCTTAATTGGATTACCAGAAATTACGTTAGGTCTATTTCCTGGGGCTGGAGGAACTCAGCGCTTACCTAGATTAATTGGAGAAGGAAA
AAEN01000017.1 AGAACAAGCCTTAATTGGATTACCAGAAATTACGTTAGGTCTATTTCCTGGGGCTGGAGGAACTCAGCGCTTACCTAGATTAATTGGAGAAGGAAA
NZ_ABKG01000001.1 AGAACAAGCCTTAATTGGATTACCAGAAATTACGTTAGGTCTATTTCCTGGGGCTGGAGGAACTCAGCGCTTACCTAGATTAATTGGAGAAGGAAA
QAEL01000003.1 AGAACAAGCCTTAATTGGATTACCAGAAATTACGTTAGGTCTATTTCCTGGGGCTGGAGGAACTCAGCGCTTACCTAGATTAATTGGAGAAGGAAA
NZ_AAEQ01000046.1 AGAACAAGCCTTAATTGGATTACCAGAAATTACGTTAGGTCTATTTCCTGGGGCTGGAGGAACTCAGCGCTTACCTAGATTAATTGGAGAAGGAAA
AMDT01000002.1 AGAACAAGCCTTAATTGGATTACCAGAAATTACGTTAGGTCTATTTCCTGGGGCTGGAGGAACTCAGCGCTTACCTAGATTAATTGGAGAAGGAAA
JMPU01000016.1 AGAACAAGCCTTAATTGGATTACCAGAAATTACGTTAGGTCTATTTCCTGGGGCTGGAGGAACTCAGCGCTTACCTAGATTAATTGGAGAAGGAAA
QAEM01000001.1 AGAACAAGCCTTAATTGGATTACCAGAAATTACGTTAGGTCTATTTCCTGGGGCTGGAGGAACTCAGCGCTTACCTAGATTAATTGGAGAAGGAAA
QAEN01000002.1 AGAACAAGCCTTAATTGGATTACCAGAAATTACGTTAGGTCTATTTCCTGGGGCTGGAGGAACTCAGCGCTTACCTAGATTAATTGGAGAAGGAAA
JHDS02000015.1 AGAACAAGCCTTAATTGGATTACCAGAAATTACGTTAGGTCTATTTCCTGGGGCTGGAGGAACTCAGCGCTTACCTAGATTAATTGGAGAAGGAAA
PVLH01000002.1 AGAACAAGCCTTAATTGGATTACCAGAAATTACGTTAGGTCTATTTCCTGGGGCTGGAGGAACTCAGCGCTTACCTAGATTAATTGGAGAAGGAAA
MVOA01000067.1 AGAACAAGCCTTAATTGGATTACCAGAAATTACGTTAGGTCTATTTCCTGGGGCTGGAGGAACTCAGCGCTTACCTAGATTAATTGGAGAAGGAAA
QAEH01000002.1 AGAACAAGCCTTAATTGGATTACCAGAAATTACGTTAGGTCTATTTCCTGGGGCTGGAGGAACTCAGCGCTTACCTAGATTAATTGGAGAAGGAAA
RQWM01000005.1 AGAACAAGCCTTAATTGGATTACCAGAAATTACGTTAGGTCTATTTCCTGGGGCTGGAGGAACTCAGCGCTTACCTAGATTAATTGGAGAAGGAAA
LGCD01000038.1 AGAACAAGCCAGAATTGGACTAACAGAAACTACACTAGCAATAATACCAGGAGCAGGAGGAACGCAGCGCTTACCAAGATTAATTGGAGAAGGAAA
LGCC01000044.1 AGAACAAGCCAGAATTGGACTAACAGAAACTACACTAGCAATAATACCAGGAGCAGGAGGAACGCAGCGCTTACCAAGATTAATTGGAGAAGGAAA
Consensus AGAACAAGCCTTAATTGGATTACCAGAAATTACGTTAGGTCTATTTCCTGGGGCTGGAGGAACTCAGCGCTTACCTAGATTAATTGGAGAAGGAAA
Conservation
480 500 520 540 560
NRIV01000001.1(Sterne) AGCAAAAGAGATGATGTTCACAGGAAAACCAATTACAGCGAAAGAAGCAAAAGAAATAAATTTAGTTAACTACATTACCTCTCGAGGAGAGGCGTT
AAEO01000022.1 AGCAAAAGAGATGATGTTCACAGGAAAACCAATTACAGCGAAAGAAGCAAAAGAAATAAATTTAGTTAACTACATTACCTCTCGAGGAGAGGCGTT
ALNY01000049.1 AGCAAAAGAGATGATGTTCACAGGAAAACCAATTACAGCGAAAGAAGCAAAAGAAATAAATTTAGTTAACTACATTACCTCTCGAGGAGAGGCGTT
AGQP01000002.1 AGCAAAAGAGATGATGTTCACAGGAAAACCAATTACAGCGAAAGAAGCAAAAGAAATAAATTTAGTTAACTACATTACCTCTCGAGGAGAGGCGTT
JSZS01000049.1 AGCAAAAGAGATGATGTTCACAGGAAAACCAATTACAGCGAAAGAAGCAAAAGAAATAAATTTAGTTAACTACATTACCTCTCGAGGAGAGGCGTT
JTAG01000063.1 AGCAAAAGAGATGATGTTCACAGGAAAACCAATTACAGCGAAAGAAGCAAAAGAAATAAATTTAGTTAACTACATTACCTCTCGAGGAGAGGCGTT
ABLH01000027.1 AGCAAAAGAGATGATGTTCACAGGAAAACCAATTACAGCGAAAGAAGCAAAAGAAATAAATTTAGTTAACTACATTACCTCTCGAGGAGAGGCGTT
AAEN01000017.1 AGCAAAAGAGATGATGTTCACAGGAAAACCAATTACAGCGAAAGAAGCAAAAGAAATAAATTTAGTTAACTACATTACCTCTCGAGGAGAGGCGTT
NZ_ABKG01000001.1 AGCAAAAGAGATGATGTTCACAGGAAAACCAATTACAGCGAAAGAAGCAAAAGAAATAAATTTAGTTAACTACATTACCTCTCGAGGAGAGGCGTT
QAEL01000003.1 AGCAAAAGAGATGATGTTCACAGGAAAACCAATTACAGCGAAAGAAGCAAAAGAAATAAATTTAGTTAACTACATTACCTCTCGAGGAGAGGCGTT
NZ_AAEQ01000046.1 AGCAAAAGAGATGATGTTCACAGGAAAACCAATTACAGCGAAAGAAGCAAAAGAAATAAATTTAGTTAACTACATTACCTCTCGAGGAGAGGCGTT
AMDT01000002.1 AGCAAAAGAGATGATGTTCACAGGAAAACCAATTACAGCGAAAGAAGCAAAAGAAATAAATTTAGTTAACTACATTACCTCTCGAGGAGAGGCGTT
JMPU01000016.1 AGCAAAAGAGATGATGTTCACAGGAAAACCAATTACAGCGAAAGAAGCAAAAGAAATAAATTTAGTTAACTACATTACCTCTCGAGGAGAGGCGTT
QAEM01000001.1 AGCAAAAGAGATGATGTTCACAGGAAAACCAATTACAGCGAAAGAAGCAAAAGAAATAAATTTAGTTAACTACATTACCTCTCGAGGAGAGGCGTT
QAEN01000002.1 AGCAAAAGAGATGATGTTCACAGGAAAACCAATTACAGCGAAAGAAGCAAAAGAAATAAATTTAGTTAACTACATTACCTCTCGAGGAGAGGCGTT
JHDS02000015.1 AGCAAAAGAGATGATGTTCACAGGAAAACCAATTACAGCGAAAGAAGCAAAAGAAATAAATTTAGTTAACTACATTACCTCTCGAGGAGAGGCGTT
PVLH01000002.1 AGCAAAAGAGATGATGTTCACAGGAAAACCAATTACAGCGAAAGAAGCAAAAGAAATAAATTTAGTTAACTACATTACCTCTCGAGGAGAGGCGTT
MVOA01000067.1 AGCAAAAGAGATGATGTTCACAGGAAAACCAATTACAGCGAAAGAAGCAAAAGAAATAAATTTAGTTAACTACATTACCTCTCGAGGAGAGGCGTT
QAEH01000002.1 AGCAAAAGAGATGATGTTCACAGGAAAACCAATTACAGCGAAAGAAGCAAAAGAAATAAATTTAGTTAACTACATTACCTCTCGAGGAGAGGCGTT
RQWM01000005.1 AGCAAAAGAGATGATGTTCACAGGAAAACCAATTACAGCGAAAGAAGCAAAAGAAATAAATTTAGTTAACTACATTACCTCTCGAGGAGAGGCGTT
LGCD01000038.1 AGCAAAAGAAATAATGTACACAGGAAAACCAATTACAGCAAAAGAAGCAAAAGAAATAAATTTAGTTAACTACATTACCTCTCGAGGAGAGGCGTT
LGCC01000044.1 AGCAAAAGAAATAATGTACACAGGAAAACCAATTACAGCAAAAGAAGCAAAAGAAATAAATTTAGTTAACTACATTACCTCTCGAGGAGAGGCGTT
Consensus AGCAAAAGAGATGATGTTCACAGGAAAACCAATTACAGCGAAAGAAGCAAAAGAAATAAATTTAGTTAACTACATTACCTCTCGAGGAGAGGCGTT
Conservation

```
                       580                 600                 620                 640                 660
NRIV01000001.1(Sterne) AAATAAAGCAAAAGAGATTGCTAAAGATATAAGTGAATTTTCTCTTCCAGCACTTTCCTATATGAAGTTAGCAATTCGTGAAGGTTTGGCAGTACC
AAEO01000022.1         AAATAAAGCAAAAGAGATTGCTAAAGATATAAGTGAATTTTCTCTTCCAGCACTTTCCTATATGAAGTTAGCAATTCGTGAAGGTTTGGCAGTACC
ALNY01000049.1         AAATAAAGCAAAAGAGATTGCTAAAGATATAAGTGAATTTTCTCTTCCAGCACTTTCCTATATGAAGTTAGCAATTCGTGAAGGTTTGGCAGTACC
AGQP01000002.1         AAATAAAGCAAAAGAGATTGCTAAAGATATAAGTGAATTTTCTCTTCCAGCACTTTCCTATATGAAGTTAGCAATTCGTGAAGGTTTGGCAGTACC
JSZS01000049.1         AAATAAAGCAAAAGAGATTGCTAAAGATATAAGTGAATTTTCTCTTCCAGCACTTTCCTATATGAAGTTAGCAATTCGTGAAGGTTTGGCAGTACC
JTAG01000063.1         AAATAAAGCAAAAGAGATTGCTAAAGATATAAGTGAATTTTCTCTTCCAGCACTTTCCTATATGAAGTTAGCAATTCGTGAAGGTTTGGCAGTACC
ABLH01000027.1         AAATAAAGCAAAAGAGATTGCTAAAGATATAAGTGAATCTTCTCTTCCAGCACTTTCCTATATGAAGTTAGCAATTCGTGAAGGTTTGGCAGTACC
AAEN01000017.1         AAATAAAGCAAAAGAGATTGCTAAAGATATAAGTGAATCTTCTCTTCCAGCACTTTCCTATATGAAGTTAGCAATTCGTGAAGGTTTGGCAGTACC
NZ_ABKG01000001.1      AAATAAAGCAAAAGAGATTGCTAAAGATATAAGTGAATCTTCTCTTCCAGCACTTTCCTATATGAAGTTAGCAATTCGTGAAGGTTTGGCAGTACC
QAEL01000003.1         AAATAAAGCAAAAGAGATTGCTAAAGATATAAGTGAATCTTCTCTTCCAGCACTTTCCTATATGAAGTTAGCAATTCGTGAAGGTTTGGCAGTACC
NZ_AAEQ01000046.1      AAATAAAGCAAAAGAGATTGCTAAAGATATAAGTGAATCTTCTCTTCCAGCACTTTCCTATATGAAGTTAGCAATTCGTGAAGGTTTGGCAGTACC
AMDT01000002.1         AAATAAAGCAAAAGAGATTGCTAAAGATATAAGTGAATCTTCTCTTCCAGCACTTTCCTATATGAAGTTAGCAATTCGTGAAGGTTTGGCAGTACC
JMPU01000016.1         AAATAAAGCAAAAGAGATTGCTAAAGATATAAGTGAATCTTCTCTTCCAGCACTTTCCTATATGAAGTTAGCAATTCGTGAAGGTTTGGCAGTACC
QAEM01000001.1         AAATAAAGCAAAAGAGATTGCTAAAGATATAAGTGAATCTTCTCTTCCAGCACTTTCCTATATGAAGTTAGCAATTCGTGAAGGTTTGGCAGTACC
QAEN01000002.1         AAATAAAGCAAAAGAGATTGCTAAAGATATAAGTGAATCTTCTCTTCCAGCACTTTCCTATATGAAGTTAGCAATTCGTGAAGGTTTGGCAGTACC
JHDS02000015.1         AAATAAAGCAAAAGAGATTGCTAAAGATATAAGTGAATCTTCTCTTCCAGCACTTTCCTATATGAAGTTAGCAATTCGTGAAGGTTTGGCAGTACC
PVLH01000002.1         AAATAAAGCAAAAGAGATTGCTAAAGATATAAGTGAATTTTCTCTTCCAGCACTTTCCTATATGAAGTTAGCAATTCGTGAAGGTTTGGCAGTACC
MVOA01000067.1         AAATAAAGCAAAAGAGATTGCTAAAGATATAAGTGAATTTTCTCTTCCAGCACTTTCCTATATGAAGTTAGCAATTCGTGAAGGTTTGGCAGTACC
QAEH01000002.1         AAATAAAGCAAAAGAGATTGCTAAAGATATAAGTGAATTTTCTCTTCCAGCACTTTCCTATATGAAGTTAGCAATTCGTGAAGGTTTGGCAGTACC
RQWM01000005.1         AAATAAAGCAAAAGAGATTGCTAAAGATATAAGTGAATTTTCTCTTCCAGCACTTTCCTATATGAAGTTAGCAATTCGTGAAGGTTTGGCAGTACC
LGCD01000038.1         AAATAAAGCAAAAGAGATTGCTAAAGATATAAGTGAATTTTCTCTTCCAGCACTTTCCTATATGAAGTTAGCAATTCGTGAAGGTTTGGCAGTACC
LGCC01000044.1         AAATAAAGCAAAAGAGATTGCTAAAGATATAAGTGAATTTTCTCTTCCAGCACTTTCCTATATGAAGTTAGCAATTCGTGAAGGTTTGGCAGTACC
Consensus              AAATAAAGCAAAAGAGATTGCTAAAGATATAAGTGAATTTTCTCTTCCAGCACTTTCCTATATGAAGTTAGCAATTCGTGAAGGTTTGGCAGTACC
Conservation
```

```
                          680                 700                 720                 740                 760
NRIV01000001.1(Sterne) TTTACAAGAAGGGCTTCAGATCGAAGCGCGTTATTTTGGGAAAGTGTTTCAAACAGAAGATGTGAAGGAGGGGGTAAAGGCATTTATTGAAAAAAG
AAEO01000022.1         TTTACAAGAAGGGCTTCAGATCGAAGCGCGTTATTTTGGGAAAGTGTTTCAAACAGAAGATGTGAAGGAGGGGGTAAAGGCATTTATTGAAAAAAG
ALNY01000049.1         TTTACAAGAAGGGCTTCAGATCGAAGCGCGTTATTTTGGGAAAGTGTTTCAAACAGAAGATGTGAAGGAGGGGGTAAAGGCATTTATTGAAAAAAG
AGQP01000002.1         TTTACAAGAAGGGCTTCAGATCGAAGCGCGTTATTTTGGGAAAGTGTTTCAAACAGAAGATGTGAAGGAGGGGGTAAAGGCATTTATTGAAAAAAG
JSZS01000049.1         TTTACAAGAAGGGCTTCAGATCGAAGCGCGTTATTTTGGGAAAGTGTTTCAAACAGAAGATGTGAAGGAGGGGGTAAAGGCATTTATTGAAAAAAG
JTAG01000063.1         TTTACAAGAAGGGCTTCAGATCGAAGCGCGTTATTTTGGGAAAGTGTTTCAAACAGAAGATGTGAAGGAGGGGGTAAAGGCATTTATTGAAAAAAG
ABLH01000027.1         TTTACAAGAAGGGCTTCAGATCGAAGCGCGTTATTTTGGGAAAGTGTTTCAAACAGAAGATGTGAAGGAGGGGGTAAAGGCATTTATTGAAAAAAG
AAEN01000017.1         TTTACAAGAAGGGCTTCAGATCGAAGCGCGTTATTTTGGGAAAGTGTTTCAAACAGAAGATGTGAAGGAGGGGGTAAAGGCATTTATTGAAAAAAG
NZ_ABKG01000001.1      TTTACAAGAAGGGCTTCAGATCGAAGCGCGTTATTTTGGGAAAGTGTTTCAAACAGAAGATGTGAAGGAGGGGGTAAAGGCATTTATTGAAAAAAG
QAEL01000003.1         TTTACAAGAAGGGCTTCAGATCGAAGCGCGTTATTTTGGGAAAGTGTTTCAAACAGAAGATGTGAAGGAGGGGGTAAAGGCATTTATTGAAAAAAG
NZ_AAEQ01000046.1      TTTACAAGAAGGGCTTCAGATCGAAGCGCGTTATTTTGGGAAAGTGTTTCAAACAGAAGATGTGAAGGAGGGGGTAAAGGCATTTATTGAAAAAAG
AMDT01000002.1         TTTACAAGAAGGGCTTCAGATCGAAGCGCGTTATTTTGGGAAAGTGTTTCAAACAGAAGATGTGAAGGAGGGGGTAAAGGCATTTATTGAAAAAAG
JMPU01000016.1         TTTACAAGAAGGGCTTCAGATCGAAGCGCGTTATTTTGGGAAAGTGTTTCAAACAGAAGATGTGAAGGAGGGGGTAAAGGCATTTATTGAAAAAAG
QAEM01000001.1         TTTACAAGAAGGGCTTCAGATCGAAGCGCGTTATTTTGGGAAAGTGTTTCAAACAGAAGATGTGAAGGAGGGGGTAAAGGCATTTATTGAAAAAAG
QAEN01000002.1         TTTACAAGAAGGGCTTCAGATCGAAGCGCGTTATTTTGGGAAAGTGTTTCAAACAGAAGATGTGAAGGAGGGGGTAAAGGCATTTATTGAAAAAAG
JHDS02000015.1         TTTACAAGAAGGGCTTCAGATCGAAGCGCGTTATTTTGGGAAAGTGTTTCAAACAGAAGATGTGAAGGAGGGGGTAAAGGCATTTATTGAAAAAAG
PVLH01000002.1         TTTACAAGAAGGGCTTCAGATCGAAGCGCGTTATTTTGGGAAAGTGTTTCAAACAGAAGATGTGAAGGAGGGGGTAAAGGCATTTATTGAAAAAAG
MVOA01000067.1         TTTACAAGAAGGGCTTCAGATCGAAGCGCGTTATTTTGGGAAAGTGTTTCAAACAGAAGATGTGAAGGAGGGGGTAAAGGCATTTATTGAAAAAAG
QAEH01000002.1         TTTACAAGAAGGGCTTCAGATCGAAGCGCGTTATTTTGGAAAAGTGTTTCAAACAGAAGATGTGAAGGAGGGGGTAAAGGCATTTATTGAAAAAAG
RQWM01000005.1         TTTACAAGAAGGGCTTCAGATCGAAGCGCGTTATTTTGGGAAAGTGTTTCAAACAGAAGATGTGAAGGAGGGGGTAAAGGCATTTATTGAAAAAAG
LGCD01000038.1         TTTACAAGAAGGGCTTCAGATCGAAGCGCGTTATTTTGGGAAAGTGTTTCAAACAGAAGATGTGAAGGAGGGGGTAAAGGCATTTATTGAAAAAAG
LGCC01000044.1         TTTACAAGAAGGGCTTCAGATCGAAGCGCGTTATTTTGGGAAAGTGTTTCAAACAGAAGATGTGAAGGAGGGGGTAAAGGCATTTATTGAAAAAAG
Consensus              TTTACAAGAAGGGCTTCAGATCGAAGCGCGTTATTTTGGGAAAGTGTTTCAAACAGAAGATGTGAAGGAGGGGGTAAAGGCATTTATTGAAAAAAG
Conservation
```

```
                               780                 800                 820                 840                 860
NRIV01000001.1(Sterne) AGTACCGCGTTTCACCAATAAATAAAAAA-GTATTGGGGTGTATTTTACAATTGCCCACACAGATGCTACTTTTAAAGCTATTTATTTGATTCTTA
AAEO01000022.1         AGTACCGCGTTTCACCAATAAATAAAAAA-GTATTGGGGTGTATTTTACAATTGCCCACACAGATGCTACTTTTAAAGCTATTTATTTGATTCTTA
ALNY01000049.1         AGTACCGCGTTTCACCAATAAATAAAAAA-GTATTGGGGTGTATTTTACAATTGCCCACACAGATGCTACTTTTAAAGCTATTTATTTGATTCTTA
AGQP01000002.1         AGTACCGCGTTTCACCAATAAATAAAAAA-GTATTGGGGTGTATTTTACAATTGCCCACACAGATGCTACTTTTAAAGCTATTTATTTGATTCTTA
JSZS01000049.1         AGTACCGCGTTTCACCAATAAATAAAAAA-GTATTGGGGTGTATTTTACAATTGCCCACACAGATGCTACTTTTAAAGCTATTTATTTGATTCTTA
JTAG01000063.1         AGTACCGCGTTTCACCAATAAATAAAAAA-GTATTGGGGTGTATTTTACAATTGCCCACACAGATGCTACTTTTAAAGCTATTTATTTGATTCTTA
ABLH01000027.1         AGTACCGCGTTTCACCAATAAATAAAAAA-GTATTGGGGTGTATTTTACAATTGCCCACACAGATGCTACTTTTAAAGCTATTTATTTGATTCTTA
AAEN01000017.1         AGTACCGCGTTTCACCAATAAATAAAAAA-GTATTGGGGTGTATTTTACAATTGCCCACACAGATGCTACTTTTAAAGCTATTTATTTGATTCTTA
NZ_ABKG01000001.1      AGTACCGCGTTTCACCAATAAATAAAAAA-GTATTGGGGTGTATTTTACAATTGCCCACACAGATGCTACTTTTAAAGCTATTTATTTGATTCTTA
QAEL01000003.1         AGTACCGCGTTTCACCAATAAATAAAAAA-GTATTGGGGTGTATTTTACAATTGCCCACACAGATGCTACTTTTAAAGCTATTTATTTGATTCTTA
NZ_AAEQ01000046.1      AGTACCGCGTTTCACCAATAAATAAAAAA-GTATTGGGGTGTATTTTACAATTGCCCACACAGATGCTACTTTTAAAGCTATTTATTTGATTCTTA
AMDT01000002.1         AGTACCGCGTTTCACCAATAAATAAAAAA-GTATTGGGGTGTATTTTACAATTGCCCACACAGATGCTACTTTTAAAGCTATTTATTTGATTCTTA
JMPU01000016.1         AGTACCGCGTTTCACCAATAAATAAAAAA-GTATTGGGGTGTATTTTACAATTGCCCACACAGATGCTACTTTTAAAGCTATTTATTTGATTCTTA
QAEM01000001.1         AGTACCGCGTTTCACCAATAAATAAAAAA-GTATTGGGGTGTATTTTACAATTGCCCACACAGATGCTACTTTTAAAGCTATTTATTTGATTCTTA
QAEN01000002.1         AGTACCGCGTTTCACCAATAAATAAAAAA-GTATTGGGGTGTATTTTACAATTGCCCACACAGATGCTACTTTTAAAGCTATTTATTTGATTCTTA
JHDS02000015.1         AGTACCGCGTTTCACCAATAAATAAAAAA-GTATTGGGGTGTATTTTACAATTGCCCACACAGATGCTACTTTTAAAGCTATTTATTTGATTCTTA
PVLH01000002.1         AGTACCGCGTTTCACCAATAAATAAAAAA-GTATTGGGGTGTATTTTACAATTGCCCACACAGATGCTACTTTTAAAGCTATTTATTTGATTCTTA
MVOA01000067.1         AGTACCGCGTTTCACCAATAAATAAAAAA-GTATTGGGGTGTATTTTACAATTGCCCACACAGATGCTACTTTTAAAGCTATTTATTTGATTCTTA
QAEH01000002.1         AGTACCGCGTTTCACCAATAAATAAAAAA-GCATTGGGGTGTATTTTACAATTGCCCACACAGATGCTACTTTTAAAGCTATTTATTTGATTCTTA
RQWM01000005.1         AGTACCGCGTTTCACCAATAAATAAAAAAAGTATTGGGGTGTATTTTACAATTGCCCACACAGATGCTACTTTTAAAGCTATTTATTTGATTCTTA
LGCD01000038.1         AGTACCGCGTTTCACCAATAAATAAAAAA-GTATTGGGGTGTATTTTACAATTGCCCACACAGATGCTACTTTTAAAGCTATTTATTTGATTCTTA
LGCC01000044.1         AGTACCGCGTTTCACCAATAAATAAAAAA-GTATTGGGGTGTATTTTACAATTGCCCACACAGATGCTACTTTTAAAGCTATTTATTTGATTCTTA
Consensus              AGTACCGCGTTTCACCAATAAATAAAAAA-GTATTGGGGTGTATTTTACAATTGCCCACACAGATGCTACTTTTAAAGCTATTTATTTGATTCTTA
Conservation
```

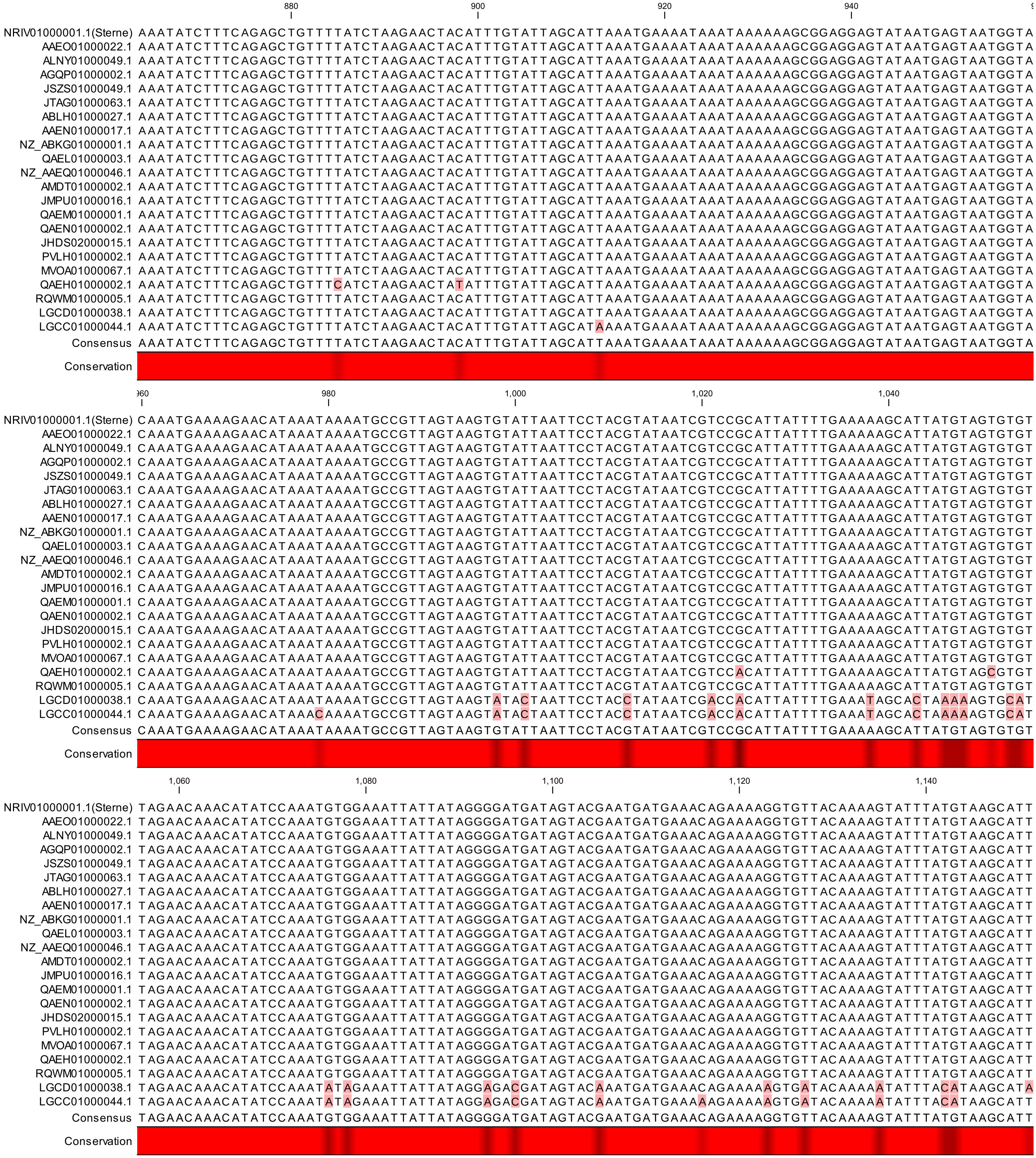
880
900
920
940
960
980
1,000
1,020
1,040
1,060
1,080
1,100
1,120
1,140
NRIV01000001.1(Sterne)
AAEO01000022.1
ALNY01000049.1
AGQP01000002.1
JSZS01000049.1
JTAG01000063.1
ABLH01000027.1
AAEN01000017.1
NZ_ABKG01000001.1
QAEL01000003.1
NZ_AAEQ01000046.1
AMDT01000002.1
JMPU01000016.1
QAEM01000001.1
QAEN01000002.1
JHDS02000015.1
PVLH01000002.1
MVOA01000067.1
QAEH01000002.1
RQWM01000005.1
LGCD01000038.1
LGCC01000044.1
Consensus
Conservation

```
                              1,160               1,180               1,200               1,220               1,240
NRIV01000001.1(Sterne) CAAATATTATCTATATAAAAAAATAGATCAACTTTTGGACAATTTGAAAATGCACTTATGTTATTGAATAAGGCGAACGGTGAATATATAAATTTCT
AAEO01000022.1         CAAATATTATCTATATAAAAAAATAGATCAACTTTTGGACAATTTGAAAATGCACTTATGTTATTGAATAAGGCGAACGGTGAATATATAAATTTCT
ALNY01000049.1         CAAATATTATCTATATAAAAAAATAGATCAACTTTTGGACAATTTGAAAATGCACTTATGTTATTGAATAAGGCGAACGGTGAATATATAAATTTCT
AGQP01000002.1         CAAATATTATCTATATAAAAAAATAGATCAACTTTTGGACAATTTGAAAATGCACTTATGTTATTGAATAAGGCGAACGGTGAATATATAAATTTCT
JSZS01000049.1         CAAATATTATCTATATAAAAAAATAGATCAACTTTTGGACAATTTGAAAATGCACTTATGTTATTGAATAAGGCGAACGGTGAATATATAAATTTCT
JTAG01000063.1         CAAATATTATCTATATAAAAAAATAGATCAACTTTTGGACAATTTGAAAATGCACTTATGTTATTGAATAAGGCGAACGGTGAATATATAAATTTCT
ABLH01000027.1         CAAATATTATCTATATAAAAAAATAGATCAACTTTTGGACAATTTGAAAATGCACTTATGTTATTGAATAAGGCGAACGGTGAATATATAAATTTCT
AAEN01000017.1         CAAATATTATCTATATAAAAAAATAGATCAACTTTTGGACAATTTGAAAATGCACTTATGTTATTGAATAAGGCGAACGGTGAATATATAAATTTCT
NZ_ABKG01000001.1      CAAATATTATCTATATAAAAAAATAGATCAACTTTTGGACAATTTGAAAATGCACTTATGTTATTGAATAAGGCGAACGGTGAATATATAAATTTCT
QAEL01000003.1         CAAATATTATCTATATAAAAAAATAGATCAACTTTTGGACAATTTGAAAATGCACTTATGTTATTGAATAAGGCGAACGGTGAATATATAAATTTCT
NZ_AAEQ01000046.1      CAAATATTATCTATATAAAAAAATAGATCAACTTTTGGACAATTTGAAAATGCACTTATGTTATTGAATAAGGCGAACGGTGAATATATAAATTTCT
AMDT01000002.1         CAAATATTATCTATATAAAAAAATAGATCAACTTTTGGACAATTTGAAAATGCACTTATGTTATTGAATAAGGCGAACGGTGAATATATAAATTTCT
JMPU01000016.1         CAAATATTATCTATATAAAAAAATAGATCAACTTTTGGACAATTTGAAAATGCACTTATGTTATTGAATAAGGCGAACGGTGAATATATAAATTTCT
QAEM01000001.1         CAAATATTATCTATATAAAAAAATAGATCAACTTTTGGACAATTTGAAAATGCACTTATGTTATTGAATAAGGCGAACGGTGAATATATAAATTTCT
QAEN01000002.1         CAAATATTATCTATATAAAAAAATAGATCAACTTTTGGACAATTTGAAAATGCACTTATGTTATTGAATAAGGCGAACGGTGAATATATAAATTTCT
JHDS02000015.1         CAAATATTATCTATATAAAAAAATAGATCAACTTTTGGACAATTTGAAAATGCACTTATGTTATTGAATAAGGCGAACGGTGAATATATAAATTTCT
PVLH01000002.1         CAAATATTATCTATATAAAAAAATAGATCAACTTTTGGACAATTTGAAAATGCACTTATGTTATTGAATAAGGCGAACGGTGAATATATAAATTTCT
MVOA01000067.1         CAAATATTATCTATATAAAAAAATAGATCAACTTTTGGACAATTTGAAAATGCACTTATGTTATTGAATAAGGCGAACGGTGAATATATAAATTTCT
QAEH01000002.1         CAAATATTCTCTATATAAAAAAATAGATCAACTTTTGGACAATTTGAAAATGCACTTATGTTATTGAATAAGGCGAACGGTGAATATATAAATTTCT
RQWM01000005.1         CAAATATTATCTATATAAAAAAATAGATCAACTTTTGGACAATTTGAAAATGCACTTATGTTATTGAATAAGGCGAACGGTGAATATATAAATTTCT
LGCD01000038.1         AAAATATAACATATATAAAAAAATAAATCAACTTTAGGACAATTTGAAAATGCACTTATGTTATTGAATAAGGCAAATGGTGAATATATAAATTTCT
LGCC01000044.1         CAAATATAATCTATATAAAAAAATAGATCAACTTTAGGACAATTTGAAAATGCACTTATGTTATTGAATAAGGCAAATGGTGAATATATAAATTTCT
Consensus              CAAATATTATCTATATAAAAAAATAGATCAACTTTTGGACAATTTGAAAATGCACTTATGTTATTGAATAAGGCGAACGGTGAATATATAAATTTCT
Conservation
```

```
                                  1,260               1,280               1,300               1,320               1,340
NRIV01000001.1(Sterne) TGATGGATGATGACTTATTTCATGTAAATAAAATTCAAAAAAATGATGCAATACTTTTTAAATGATCTAGATAATGAAATTAAGCTTGTAACATCGC
AAEO01000022.1         TGATGGATGATGACTTATTTCATGTAAATAAAATTCAAAAAAATGATGCAATACTTTTTAAATGATCTAGATAATGAAATTAAGCTTGTAACATCGC
ALNY01000049.1         TGATGGATGATGACTTATTTCATGTAAATAAAATTCAAAAAAATGATGCAATACTTTTTAAATGATCTAGATAATGAAATTAAGCTTGTAACATCGC
AGQP01000002.1         TGATGGATGATGACTTATTTCATGTAAATAAAATTCAAAAAAATGATGCAATACTTTTTAAATGATCTAGATAATGAAATTAAGCTTGTAACATCGC
JSZS01000049.1         TGATGGATGATGACTTATTTCATGTAAATAAAATTCAAAAAAATGATGCAATACTTTTTAAATGATCTAGATAATGAAATTAAGCTTGTAACATCGC
JTAG01000063.1         TGATGGATGATGACTTATTTCATGTAAATAAAATTCAAAAAAATGATGCAATACTTTTTAAATGATCTAGATAATGAAATTAAGCTTGTAACATCGC
ABLH01000027.1         TGATGGATGATGACTTATTTCATGTAAATAAAATTCAAAAAAATGATGCAATACTTTTTAAATGATCTAGATAATGAAATTAAGCTTGTAACATCGC
AAEN01000017.1         TGATGGATGATGACTTATTTCATGTAAATAAAATTCAAAAAAATGATGCAATACTTTTTAAATGATCTAGATAATGAAATTAAGCTTGTAACATCGC
NZ_ABKG01000001.1      TGATGGATGATGACTTATTTCATGTAAATAAAATTCAAAAAAATGATGCAATACTTTTTAAATGATCTAGATAATGAAATTAAGCTTGTAACATCGC
QAEL01000003.1         TGATGGATGATGACTTATTTCATGTAAATAAAATTCAAAAAAATGATGCAATACTTTTTAAATGATCTAGATAATGAAATTAAGCTTGTAACATCGC
NZ_AAEQ01000046.1      TGATGGATGATGACTTATTTCATGTAAATAAAATTCAAAAAAATGATGCAATACTTTTTAAATGATCTAGATAATGAAATTAAGCTTGTAACATCGC
AMDT01000002.1         TGATGGATGATGACTTATTTCATGTAAATAAAATTCAAAAAAATGATGCAATACTTTTTAAATGATCTAGATAATGAAATTAAGCTTGTAACATCGC
JMPU01000016.1         TGATGGATGATGACTTATTTCATGTAAATAAAATTCAAAAAAATGATGCAATACTTTTTAAATGATCTAGATAATGAAATTAAGCTTGTAACATCGC
QAEM01000001.1         TGATGGATGATGACTTATTTCATGTAAATAAAATTCAAAAAAATGATGCAATACTTTTTAAATGATCTAGATAATGAAATTAAGCTTGTAACATCGC
QAEN01000002.1         TGATGGATGATGACTTATTTCATGTAAATAAAATTCAAAAAAATGATGCAATACTTTTTAAATGATCTAGATAATGAAATTAAGCTTGTAACATCGC
JHDS02000015.1         TGATGGATGATGACTTATTTCATGTAAATAAAATTCAAAAAAATGATGCAATACTTTTTAAATGATCTAGATAATGAAATTAAGCTTGTAACATCGC
PVLH01000002.1         TGATGGATGATGACTTATTTCATGTAAATAAAATTCAAAAAAATGATGCAATACTTTTTAAATGATCTAGATAATGAAATTAAGCTTGTAACATCGC
MVOA01000067.1         TGATGGATGATGACTTATTTCATGTAAATAAAATTCAAAAAAATGATGCAATACTTTTTAAATGATCTAGATAATGAAATTAAGCTTGTAACATCGC
QAEH01000002.1         TGATGGATGATGACTTATTTCATGTAAATAAAATTCAAAAAAATGATGCAATACTTTTTAAATGATCTAGATAATGAAATTAAGCTTGTAACATCGC
RQWM01000005.1         TGATGGATGATGACTTATTTCATGTAAATAAAATTCAAAAAAATGATGCAATACTTTTTAAATGATCTAGATAATGAAATTAAGCTTGTAACATCGC
LGCD01000038.1         TAATGGATGATGATTTATTTCATGAAAATAAAATTCAAAAAAATGATGAAATACTTTTTAAATGATCTAGATAATGAAATTAAGCTTGTAACATCGC
LGCC01000044.1         TAATGGATGATGATTTATTTCATGTAAATAAAATTCAAAAAAATGATGCAATACTTTTTAAATGATCTAGATAATGAAATAAAGCTTGTAACATCGC
Consensus              TGATGGATGATGACTTATTTCATGTAAATAAAATTCAAAAAAATGATGCAATACTTTTTAAATGATCTAGATAATGAAATTAAGCTTGTAACATCGC
Conservation
```

```
                                      1,360               1,380               1,400               1,420               1,
NRIV01000001.1(Sterne) ATCGTCAAGTAATAGATGGGGAAGGCAACCTATTGCCAGATGTTTATTCAACGATGCCGTTGTTTGATACAGATACAATTATAGAGGGGATACAAT
AAEO01000022.1         ATCGTCAAGTAATAGATGGGGAAGGCAACCTATTGCCAGATGTTTATTCAACGATGCCGTTGTTTGATACAGATACAATTATAGAGGGGATACAAT
ALNY01000049.1         ATCGTCAAGTAATAGATGGGTAAGGCAACCTATTGCCAGATGTTTATTCAACGATGCCGTTGTTTGATACAGATACAATTATAGAGGGGATACAAT
AGQP01000002.1         ATCGTCAAGTAATAGATGGGTAAGGCAACCTATTGCCAGATGTTTATTCAACGATGCCGTTGTTTGATACAGATACAATTATAGAGGGGATACAAT
JSZS01000049.1         ATCGTCAAGTAATAGATGGGGAAGGCAACCTATTGCCAGATGTTTATTCAACGATGCCGTTGTTTGATACAGATACAATTATAGAGGGGATACAAT
JTAG01000063.1         ATCGTCAAGTAATAGATGGGGAAGGCAACCTATTGCCAGATGTTTATTCAACGATGCCGTTGTTTGATACAGATACAATTATAGAGGGGATACAAT
ABLH01000027.1         ATCGTCAAGTAATAGATGGGGAAGGCAACCTATTGCCAGATGTTTATTCAACGATGCCGTTGTTTGATACAGATACAATTATAGAGGGGATACAAT
AAEN01000017.1         ATCGTCAAGTAATAGATGGGGAAGGCAACCTATTGCCAGATGTTTATTCAACGATGCCGTTGTTTGATACAGATACAATTATAGAGGGGATACAAT
NZ_ABKG01000001.1      ATCGTCAAGTAATAGATGGGGAAGGCAACCTATTGCCAGATGTTTATTCAACGATGCCGTTGTTTGATACAGATACAATTATAGAGGGGATACAAT
QAEL01000003.1         ATCGTCAAGTAATAGATGGGGAAGGCAACCTATTGCCAGATGTTTATTCAACGATGCCGTTGTTTGATACAGATACAATTATAGAGGGGATACAAT
NZ_AAEQ01000046.1      ATCGTCAAGTAATAGATGGGGAAGGCAACCTATTGCCAGATGTTTATTCAACGATGCCGTTGTTTGATACAGATACAATTATAGAGGGGATACAAT
AMDT01000002.1         ATCGTCAAGTAATAGATGGGGAAGGCAACCTATTGCCAGATGTTTATTCAACGATGCCGTTGTTTGATACAGATACAATTATAGAGGGGATACAAT
JMPU01000016.1         ATCGTCAAGTAATAGATGGGGAAGGCAACCTATTGCCAGATGTTTATTCAACGATGCCGTTGTTTGATACAGATACAATTATAGAGGGGATACAAT
QAEM01000001.1         ATCGTCAAGTAATAGATGGGGAAGGCAACCTATTGCCAGATGTTTATTCAACGATGCCGTTGTTTGATACAGATACAATTATAGAGGGGATACAAT
QAEN01000002.1         ATCGTCAAGTAATAGATGGGGAAGGCAACCTATTGCCAGATGTTTATTCAACGATGCCGTTGTTTGATACAGATACAATTATAGAGGGGATACAAT
JHDS02000015.1         ATCGTCAAGTAATAGATGGGGAAGGCAACCTATTGCCAGATGTTTATTCAACGATGCCGTTGTTTGATACAGATACAATTATAGAGGGGATACAAT
PVLH01000002.1         ATCGTCAAGTAATAGATGGGGAAGGCAACCTATTGCCAGATGTTTATTCAACGATGCCGTTGTTTGATACAGATACAATTATAGAGGGGATACAAT
MVOA01000067.1         ATCGTCAAGTAATAGATGGGGAAGGCAACCTATTGCCAGATGTTTATTCAACGATGCCGTTGTTTGATACAGATACAATTATAGAGGGGATACAAT
QAEH01000002.1         ATCGTCAAGTAATAGATGGGGAAGGCAACCTATTGCCAGATGTTTATTCAACGATGCCGTTGTTTGATACAGATACAATTATAGAGGGGATACAAT
RQWM01000005.1         ATCGTCAAGTAATAGATGGGGAAGGCAACCTATTGCCAGATGTTTATTCAACGATGCCGTTGTTTGATACAGATACAATTATAGAGGGGATACAAT
LGCD01000038.1         ATCGTCAAGTAATAGATGGGGAAGGCAACCTATTGCCAGATGTTTATTCAACGATGCCGTTGTTTGATACAGATACAATTATAGAGGGGATACAAT
LGCC01000044.1         ATCGTCAAGTAATAGATGGGGAAGGCAACCTATTGCCAGATGTTTATTCAACGATGCCGTTGTTTGATACAGATACAATTATAGAGGGGATACAAT
Consensus              ATCGTCAAGTAATAGATGGGGAAGGCAACCTATTGCCAGATGTTTATTCAACGATGCCGTTGTTTGATACAGATACAATTATAGAGGGGATACAAT
Conservation
```

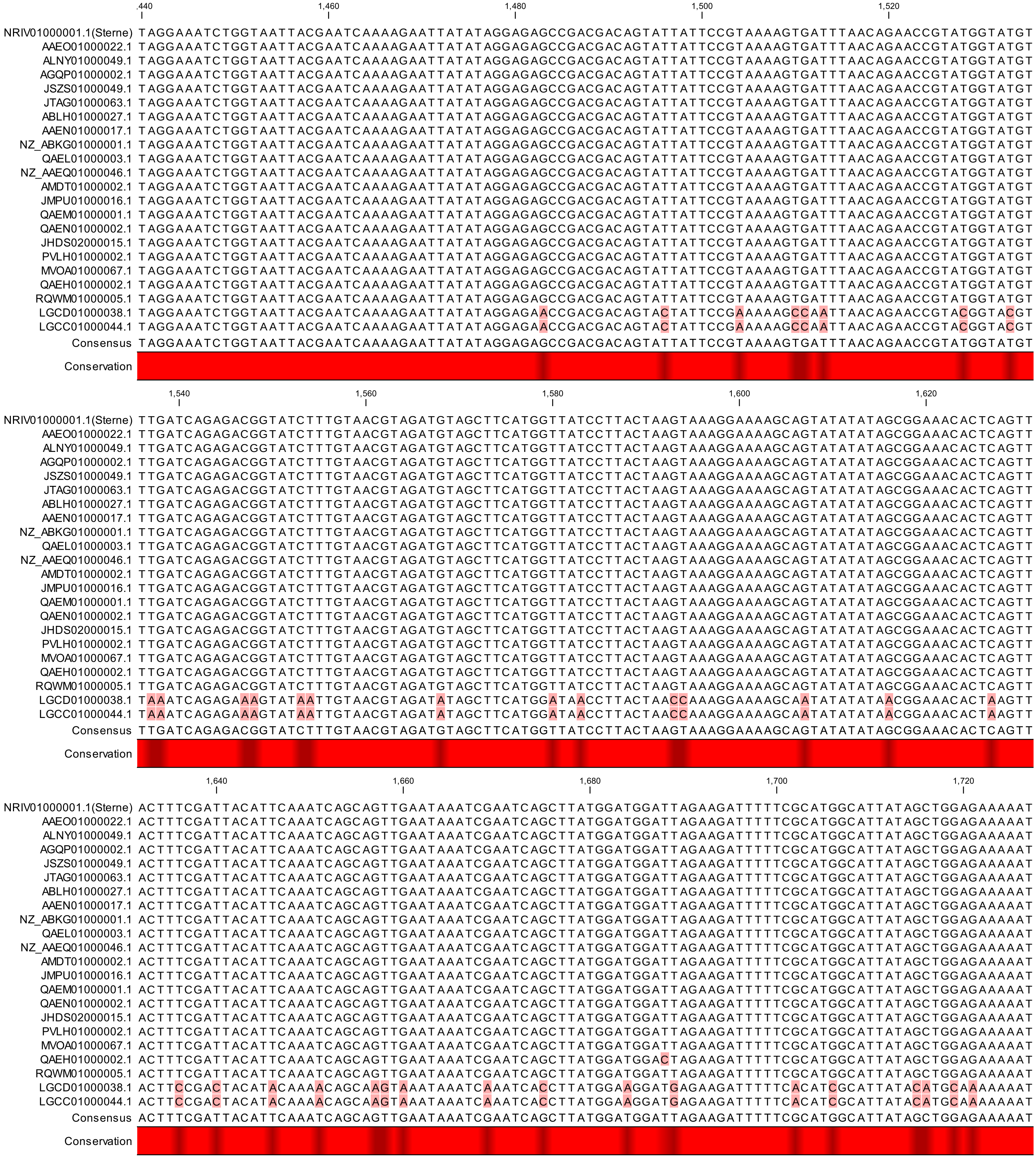

,440 1,460 1,480 1,500 1,520
NRIV01000001.1(Sterne) TAGGAAATCTGGTAATTACGAATCAAAAGAATTATATAGGAGAGCCGACGACAGTATTATTCCGTAAAAGTGATTTAACAGAACCGTATGGTATGT
AAEO01000022.1 TAGGAAATCTGGTAATTACGAATCAAAAGAATTATATAGGAGAGCCGACGACAGTATTATTCCGTAAAAGTGATTTAACAGAACCGTATGGTATGT
ALNY01000049.1 TAGGAAATCTGGTAATTACGAATCAAAAGAATTATATAGGAGAGCCGACGACAGTATTATTCCGTAAAAGTGATTTAACAGAACCGTATGGTATGT
AGQP01000002.1 TAGGAAATCTGGTAATTACGAATCAAAAGAATTATATAGGAGAGCCGACGACAGTATTATTCCGTAAAAGTGATTTAACAGAACCGTATGGTATGT
JSZS01000049.1 TAGGAAATCTGGTAATTACGAATCAAAAGAATTATATAGGAGAGCCGACGACAGTATTATTCCGTAAAAGTGATTTAACAGAACCGTATGGTATGT
JTAG01000063.1 TAGGAAATCTGGTAATTACGAATCAAAAGAATTATATAGGAGAGCCGACGACAGTATTATTCCGTAAAAGTGATTTAACAGAACCGTATGGTATGT
ABLH01000027.1 TAGGAAATCTGGTAATTACGAATCAAAAGAATTATATAGGAGAGCCGACGACAGTATTATTCCGTAAAAGTGATTTAACAGAACCGTATGGTATGT
AAEN01000017.1 TAGGAAATCTGGTAATTACGAATCAAAAGAATTATATAGGAGAGCCGACGACAGTATTATTCCGTAAAAGTGATTTAACAGAACCGTATGGTATGT
NZ_ABKG01000001.1 TAGGAAATCTGGTAATTACGAATCAAAAGAATTATATAGGAGAGCCGACGACAGTATTATTCCGTAAAAGTGATTTAACAGAACCGTATGGTATGT
QAEL01000003.1 TAGGAAATCTGGTAATTACGAATCAAAAGAATTATATAGGAGAGCCGACGACAGTATTATTCCGTAAAAGTGATTTAACAGAACCGTATGGTATGT
NZ_AAEQ01000046.1 TAGGAAATCTGGTAATTACGAATCAAAAGAATTATATAGGAGAGCCGACGACAGTATTATTCCGTAAAAGTGATTTAACAGAACCGTATGGTATGT
AMDT01000002.1 TAGGAAATCTGGTAATTACGAATCAAAAGAATTATATAGGAGAGCCGACGACAGTATTATTCCGTAAAAGTGATTTAACAGAACCGTATGGTATGT
JMPU01000016.1 TAGGAAATCTGGTAATTACGAATCAAAAGAATTATATAGGAGAGCCGACGACAGTATTATTCCGTAAAAGTGATTTAACAGAACCGTATGGTATGT
QAEM01000001.1 TAGGAAATCTGGTAATTACGAATCAAAAGAATTATATAGGAGAGCCGACGACAGTATTATTCCGTAAAAGTGATTTAACAGAACCGTATGGTATGT
QAEN01000002.1 TAGGAAATCTGGTAATTACGAATCAAAAGAATTATATAGGAGAGCCGACGACAGTATTATTCCGTAAAAGTGATTTAACAGAACCGTATGGTATGT
JHDS02000015.1 TAGGAAATCTGGTAATTACGAATCAAAAGAATTATATAGGAGAGCCGACGACAGTATTATTCCGTAAAAGTGATTTAACAGAACCGTATGGTATGT
PVLH01000002.1 TAGGAAATCTGGTAATTACGAATCAAAAGAATTATATAGGAGAGCCGACGACAGTATTATTCCGTAAAAGTGATTTAACAGAACCGTATGGTATGT
MVOA01000067.1 TAGGAAATCTGGTAATTACGAATCAAAAGAATTATATAGGAGAGCCGACGACAGTATTATTCCGTAAAAGTGATTTAACAGAACCGTATGGTATGT
QAEH01000002.1 TAGGAAATCTGGTAATTACGAATCAAAAGAATTATATAGGAGAGCCGACGACAGTATTATTCCGTAAAAGTGATTTAACAGAACCGTATGGTATGT
RQWM01000005.1 TAGGAAATCTGGTAATTACGAATCAAAAGAATTATATAGGAGAGCCGACGACAGTATTATTCCGTAAAAGTGATTTAACAGAACCGTATGGTATGT
LGCD01000038.1 TAGGAAATCTGGTAATTACGAATCAAAAGAATTATATAGGAGAACCGACGACAGTACTATTCCGAAAAAGCCAATTAACAGAACCGTACGGTACGT
LGCC01000044.1 TAGGAAATCTGGTAATTACGAATCAAAAGAATTATATAGGAGAACCGACGACAGTACTATTCCGAAAAAGCCAATTAACAGAACCGTACGGTACGT
Consensus TAGGAAATCTGGTAATTACGAATCAAAAGAATTATATAGGAGAGCCGACGACAGTATTATTCCGTAAAAGTGATTTAACAGAACCGTATGGTATGT
Conservation
1,540 1,560 1,580 1,600 1,620
NRIV01000001.1(Sterne) TTGATCAGAGACGGTATCTTTGTAACGTAGATGTAGCTTCATGGTTATCCTTACTAAGTAAAGGAAAAGCAGTATATATAGCGGAAACACTCAGTT
AAEO01000022.1 TTGATCAGAGACGGTATCTTTGTAACGTAGATGTAGCTTCATGGTTATCCTTACTAAGTAAAGGAAAAGCAGTATATATAGCGGAAACACTCAGTT
ALNY01000049.1 TTGATCAGAGACGGTATCTTTGTAACGTAGATGTAGCTTCATGGTTATCCTTACTAAGTAAAGGAAAAGCAGTATATATAGCGGAAACACTCAGTT
AGQP01000002.1 TTGATCAGAGACGGTATCTTTGTAACGTAGATGTAGCTTCATGGTTATCCTTACTAAGTAAAGGAAAAGCAGTATATATAGCGGAAACACTCAGTT
JSZS01000049.1 TTGATCAGAGACGGTATCTTTGTAACGTAGATGTAGCTTCATGGTTATCCTTACTAAGTAAAGGAAAAGCAGTATATATAGCGGAAACACTCAGTT
JTAG01000063.1 TTGATCAGAGACGGTATCTTTGTAACGTAGATGTAGCTTCATGGTTATCCTTACTAAGTAAAGGAAAAGCAGTATATATAGCGGAAACACTCAGTT
ABLH01000027.1 TTGATCAGAGACGGTATCTTTGTAACGTAGATGTAGCTTCATGGTTATCCTTACTAAGTAAAGGAAAAGCAGTATATATAGCGGAAACACTCAGTT
AAEN01000017.1 TTGATCAGAGACGGTATCTTTGTAACGTAGATGTAGCTTCATGGTTATCCTTACTAAGTAAAGGAAAAGCAGTATATATAGCGGAAACACTCAGTT
NZ_ABKG01000001.1 TTGATCAGAGACGGTATCTTTGTAACGTAGATGTAGCTTCATGGTTATCCTTACTAAGTAAAGGAAAAGCAGTATATATAGCGGAAACACTCAGTT
QAEL01000003.1 TTGATCAGAGACGGTATCTTTGTAACGTAGATGTAGCTTCATGGTTATCCTTACTAAGTAAAGGAAAAGCAGTATATATAGCGGAAACACTCAGTT
NZ_AAEQ01000046.1 TTGATCAGAGACGGTATCTTTGTAACGTAGATGTAGCTTCATGGTTATCCTTACTAAGTAAAGGAAAAGCAGTATATATAGCGGAAACACTCAGTT
AMDT01000002.1 TTGATCAGAGACGGTATCTTTGTAACGTAGATGTAGCTTCATGGTTATCCTTACTAAGTAAAGGAAAAGCAGTATATATAGCGGAAACACTCAGTT
JMPU01000016.1 TTGATCAGAGACGGTATCTTTGTAACGTAGATGTAGCTTCATGGTTATCCTTACTAAGTAAAGGAAAAGCAGTATATATAGCGGAAACACTCAGTT
QAEM01000001.1 TTGATCAGAGACGGTATCTTTGTAACGTAGATGTAGCTTCATGGTTATCCTTACTAAGTAAAGGAAAAGCAGTATATATAGCGGAAACACTCAGTT
QAEN01000002.1 TTGATCAGAGACGGTATCTTTGTAACGTAGATGTAGCTTCATGGTTATCCTTACTAAGTAAAGGAAAAGCAGTATATATAGCGGAAACACTCAGTT
JHDS02000015.1 TTGATCAGAGACGGTATCTTTGTAACGTAGATGTAGCTTCATGGTTATCCTTACTAAGTAAAGGAAAAGCAGTATATATAGCGGAAACACTCAGTT
PVLH01000002.1 TTGATCAGAGACGGTATCTTTGTAACGTAGATGTAGCTTCATGGTTATCCTTACTAAGTAAAGGAAAAGCAGTATATATAGCGGAAACACTCAGTT
MVOA01000067.1 TTGATCAGAGACGGTATCTTTGTAACGTAGATGTAGCTTCATGGTTATCCTTACTAAGTAAAGGAAAAGCAGTATATATAGCGGAAACACTCAGTT
QAEH01000002.1 TTGATCAGAGACGGTATCTTTGTAACGTAGATGTAGCTTCATGGTTATCCTTACTAAGTAAAGGAAAAGCAGTATATATAGCGGAAACACTCAGTT
RQWM01000005.1 TTGATCAGAGACGGTATCTTTGTAACGTAGATGTAGCTTCATGGTTATCCTTACTAAGTAAAGGAAAAGCAGTATATATAGCGGAAACACTCAGTT
LGCD01000038.1 TAAATCAGAGAAAGTATAATTGTAACGTAGATATAGCTTCATGGATAACCTTACTAACCAAAGGAAAAGCAATATATATAACGGAAACACTAAGTT
LGCC01000044.1 TAAATCAGAGAAAGTATAATTGTAACGTAGATATAGCTTCATGGATAACCTTACTAACCAAAGGAAAAGCAATATATATAACGGAAACACTAAGTT
Consensus TTGATCAGAGACGGTATCTTTGTAACGTAGATGTAGCTTCATGGTTATCCTTACTAAGTAAAGGAAAAGCAGTATATATAGCGGAAACACTCAGTT
Conservation
1,640 1,660 1,680 1,700 1,720
NRIV01000001.1(Sterne) ACTTTCGATTACATTCAAATCAGCAGTTGAATAAATCGAATCAGCTTATGGATGGATTAGAAGATTTTTCGCATGGCATTATAGCTGGAGAAAAAT
AAEO01000022.1 ACTTTCGATTACATTCAAATCAGCAGTTGAATAAATCGAATCAGCTTATGGATGGATTAGAAGATTTTTCGCATGGCATTATAGCTGGAGAAAAAT
ALNY01000049.1 ACTTTCGATTACATTCAAATCAGCAGTTGAATAAATCGAATCAGCTTATGGATGGATTAGAAGATTTTTCGCATGGCATTATAGCTGGAGAAAAAT
AGQP01000002.1 ACTTTCGATTACATTCAAATCAGCAGTTGAATAAATCGAATCAGCTTATGGATGGATTAGAAGATTTTTCGCATGGCATTATAGCTGGAGAAAAAT
JSZS01000049.1 ACTTTCGATTACATTCAAATCAGCAGTTGAATAAATCGAATCAGCTTATGGATGGATTAGAAGATTTTTCGCATGGCATTATAGCTGGAGAAAAAT
JTAG01000063.1 ACTTTCGATTACATTCAAATCAGCAGTTGAATAAATCGAATCAGCTTATGGATGGATTAGAAGATTTTTCGCATGGCATTATAGCTGGAGAAAAAT
ABLH01000027.1 ACTTTCGATTACATTCAAATCAGCAGTTGAATAAATCGAATCAGCTTATGGATGGATTAGAAGATTTTTCGCATGGCATTATAGCTGGAGAAAAAT
AAEN01000017.1 ACTTTCGATTACATTCAAATCAGCAGTTGAATAAATCGAATCAGCTTATGGATGGATTAGAAGATTTTTCGCATGGCATTATAGCTGGAGAAAAAT
NZ_ABKG01000001.1 ACTTTCGATTACATTCAAATCAGCAGTTGAATAAATCGAATCAGCTTATGGATGGATTAGAAGATTTTTCGCATGGCATTATAGCTGGAGAAAAAT
QAEL01000003.1 ACTTTCGATTACATTCAAATCAGCAGTTGAATAAATCGAATCAGCTTATGGATGGATTAGAAGATTTTTCGCATGGCATTATAGCTGGAGAAAAAT
NZ_AAEQ01000046.1 ACTTTCGATTACATTCAAATCAGCAGTTGAATAAATCGAATCAGCTTATGGATGGATTAGAAGATTTTTCGCATGGCATTATAGCTGGAGAAAAAT
AMDT01000002.1 ACTTTCGATTACATTCAAATCAGCAGTTGAATAAATCGAATCAGCTTATGGATGGATTAGAAGATTTTTCGCATGGCATTATAGCTGGAGAAAAAT
JMPU01000016.1 ACTTTCGATTACATTCAAATCAGCAGTTGAATAAATCGAATCAGCTTATGGATGGATTAGAAGATTTTTCGCATGGCATTATAGCTGGAGAAAAAT
QAEM01000001.1 ACTTTCGATTACATTCAAATCAGCAGTTGAATAAATCGAATCAGCTTATGGATGGATTAGAAGATTTTTCGCATGGCATTATAGCTGGAGAAAAAT
QAEN01000002.1 ACTTTCGATTACATTCAAATCAGCAGTTGAATAAATCGAATCAGCTTATGGATGGATTAGAAGATTTTTCGCATGGCATTATAGCTGGAGAAAAAT
JHDS02000015.1 ACTTTCGATTACATTCAAATCAGCAGTTGAATAAATCGAATCAGCTTATGGATGGATTAGAAGATTTTTCGCATGGCATTATAGCTGGAGAAAAAT
PVLH01000002.1 ACTTTCGATTACATTCAAATCAGCAGTTGAATAAATCGAATCAGCTTATGGATGGATTAGAAGATTTTTCGCATGGCATTATAGCTGGAGAAAAAT
MVOA01000067.1 ACTTTCGATTACATTCAAATCAGCAGTTGAATAAATCGAATCAGCTTATGGATGGATTAGAAGATTTTTCGCATGGCATTATAGCTGGAGAAAAAT
QAEH01000002.1 ACTTTCGATTACATTCAAATCAGCAGTTGAATAAATCGAATCAGCTTATGGATGGACTAGAAGATTTTTCGCATGGCATTATAGCTGGAGAAAAAT
RQWM01000005.1 ACTTTCGATTACATTCAAATCAGCAGTTGAATAAATCGAATCAGCTTATGGATGGATTAGAAGATTTTTCGCATGGCATTATAGCTGGAGAAAAAT
LGCD01000038.1 ACTTCCGACTACATACAAAACAGCAAGTAAATAAATCAAATCACCTTATGGAAGGATGAGAAGATTTTTCACATCGCATTATACATGCAAAAAAAT
LGCC01000044.1 ACTTCCGACTACATACAAAACAGCAAGTAAATAAATCAAATCACCTTATGGAAGGATGAGAAGATTTTTCACATCGCATTATACATGCAAAAAAAT
Consensus ACTTTCGATTACATTCAAATCAGCAGTTGAATAAATCGAATCAGCTTATGGATGGATTAGAAGATTTTTCGCATGGCATTATAGCTGGAGAAAAAT
Conservation

1,740 1,760 1,780 1,800 1,820

NRIV01000001.1(Sterne)	ATGGTTTCTTATCTTCTGAACAGGAGTTAGATAAAGCTATTACTAATTTTCTAGACTATGCAAGAAGAATCGTTCCATCATCCATACTATATTCAT
AAEO01000022.1	ATGGTTTCTTATCTTCTGAACAGGAGTTAGATAAAGCTATTACTAATTTTCTAGACTATGCAAGAAGAATCGTTCCATCATCCATACTATATTCAT
ALNY01000049.1	ATGGTTTCTTATCTTCTGAACAGGAGTTAGATAAAGCTATTACTAATTTTCTAGACTATGCAAGAAGAATCGTTCCATCATCCATACTATATTCAT
AGQP01000002.1	ATGGTTTCTTATCTTCTGAACAGGAGTTAGATAAAGCTATTACTAATTTTCTAGACTATGCAAGAAGAATCGTTCCATCATCCATACTATATTCAT
JSZS01000049.1	ATGGTTTCTTATCTTCTGAACAGGAGTTAGATAAAGCTATTACTAATTTTCTAGACTATGCAAGAAGAATCGTTCCATCATCCATACTATATTCAT
JTAG01000063.1	ATGGTTTCTTATCTTCTGAACAGGAGTTAGATAAAGCTATTACTAATTTTCTAGACTATGCAAGAAGAATCGTTCCATCATCCATACTATATTCAT
ABLH01000027.1	ATGGTTTCTTATCTTCTGAACAGGAGTTAGATAAAGCTATTACTAATTTTCTAGACTATGCAAGAAGAATCGTTCCATCATCCATACTATATTCAT
AAEN01000017.1	ATGGTTTCTTATCTTCTGAACAGGAGTTAGATAAAGCTATTACTAATTTTCTAGACTATGCAAGAAGAATCGTTCCATCATCCATACTATATTCAT
NZ_ABKG01000001.1	ATGGTTTCTTATCTTCTGAACAGGAGTTAGATAAAGCTATTACTAATTTTCTAGACTATGCAAGAAGAATCGTTCCATCATCCATACTATATTCAT
QAEL01000003.1	ATGGTTTCTTATCTTCTGAACAGGAGTTAGATAAAGCTATTACTAATTTTCTAGACTATGCAAGAAGAATCGTTCCATCATCCATACTATATTCAT
NZ_AAEQ01000046.1	ATGGTTTCTTATCTTCTGAACAGGAGTTAGATAAAGCTATTACTAATTTTCTAGACTATGCAAGAAGAATCGTTCCATCATCCATACTATATTCAT
AMDT01000002.1	ATGGTTTCTTATCTTCTGAACAGGAGTTAGATAAAGCTATTACTAATTTTCTAGACTATGCAAGAAGAATCGTTCCATCATCCATACTATATTCAT
JMPU01000016.1	ATGGTTTCTTATCTTCTGAACAGGAGTTAGATAAAGCTATTACTAATTTTCTAGACTATGCAAGAAGAATCGTTCCATCATCCATACTATATTCAT
QAEM01000001.1	ATGGTTTCTTATCTTCTGAACAGGAGTTAGATAAAGCTATTACTAATTTTCTAGACTATGCAAGAAGAATCGTTCCATCATCCATACTATATTCAT
QAEN01000002.1	ATGGTTTCTTATCTTCTGAACAGGAGTTAGATAAAGCTATTACTAATTTTCTAGACTATGCAAGAAGAATCGTTCCATCATCCATACTATATTCAT
JHDS02000015.1	ATGGTTTCTTATCTTCTGAACAGGAGTTAGATAAAGCTATTACTAATTTTCTAGACTATGCAAGAAGAATCGTTCCATCATCCATACTATATTCAT
PVLH01000002.1	ATGGTTTCTTATCTTCTGAACAGGAGTTAGATAAAGCTATTACTAATTTTCTAGACTATGCAAGAAGAATCGTTCCATCATCCATACTATATTCAT
MVOA01000067.1	ATGGTTTCTTATCTTCTGAACAGGAGTTAGATAAAGCTATTACTAATTTTCTAGACTATGCAAGAAGAATCGTTCCATCATCCATACTATATTCAT
QAEH01000002.1	ATGGTTTCTTATCTTCTGAACAGGAGTTAGATAAAGCTATTACTAATTTTCTAGACTATGCAAGAAGAATCGTTCCATCATCCATACTATATTCAT
RQWM01000005.1	ATGGTTTCTTATCTTCTGAACAGGAGTTAGATAAAGCTATTACTAATTTTCTAGACTATGCAAGAAGAATCGTTCCATCATCCATACTATATTCAT
LGCD01000038.1	ATGGTTTCTTATCTTCTGAACAGGAGTTAGATAAAGCTATTACTAATTTTCTAGACTATGCAAGAAGAATCGTTCCATCATCCATACTATATTCAT
LGCC01000044.1	ATGGTTTCTTATCTTCTGAACAGGAGTTAGATAAAGCTATTACTAATTTTCTAGACTATGCAAGAAGAATCGTTCCATCATCCATACTATATTCAT
Consensus	ATGGTTTCTTATCTTCTGAACAGGAGTTAGATAAAGCTATTACTAATTTTCTAGACTATGCAAGAAGAATCGTTCCATCATCCATACTATATTCAT
Conservation	

1,840 1,860 1,880 1,900 1,

NRIV01000001.1(Sterne)	TGGACTATTATCAACAAATTAAGTTAAAGAAAATAAATTTAGAAAAGAAAAAACAACATATAAATAACAGTTCGCATCTTCCAAAAGTCAGTATAC
AAEO01000022.1	TGGACTATTATCAACAAATTAAGTTAAAGAAAATAAATTTAGAAAAGAAAAAACAACATATAAATAACAGTTCGCATCTTCCAAAAGTCAGTATAC
ALNY01000049.1	TGGACTATTATCAACAAATTAAGTTAAAGAAAATAAATTTAGAAAAGAAAAAACAACATATAAATAACAGTTCGCATCTTCCAAAAGTCAGTATAC
AGQP01000002.1	TGGACTATTATCAACAAATTAAGTTAAAGAAAATAAATTTAGAAAAGAAAAAACAACATATAAATAACAGTTCGCATCTTCCAAAAGTCAGTATAC
JSZS01000049.1	TGGACTATTATCAACAAATTAAGTTAAAGAAAATAAATTTAGAAAAGAAAAAACAACATATAAATAACAGTTCGCATCTTCCAAAAGTCAGTATAC
JTAG01000063.1	TGGACTATTATCAACAAATTAAGTTAAAGAAAATAAATTTAGAAAAGAAAAAACAACATATAAATAACAGTTCGCATCTTCCAAAAGTCAGTATAC
ABLH01000027.1	TGGACTATTATCAACAAATTAAGTTAAAGAAAATAAATTTAGAAAAGAAAAAACAACATATAAATAACAGTTCGCATCTTCCAAAAGTCAGTATAC
AAEN01000017.1	TGGACTATTATCAACAAATTAAGTTAAAGAAAATAAATTTAGAAAAGAAAAAACAACATATAAATAACAGTTCGCATCTTCCAAAAGTCAGTATAC
NZ_ABKG01000001.1	TGGACTATTATCAACAAATTAAGTTAAAGAAAATAAATTTAGAAAAGAAAAAACAACATATAAATAACAGTTCGCATCTTCCAAAAGTCAGTATAC
QAEL01000003.1	TGGACTATTATCAACAAATTAAGTTAAAGAAAATAAATTTAGAAAAGAAAAAACAACATATAAATAACAGTTCGCATCTTCCAAAAGTCAGTATAC
NZ_AAEQ01000046.1	TGGACTATTATCAACAAATTAAGTTAAAGAAAATAAATTTAGAAAAGAAAAAACAACATATAAATAACAGTTCGCATCTTCCAAAAGTCAGTATAC
AMDT01000002.1	TGGACTATTATCAACAAATTAAGTTAAAGAAAATAAATTTAGAAAAGAAAAAACAACATATAAATAACAGTTCGCATCTTCCAAAAGTCAGTATAC
JMPU01000016.1	TGGACTATTATCAACAAATTAAGTTAAAGAAAATAAATTTAGAAAAGAAAAAACAACATATAAATAACAGTTCGCATCTTCCAAAAGTCAGTATAC
QAEM01000001.1	TGGACTATTATCAACAAATTAAGTTAAAGAAAATAAATTTAGAAAAGAAAAAACAACATATAAATAACAGTTCGCATCTTCCAAAAGTCAGTATAC
QAEN01000002.1	TGGACTATTATCAACAAATTAAGTTAAAGAAAATAAATTTAGAAAAGAAAAAACAACATATAAATAACAGTTCGCATCTTCCAAAAGTCAGTATAC
JHDS02000015.1	TGGACTATTATCAACAAATTAAGTTAAAGAAAATAAATTTAGAAAAGAAAAAACAACATATAAATAACAGTTCGCATCTTCCAAAAGTCAGTATAC
PVLH01000002.1	TGGACTATTATCAACAAATTAAGTTAAAGAAAATAAATTTAGAAAAGAAAAAACAACATATAAATAACAGTTCGCATCTTCCAAAAGTCAGTATAC
MVOA01000067.1	TGGACTATTATCAACAAATTAAGTTAAAGAAAATAAATTTAGAAAAGAAAAAACAACATATAAATAACAGTTCGCATCTTCCAAAAGTCAGTATAC
QAEH01000002.1	TGGACTATTATCAACAAATTAAGTTAAAGAAAATAAATTTAGAAAAGAAAAAACAACATATAAATAACAGTTCGCATCTTCCAAAAGTCAGTATAC
RQWM01000005.1	TGGACTATTATCAACAAATTAAGTTAAAGAAAATAAATTTAGAAAAGAAAAAACAACATATAAATAACAGTTCGCATCTTCCAAAAGTCAGTATAC
LGCD01000038.1	TGGACTATTATCAACAAATTAAGTTAAAGAAAACAAAGTTAGAAAAAAAAAAACAACATATAAATAACAGTTCGCATCTTCCAAAAGTAAGTATAC
LGCC01000044.1	TGGACTATTATCAACAAATTAAGTTAAAGAAAACAAAGTTAGAAAAAAAAAAACAACATATAAATAACAGTTCGCATCTTCCAAAAGTAAGTATAC
Consensus	TGGACTATTATCAACAAATTAAGTTAAAGAAAATAAATTTAGAAAAGAAAAAACAACATATAAATAACAGTTCGCATCTTCCAAAAGTCAGTATAC
Conservation	

,920 1,940 1,960 1,980 2,000

NRIV01000001.1(Sterne)	TTATTCCTGCCTACAATAGACCTCATTACTTAGAATTAGCCCTTAAAAGTGCTTTAAACCAAACCTATGAAAATATAGAAATTATTATATCTGACG
AAEO01000022.1	TTATTCCTGCCTACAATAGACCTCATTACTTAGAATTAGCCCTTAAAAGTGCTTTAAACCAAACCTATGAAAATATAGAAATTATTATATCTGACG
ALNY01000049.1	TTATTCCTGCCTACAATAGACCTCATTACTTAGAATTAGCCCTTAAAAGTGCTTTAAACCAAACCTATGAAAATATAGAAATTATTATATCTGACG
AGQP01000002.1	TTATTCCTGCCTACAATAGACCTCATTACTTAGAATTAGCCCTTAAAAGTGCTTTAAACCAAACCTATGAAAATATAGAAATTATTATATCTGACG
JSZS01000049.1	TTATTCCTGCCTACAATAGACCTCATTACTTAGAATTAGCCCTTAAAAGTGCTTTAAACCAAACCTATGAAAATATAGAAATTATTATATCTGACG
JTAG01000063.1	TTATTCCTGCCTACAATAGACCTCATTACTTAGAATTAGCCCTTAAAAGTGCTTTAAACCAAACCTATGAAAATATAGAAATTATTATATCTGACG
ABLH01000027.1	TTATTCCTGCCTACAATAGACCTCATTACTTAGAATTAGCCCTTAAAAGTGCTTTAAACCAAACCTATGAAAATATAGAAATTATTATATCTGACG
AAEN01000017.1	TTATTCCTGCCTACAATAGACCTCATTACTTAGAATTAGCCCTTAAAAGTGCTTTAAACCAAACCTATGAAAATATAGAAATTATTATATCTGACG
NZ_ABKG01000001.1	TTATTCCTGCCTACAATAGACCTCATTACTTAGAATTAGCCCTTAAAAGTGCTTTAAACCAAACCTATGAAAATATAGAAATTATTATATCTGACG
QAEL01000003.1	TTATTCCTGCCTACAATAGACCTCATTACTTAGAATTAGCCCTTAAAAGTGCTTTAAACCAAACCTATGAAAATATAGAAATTATTATATCTGACG
NZ_AAEQ01000046.1	TTATTCCTGCCTACAATAGACCTCATTACTTAGAATTAGCCCTTAAAAGTGCTTTAAACCAAACCTATGAAAATATAGAAATTATTATATCTGACG
AMDT01000002.1	TTATTCCTGCCTACAATAGACCTCATTACTTAGAATTAGCCCTTAAAAGTGCTTTAAACCAAACCTATGAAAATATAGAAATTATTATATCTGACG
JMPU01000016.1	TTATTCCTGCCTACAATAGACCTCATTACTTAGAATTAGCCCTTAAAAGTGCTTTAAACCAAACCTATGAAAATATAGAAATTATTATATCTGACG
QAEM01000001.1	TTATTCCTGCCTACAATAGACCTCATTACTTAGAATTAGCCCTTAAAAGTGCTTTAAACCAAACCTATGAAAATATAGAAATTATTATATCTGACG
QAEN01000002.1	TTATTCCTGCCTACAATAGACCTCATTACTTAGAATTAGCCCTTAAAAGTGCTTTAAACCAAACCTATGAAAATATAGAAATTATTATATCTGACG
JHDS02000015.1	TTATTCCTGCCTACAATAGACCTCATTACTTAGAATTAGCCCTTAAAAGTGCTTTAAACCAAACCTATGAAAATATAGAAATTATTATATCTGACG
PVLH01000002.1	TTATTCCTGCCTACAATAGACCTCATTACTTAGAATTAGCCCTTAAAAGTGCTTTAAACCAAACCTATGAAAATATAGAAATTATTATATCTGACG
MVOA01000067.1	TTATTCCTGCCTACAATAGACCTCATTACTTAGAATTAGCCCTTAAAAGTGCTTTAAACCAAACCTATGAAAATATAGAAATTATTATATCTGACG
QAEH01000002.1	TTATTCCTGCCTACAATAGACCTCATTACTTAGAATTAGCCCTTAAAAGTGCTTTAAACCAAACCTATGAAAATATAGAAATTATTATATCTGACG
RQWM01000005.1	TTATTCCTGCCTACAATAGACCTCATTACTTAGAATTAGCCCTTAAAAGTGCTTTAAACCAAACCTATGAAAATATAGAAATTATTATATCTGACG
LGCD01000038.1	TAATTCCTACCTACAATAGACCGCATTACTTAGAAAAAGCACTAAAAAGTGCGTTAAAACAAACATATCAAAATATAGAAATTATTATAGCGGACG
LGCC01000044.1	TAATTCCTACCTACAATAGACCGCATTACTTAGAAAAAGCACTAAAAAGTGCGTTAAAACAAACATATCAAAATATAGAAATTATTATAGCGGACG
Consensus	TTATTCCTGCCTACAATAGACCTCATTACTTAGAATTAGCCCTTAAAAGTGCTTTAAACCAAACCTATGAAAATATAGAAATTATTATATCTGACG
Conservation	

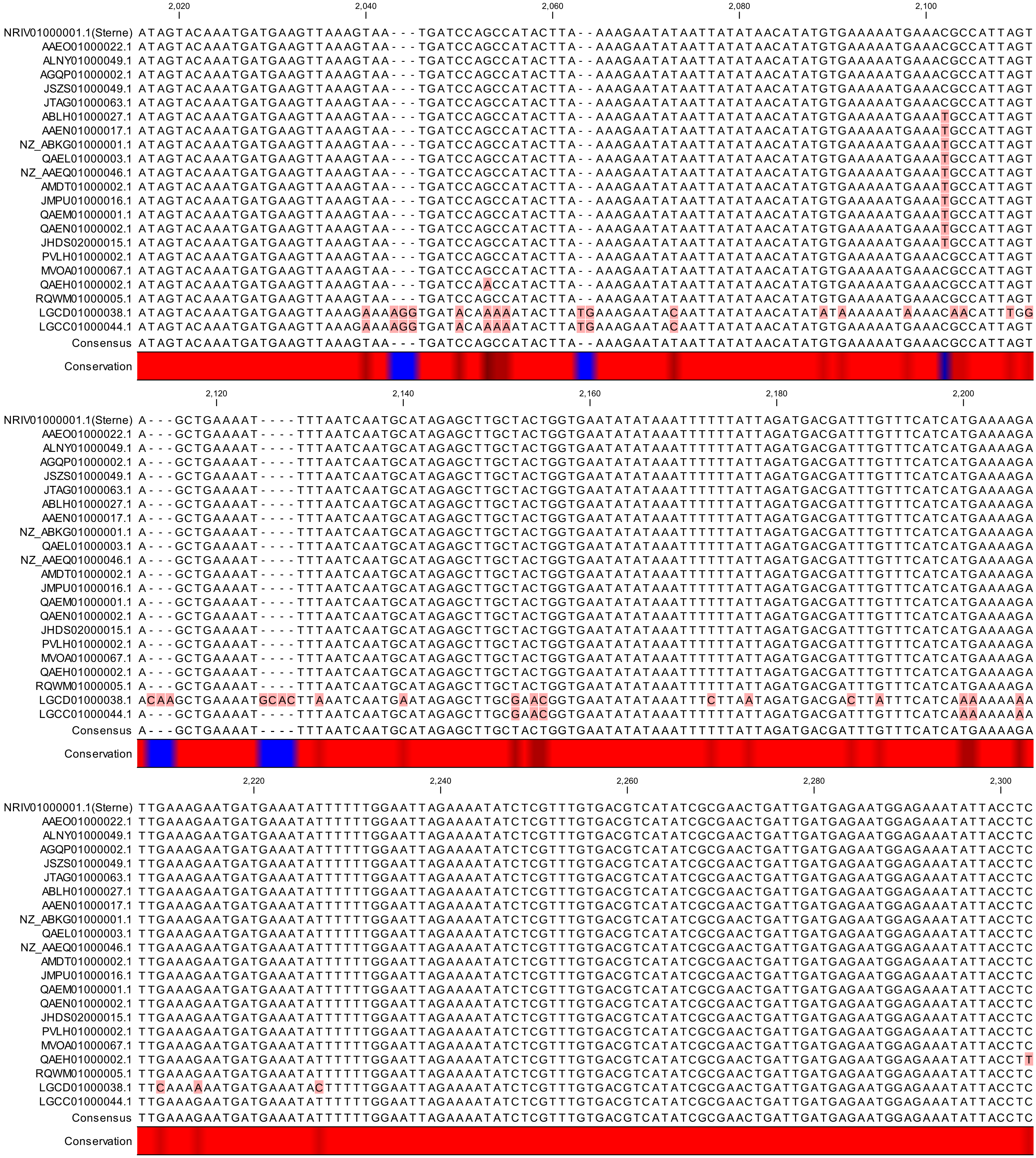

2,020 2,040 2,060 2,080 2,100
NRIV01000001.1(Sterne) ATAGTACAAATGATGAAGTTAAAGTAA---TGATCCAGCCATACTTA--AAAGAATATAATTATATAACATATGTGAAAAATGAAACGCCATTAGT
AAEO01000022.1 ATAGTACAAATGATGAAGTTAAAGTAA---TGATCCAGCCATACTTA--AAAGAATATAATTATATAACATATGTGAAAAATGAAACGCCATTAGT
ALNY01000049.1 ATAGTACAAATGATGAAGTTAAAGTAA---TGATCCAGCCATACTTA--AAAGAATATAATTATATAACATATGTGAAAAATGAAACGCCATTAGT
AGQP01000002.1 ATAGTACAAATGATGAAGTTAAAGTAA---TGATCCAGCCATACTTA--AAAGAATATAATTATATAACATATGTGAAAAATGAAACGCCATTAGT
JSZS01000049.1 ATAGTACAAATGATGAAGTTAAAGTAA---TGATCCAGCCATACTTA--AAAGAATATAATTATATAACATATGTGAAAAATGAAACGCCATTAGT
JTAG01000063.1 ATAGTACAAATGATGAAGTTAAAGTAA---TGATCCAGCCATACTTA--AAAGAATATAATTATATAACATATGTGAAAAATGAAACGCCATTAGT
ABLH01000027.1 ATAGTACAAATGATGAAGTTAAAGTAA---TGATCCAGCCATACTTA--AAAGAATATAATTATATAACATATGTGAAAAATGAAATGCCATTAGT
AAEN01000017.1 ATAGTACAAATGATGAAGTTAAAGTAA---TGATCCAGCCATACTTA--AAAGAATATAATTATATAACATATGTGAAAAATGAAATGCCATTAGT
NZ_ABKG01000001.1 ATAGTACAAATGATGAAGTTAAAGTAA---TGATCCAGCCATACTTA--AAAGAATATAATTATATAACATATGTGAAAAATGAAATGCCATTAGT
QAEL01000003.1 ATAGTACAAATGATGAAGTTAAAGTAA---TGATCCAGCCATACTTA--AAAGAATATAATTATATAACATATGTGAAAAATGAAATGCCATTAGT
NZ_AAEQ01000046.1 ATAGTACAAATGATGAAGTTAAAGTAA---TGATCCAGCCATACTTA--AAAGAATATAATTATATAACATATGTGAAAAATGAAATGCCATTAGT
AMDT01000002.1 ATAGTACAAATGATGAAGTTAAAGTAA---TGATCCAGCCATACTTA--AAAGAATATAATTATATAACATATGTGAAAAATGAAATGCCATTAGT
JMPU01000016.1 ATAGTACAAATGATGAAGTTAAAGTAA---TGATCCAGCCATACTTA--AAAGAATATAATTATATAACATATGTGAAAAATGAAATGCCATTAGT
QAEM01000001.1 ATAGTACAAATGATGAAGTTAAAGTAA---TGATCCAGCCATACTTA--AAAGAATATAATTATATAACATATGTGAAAAATGAAATGCCATTAGT
QAEN01000002.1 ATAGTACAAATGATGAAGTTAAAGTAA---TGATCCAGCCATACTTA--AAAGAATATAATTATATAACATATGTGAAAAATGAAATGCCATTAGT
JHDS02000015.1 ATAGTACAAATGATGAAGTTAAAGTAA---TGATCCAGCCATACTTA--AAAGAATATAATTATATAACATATGTGAAAAATGAAATGCCATTAGT
PVLH01000002.1 ATAGTACAAATGATGAAGTTAAAGTAA---TGATCCAGCCATACTTA--AAAGAATATAATTATATAACATATGTGAAAAATGAAACGCCATTAGT
MVOA01000067.1 ATAGTACAAATGATGAAGTTAAAGTAA---TGATCCAGCCATACTTA--AAAGAATATAATTATATAACATATGTGAAAAATGAAACGCCATTAGT
QAEH01000002.1 ATAGTACAAATGATGAAGTTAAAGTAA---TGATCCAACCATACTTA--AAAGAATATAATTATATAACATATGTGAAAAATGAAACGCCATTAGT
RQWM01000005.1 ATAGTACAAATGATGAAGTTAAAGTAA---TGATCCAGCCATACTTA--AAAGAATATAATTATATAACATATGTGAAAAATGAAACGCCATTAGT
LGCD01000038.1 ATAGTACAAATGATGAAGTTAAAGAAAAGGTGATACAAAAATACTTATGAAAGAATACAATTATATAACATATATAAAAAATAAAACAACATTTGG
LGCC01000044.1 ATAGTACAAATGATGAAGTTAAAGAAAAGGTGATACAAAAATACTTATGAAAGAATACAATTATATAACATATGTGAAAAATGAAACGCCATTAGT
Consensus ATAGTACAAATGATGAAGTTAAAGTAA---TGATCCAGCCATACTTA--AAAGAATATAATTATATAACATATGTGAAAAATGAAACGCCATTAGT
Conservation
2,120 2,140 2,160 2,180 2,200
NRIV01000001.1(Sterne) A---GCTGAAAAT----TTTAATCAATGCATAGAGCTTGCTACTGGTGAATATATAAATTTTTTATTAGATGACGATTTGTTTCATCATGAAAAGA
AAEO01000022.1 A---GCTGAAAAT----TTTAATCAATGCATAGAGCTTGCTACTGGTGAATATATAAATTTTTTATTAGATGACGATTTGTTTCATCATGAAAAGA
ALNY01000049.1 A---GCTGAAAAT----TTTAATCAATGCATAGAGCTTGCTACTGGTGAATATATAAATTTTTTATTAGATGACGATTTGTTTCATCATGAAAAGA
AGQP01000002.1 A---GCTGAAAAT----TTTAATCAATGCATAGAGCTTGCTACTGGTGAATATATAAATTTTTTATTAGATGACGATTTGTTTCATCATGAAAAGA
JSZS01000049.1 A---GCTGAAAAT----TTTAATCAATGCATAGAGCTTGCTACTGGTGAATATATAAATTTTTTATTAGATGACGATTTGTTTCATCATGAAAAGA
JTAG01000063.1 A---GCTGAAAAT----TTTAATCAATGCATAGAGCTTGCTACTGGTGAATATATAAATTTTTTATTAGATGACGATTTGTTTCATCATGAAAAGA
ABLH01000027.1 A---GCTGAAAAT----TTTAATCAATGCATAGAGCTTGCTACTGGTGAATATATAAATTTTTTATTAGATGACGATTTGTTTCATCATGAAAAGA
AAEN01000017.1 A---GCTGAAAAT----TTTAATCAATGCATAGAGCTTGCTACTGGTGAATATATAAATTTTTTATTAGATGACGATTTGTTTCATCATGAAAAGA
NZ_ABKG01000001.1 A---GCTGAAAAT----TTTAATCAATGCATAGAGCTTGCTACTGGTGAATATATAAATTTTTTATTAGATGACGATTTGTTTCATCATGAAAAGA
QAEL01000003.1 A---GCTGAAAAT----TTTAATCAATGCATAGAGCTTGCTACTGGTGAATATATAAATTTTTTATTAGATGACGATTTGTTTCATCATGAAAAGA
NZ_AAEQ01000046.1 A---GCTGAAAAT----TTTAATCAATGCATAGAGCTTGCTACTGGTGAATATATAAATTTTTTATTAGATGACGATTTGTTTCATCATGAAAAGA
AMDT01000002.1 A---GCTGAAAAT----TTTAATCAATGCATAGAGCTTGCTACTGGTGAATATATAAATTTTTTATTAGATGACGATTTGTTTCATCATGAAAAGA
JMPU01000016.1 A---GCTGAAAAT----TTTAATCAATGCATAGAGCTTGCTACTGGTGAATATATAAATTTTTTATTAGATGACGATTTGTTTCATCATGAAAAGA
QAEM01000001.1 A---GCTGAAAAT----TTTAATCAATGCATAGAGCTTGCTACTGGTGAATATATAAATTTTTTATTAGATGACGATTTGTTTCATCATGAAAAGA
QAEN01000002.1 A---GCTGAAAAT----TTTAATCAATGCATAGAGCTTGCTACTGGTGAATATATAAATTTTTTATTAGATGACGATTTGTTTCATCATGAAAAGA
JHDS02000015.1 A---GCTGAAAAT----TTTAATCAATGCATAGAGCTTGCTACTGGTGAATATATAAATTTTTTATTAGATGACGATTTGTTTCATCATGAAAAGA
PVLH01000002.1 A---GCTGAAAAT----TTTAATCAATGCATAGAGCTTGCTACTGGTGAATATATAAATTTTTTATTAGATGACGATTTGTTTCATCATGAAAAGA
MVOA01000067.1 A---GCTGAAAAT----TTTAATCAATGCATAGAGCTTGCTACTGGTGAATATATAAATTTTTTATTAGATGACGATTTGTTTCATCATGAAAAGA
QAEH01000002.1 A---GCTGAAAAT----TTTAATCAATGCATAGAGCTTGCTACTGGTGAATATATAAATTTTTTATTAGATGACGATTTGTTTCATCATGAAAAGA
RQWM01000005.1 A---GCTGAAAAT----TTTAATCAATGCATAGAGCTTGCTACTGGTGAATATATAAATTTTTTATTAGATGACGATTTGTTTCATCATGAAAAGA
LGCD01000038.1 ACAAGCTGAAAATGCACTTAAATCAATGAATAGAGCTTGCGAACGGTGAATATATAAATTTCTTAATAGATGACGACTTATTTCATCAAAAAAAAA
LGCC01000044.1 A---GCTGAAAAT----TTTAATCAATGCATAGAGCTTGCGAACGGTGAATATATAAATTTTTTATTAGATGACGATTTGTTTCATCAAAAAAAAA
Consensus A---GCTGAAAAT----TTTAATCAATGCATAGAGCTTGCTACTGGTGAATATATAAATTTTTTATTAGATGACGATTTGTTTCATCATGAAAAGA
Conservation
2,220 2,240 2,260 2,280 2,300
NRIV01000001.1(Sterne) TTGAAAGAATGATGAAATATTTTTTGGAATTAGAAAATATCTCGTTTGTGACGTCATATCGCGAACTGATTGATGAGAATGGAGAAATATTACCTC
AAEO01000022.1 TTGAAAGAATGATGAAATATTTTTTGGAATTAGAAAATATCTCGTTTGTGACGTCATATCGCGAACTGATTGATGAGAATGGAGAAATATTACCTC
ALNY01000049.1 TTGAAAGAATGATGAAATATTTTTTGGAATTAGAAAATATCTCGTTTGTGACGTCATATCGCGAACTGATTGATGAGAATGGAGAAATATTACCTC
AGQP01000002.1 TTGAAAGAATGATGAAATATTTTTTGGAATTAGAAAATATCTCGTTTGTGACGTCATATCGCGAACTGATTGATGAGAATGGAGAAATATTACCTC
JSZS01000049.1 TTGAAAGAATGATGAAATATTTTTTGGAATTAGAAAATATCTCGTTTGTGACGTCATATCGCGAACTGATTGATGAGAATGGAGAAATATTACCTC
JTAG01000063.1 TTGAAAGAATGATGAAATATTTTTTGGAATTAGAAAATATCTCGTTTGTGACGTCATATCGCGAACTGATTGATGAGAATGGAGAAATATTACCTC
ABLH01000027.1 TTGAAAGAATGATGAAATATTTTTTGGAATTAGAAAATATCTCGTTTGTGACGTCATATCGCGAACTGATTGATGAGAATGGAGAAATATTACCTC
AAEN01000017.1 TTGAAAGAATGATGAAATATTTTTTGGAATTAGAAAATATCTCGTTTGTGACGTCATATCGCGAACTGATTGATGAGAATGGAGAAATATTACCTC
NZ_ABKG01000001.1 TTGAAAGAATGATGAAATATTTTTTGGAATTAGAAAATATCTCGTTTGTGACGTCATATCGCGAACTGATTGATGAGAATGGAGAAATATTACCTC
QAEL01000003.1 TTGAAAGAATGATGAAATATTTTTTGGAATTAGAAAATATCTCGTTTGTGACGTCATATCGCGAACTGATTGATGAGAATGGAGAAATATTACCTC
NZ_AAEQ01000046.1 TTGAAAGAATGATGAAATATTTTTTGGAATTAGAAAATATCTCGTTTGTGACGTCATATCGCGAACTGATTGATGAGAATGGAGAAATATTACCTC
AMDT01000002.1 TTGAAAGAATGATGAAATATTTTTTGGAATTAGAAAATATCTCGTTTGTGACGTCATATCGCGAACTGATTGATGAGAATGGAGAAATATTACCTC
JMPU01000016.1 TTGAAAGAATGATGAAATATTTTTTGGAATTAGAAAATATCTCGTTTGTGACGTCATATCGCGAACTGATTGATGAGAATGGAGAAATATTACCTC
QAEM01000001.1 TTGAAAGAATGATGAAATATTTTTTGGAATTAGAAAATATCTCGTTTGTGACGTCATATCGCGAACTGATTGATGAGAATGGAGAAATATTACCTC
QAEN01000002.1 TTGAAAGAATGATGAAATATTTTTTGGAATTAGAAAATATCTCGTTTGTGACGTCATATCGCGAACTGATTGATGAGAATGGAGAAATATTACCTC
JHDS02000015.1 TTGAAAGAATGATGAAATATTTTTTGGAATTAGAAAATATCTCGTTTGTGACGTCATATCGCGAACTGATTGATGAGAATGGAGAAATATTACCTC
PVLH01000002.1 TTGAAAGAATGATGAAATATTTTTTGGAATTAGAAAATATCTCGTTTGTGACGTCATATCGCGAACTGATTGATGAGAATGGAGAAATATTACCTC
MVOA01000067.1 TTGAAAGAATGATGAAATATTTTTTGGAATTAGAAAATATCTCGTTTGTGACGTCATATCGCGAACTGATTGATGAGAATGGAGAAATATTACCTC
QAEH01000002.1 TTGAAAGAATGATGAAATATTTTTTGGAATTAGAAAATATCTCGTTTGTGACGTCATATCGCGAACTGATTGATGAGAATGGAGAAATATTACCTT
RQWM01000005.1 TTGAAAGAATGATGAAATATTTTTTGGAATTAGAAAATATCTCGTTTGTGACGTCATATCGCGAACTGATTGATGAGAATGGAGAAATATTACCTC
LGCD01000038.1 TTCAAAAAATGATGAAATACTTTTTGGAATTAGAAAATATCTCGTTTGTGACGTCATATCGCGAACTGATTGATGAGAATGGAGAAATATTACCTC
LGCC01000044.1 TTGAAAGAATGATGAAATATTTTTTGGAATTAGAAAATATCTCGTTTGTGACGTCATATCGCGAACTGATTGATGAGAATGGAGAAATATTACCTC
Consensus TTGAAAGAATGATGAAATATTTTTTGGAATTAGAAAATATCTCGTTTGTGACGTCATATCGCGAACTGATTGATGAGAATGGAGAAATATTACCTC
Conservation

	2,320 · 2,340 · 2,360 · 2,380 · 2,
NRIV01000001.1(Sterne)	CCTCAACCTTAAATGTGAAAATTGCCACAGAAACTACAATTTTTGAAGGAGAAGCACTAGGGAATTATATGTTGAAACATTTGAAAAATGTAGTTG
AAEO01000022.1	CCTCAACCTTAAATGTGAAAATTGCCACAGAAACTACAATTTTTGAAGGAGAAGCACTAGGGAATTATATGTTGAAACATTTGAAAAATGTAGTTG
ALNY01000049.1	CCTCAACCTTAAATGTGAAAATTGCCACAGAAACTACAATTTTTGAAGGAGAAGCACTAGGGAATTATATGTTGAAACATTTGAAAAATGTAGTTG
AGQP01000002.1	CCTCAACCTTAAATGTGAAAATTGCCACAGAAACTACAATTTTTGAAGGAGAAGCACTAGGGAATTATATGTTGAAACATTTGAAAAATGTAGTTG
JSZS01000049.1	CCTCAACCTTAAATGTGAAAATTGCCACAGAAACTACAATTTTTGAAGGAGAAGCACTAGGGAATTATATGTTGAAACATTTGAAAAATGTAGTTG
JTAG01000063.1	CCTCAACCTTAAATGTGAAAATTGCCACAGAAACTACAATTTTTGAAGGAGAAGCACTAGGGAATTATATGTTGAAACATTTGAAAAATGTAGTTG
ABLH01000027.1	CCTCAACCTTAAATGTGAAAATTGCCACAGAAACTACAATTTTTGAAGGAGAAGCACTAGGGAATTATATGTTGAAACATTTGAAAAATGTAGTTG
AAEN01000017.1	CCTCAACCTTAAATGTGAAAATTGCCACAGAAACTACAATTTTTGAAGGAGAAGCACTAGGGAATTATATGTTGAAACATTTGAAAAATGTAGTTG
NZ_ABKG01000001.1	CCTCAACCTTAAATGTGAAAATTGCCACAGAAACTACAATTTTTGAAGGAGAAGCACTAGGGAATTATATGTTGAAACATTTGAAAAATGTAGTTG
QAEL01000003.1	CCTCAACCTTAAATGTGAAAATTGCCACAGAAACTACAATTTTTGAAGGAGAAGCACTAGGGAATTATATGTTGAAACATTTGAAAAATGTAGTTG
NZ_AAEQ01000046.1	CCTCAACCTTAAATGTGAAAATTGCCACAGAAACTACAATTTTTGAAGGAGAAGCACTAGGGAATTATATGTTGAAACATTTGAAAAATGTAGTTG
AMDT01000002.1	CCTCAACCTTAAATGTGAAAATTGCCACAGAAACTACAATTTTTGAAGGAGAAGCACTAGGGAATTATATGTTGAAACATTTGAAAAATGTAGTTG
JMPU01000016.1	CCTCAACCTTAAATGTGAAAATTGCCACAGAAACTACAATTTTTGAAGGAGAAGCACTAGGGAATTATATGTTGAAACATTTGAAAAATGTAGTTG
QAEM01000001.1	CCTCAACCTTAAATGTGAAAATTGCCACAGAAACTACAATTTTTGAAGGAGAAGCACTAGGGAATTATATGTTGAAACATTTGAAAAATGTAGTTG
QAEN01000002.1	CCTCAACCTTAAATGTGAAAATTGCCACAGAAACTACAATTTTTGAAGGAGAAGCACTAGGGAATTATATGTTGAAACATTTGAAAAATGTAGTTG
JHDS02000015.1	CCTCAACCTTAAATGTGAAAATTGCCACAGAAACTACAATTTTTGAAGGAGAAGCACTAGGGAATTATATGTTGAAACATTTGAAAAATGTAGTTG
PVLH01000002.1	CCTCAACCTTAAATGTGAAAATTGCCACAGAAACTACAATTTTTGAAGGAGAAGCACTAGGGAATTATATGTTGAAACATTTGAAAAATGTAGTTG
MVOA01000067.1	CCTCAACCTTAAATGTGAAAATTGCCACAGAAACTACAATTTTTGAAGGAGAAGCACTAGGGAATTATATGTTGAAACATTTGAAAAATGTAGTTG
QAEH01000002.1	CCTCAACATTAAATATGAAAATTGCCACAGAAACTACAATTTTTGAAGGAGAAGCACTAGGGAATTATATGTTGAAACATTTGAAAAATGTAGTTG
RQWM01000005.1	CCTCAACCTTAAATGTGAAAATTGCCACAGAAACTACAATTTTTGAAGGAGAAGCACTAGGGAATTATATGTTGAAACATTTGAAAAATGTAGTTG
LGCD01000038.1	CCTCAACCTTAAATGTGAAAATTGCCACAGAAACTACAATTTTTGAAGGAGAAGCACTAGGGAATTATATGTTGAAACATTTGAAAAATGTAGTTG
LGCC01000044.1	CCTCAACCTTAAATGTGAAAATTGCCACAGAAACTACAATTTTTGAAGGAGAAGCACTAGGGAATTATATGTTGAAACATTTGAAAAATGTAGTTG
Consensus	CCTCAACCTTAAATGTGAAAATTGCCACAGAAACTACAATTTTTGAAGGAGAAGCACTAGGGAATTATATGTTGAAACATTTGAAAAATGTAGTTG
Conservation	

	,400 · 2,420 · 2,440 · 2,460 · 2,480
NRIV01000001.1(Sterne)	GAGAACCGACAACGGTTTTGTTTAATCGTAATTTTTTTGGAGGCAAGTTTGGTTATTTTAAGGGAAAAGCGTATTCAGCCATTAATGACATTGCAA
AAEO01000022.1	GAGAACCGACAACGGTTTTGTTTAATCGTAATTTTTTTGGAGGCAAGTTTGGTTATTTTAAGGGAAAAGCGTATTCAGCCATTAATGACATTGCAA
ALNY01000049.1	GAGAACCGACAACGGTTTTGTTTAATCGTAATTTTTTTGGAGGCAAGTTTGGTTATTTTAAGGGAAAAGCGTATTCAGCCATTAATGACATTGCAA
AGQP01000002.1	GAGAACCGACAACGGTTTTGTTTAATCGTAATTTTTTTGGAGGCAAGTTTGGTTATTTTAAGGGAAAAGCGTATTCAGCCATTAATGACATTGCAA
JSZS01000049.1	GAGAACCGACAACGGTTTTGTTTAATCGTAATTTTTTTGGAGGCAAGTTTGGTTATTTTAAGGGAAAAGCGTATTCAGCCATTAATGACATTGCAA
JTAG01000063.1	GAGAACCGACAACGGTTTTGTTTAATCGTAATTTTTTTGGAGGCAAGTTTGGTTATTTTAAGGGAAAAGCGTATTCAGCCATTAATGACATTGCAA
ABLH01000027.1	GAGAACCGACAACGGTTTTGTTTAATCGTAATTTTTTTGGAGGCAAGTTTGGTTATTTTAAGGGAAAAGCGTATTCAGCCATTAATGACATTGCAA
AAEN01000017.1	GAGAACCGACAACGGTTTTGTTTAATCGTAATTTTTTTGGAGGCAAGTTTGGTTATTTTAAGGGAAAAGCGTATTCAGCCATTAATGACATTGCAA
NZ_ABKG01000001.1	GAGAACCGACAACGGTTTTGTTTAATCGTAATTTTTTTGGAGGCAAGTTTGGTTATTTTAAGGGAAAAGCGTATTCAGCCATTAATGACATTGCAA
QAEL01000003.1	GAGAACCGACAACGGTTTTGTTTAATCGTAATTTTTTTGGAGGCAAGTTTGGTTATTTTAAGGGAAAAGCGTATTCAGCCATTAATGACATTGCAA
NZ_AAEQ01000046.1	GAGAACCGACAACGGTTTTGTTTAATCGTAATTTTTTTGGAGGCAAGTTTGGTTATTTTAAGGGAAAAGCGTATTCAGCCATTAATGACATTGCAA
AMDT01000002.1	GAGAACCGACAACGGTTTTGTTTAATCGTAATTTTTTTGGAGGCAAGTTTGGTTATTTTAAGGGAAAAGCGTATTCAGCCATTAATGACATTGCAA
JMPU01000016.1	GAGAACCGACAACGGTTTTGTTTAATCGTAATTTTTTTGGAGGCAAGTTTGGTTATTTTAAGGGAAAAGCGTATTCAGCCATTAATGACATTGCAA
QAEM01000001.1	GAGAACCGACAACGGTTTTGTTTAATCGTAATTTTTTTGGAGGCAAGTTTGGTTATTTTAAGGGAAAAGCGTATTCAGCCATTAATGACATTGCAA
QAEN01000002.1	GAGAACCGACAACGGTTTTGTTTAATCGTAATTTTTTTGGAGGCAAGTTTGGTTATTTTAAGGGAAAAGCGTATTCAGCCATTAATGACATTGCAA
JHDS02000015.1	GAGAACCGACAACGGTTTTGTTTAATCGTAATTTTTTTGGAGGCAAGTTTGGTTATTTTAAGGGAAAAGCGTATTCAGCCATTAATGACATTGCAA
PVLH01000002.1	GAGAACCGACAACGGTTTTGTTTAATCGTAATTTTTTTGGAGGCAAGTTTGGTTATTTTAAGGGAAAAGCGTATTCAGCCATTAATGACATTGCAA
MVOA01000067.1	GAGAACCGACAACGGTTTTGTTTAATCGTAATTTTTTTGGAGGCAAGTTTGGTTATTTTAAGGGAAAAGCGTATTCAGCCATTAATGACATTGCAA
QAEH01000002.1	GAGAACCGACAACGGTTTTGTTTAATCGTAATTTTTTTGGAGGCAAGTTTGGTTATTTTAAGGGAAAAGCGTATTCAGCCATTAATGACATTGCAA
RQWM01000005.1	GAGAACCGACAACGGTTTTGTTTAATCGTAATTTTTTTGGAGGCAAGTTTGGTTATTTTAAGGGAAAAGCGTATTCAGCCATTAATGACATTGCAA
LGCD01000038.1	GAGAACCGACAACGGTTTTGTTTAATCGTAATTTTTTTGGAGGCAAGTTTGGTTATTTTAAGGGAAAAGCGTATTCAGCCATTAATGACATTGCAA
LGCC01000044.1	GAGAACCGACAACGGTTTTGTTTAATCGTAATTTTTTTGGAGGCAAGTTTGGTTATTTTAAGGGAAAAGCGTATTCAGCCATTAATGACATTGCAA
Consensus	GAGAACCGACAACGGTTTTGTTTAATCGTAATTTTTTTGGAGGCAAGTTTGGTTATTTTAAGGGAAAAGCGTATTCAGCCATTAATGACATTGCAA
Conservation	

	2,500 · 2,520 · 2,540 · 2,560 · 2,580
NRIV01000001.1(Sterne)	CTTGGCTAGATATGATGAGGAAAGGGAAAGTAGTATATATACAAGAACCTCTTAGTTACTTTAGACAACATAGTGGGCAAAATCAAAAGCAAATGC
AAEO01000022.1	CTTGGCTAGATATGATGAGGAAAGGGAAAGTAGTATATATACAAGAACCTCTTAGTTACTTTAGACAACATAGTGGGCAAAATCAAAAGCAAATGC
ALNY01000049.1	CTTGGCTAGATATGATGAGGAAAGGGAAAGTAGTATATATACAAGAACCTCTTAGTTACTTTAGACAACATAGTGGGCAAAATCAAAAGCAAATGC
AGQP01000002.1	CTTGGCTAGATATGATGAGGAAAGGGAAAGTAGTATATATACAAGAACCTCTTAGTTACTTTAGACAACATAGTGGGCAAAATCAAAAGCAAATGC
JSZS01000049.1	CTTGGCTAGATATGATGAGGAAAGGGAAAGTAGTATATATACAAGAACCTCTTAGTTACTTTAGACAACATAGTGGGCAAAATCAAAAGCAAATGC
JTAG01000063.1	CTTGGCTAGATATGATGAGGAAAGGGAAAGTAGTATATATACAAGAACCTCTTAGTTACTTTAGACAACATAGTGGGCAAAATCAAAAGCAAATGC
ABLH01000027.1	CTTGGCTAGATATGATGAGGAAAGGGAAAGTAGTATATATACAAGAACCTCTTAGTTACTTTAGACAACATAGTGGGCAAAATCAAAAGCAAATGC
AAEN01000017.1	CTTGGCTAGATATGATGAGGAAAGGGAAAGTAGTATATATACAAGAACCTCTTAGTTACTTTAGACAACATAGTGGGCAAAATCAAAAGCAAATGC
NZ_ABKG01000001.1	CTTGGCTAGATATGATGAGGAAAGGGAAAGTAGTATATATACAAGAACCTCTTAGTTACTTTAGACAACATAGTGGGCAAAATCAAAAGCAAATGC
QAEL01000003.1	CTTGGCTAGATATGATGAGGAAAGGGAAAGTAGTATATATACAAGAACCTCTTAGTTACTTTAGACAACATAGTGGGCAAAATCAAAAGCAAATGC
NZ_AAEQ01000046.1	CTTGGCTAGATATGATGAGGAAAGGGAAAGTAGTATATATACAAGAACCTCTTAGTTACTTTAGACAACATAGTGGGCAAAATCAAAAGCAAATGC
AMDT01000002.1	CTTGGCTAGATATGATGAGGAAAGGGAAAGTAGTATATATACAAGAACCTCTTAGTTACTTTAGACAACATAGTGGGCAAAATCAAAAGCAAATGC
JMPU01000016.1	CTTGGCTAGATATGATGAGGAAAGGGAAAGTAGTATATATACAAGAACCTCTTAGTTACTTTAGACAACATAGTGGGCAAAATCAAAAGCAAATGC
QAEM01000001.1	CTTGGCTAGATATGATGAGGAAAGGGAAAGTAGTATATATACAAGAACCTCTTAGTTACTTTAGACAACATAGTGGGCAAAATCAAAAGCAAATGC
QAEN01000002.1	CTTGGCTAGATATGATGAGGAAAGGGAAAGTAGTATATATACAAGAACCTCTTAGTTACTTTAGACAACATAGTGGGCAAAATCAAAAGCAAATGC
JHDS02000015.1	CTTGGCTAGATATGATGAGGAAAGGGAAAGTAGTATATATACAAGAACCTCTTAGTTACTTTAGACAACATAGTGGGCAAAATCAAAAGCAAATGC
PVLH01000002.1	CTTGGCTAGATATGATGAGGAAAGGGAAAGTAGTATATATACAAGAACCTCTTAGTTACTTTAGACAACATAGTGGGCAAAATCAAAAGCAAATGC
MVOA01000067.1	CTTGGCTAGATATGATGAGGAAAGGGAAAGTAGTATATATACAAGAACCTCTTAGTTACTTTAGACAACATAGTGGGCAAAATCAAAAGCAAATGC
QAEH01000002.1	CTTGGCTAGATATGATGAGGAAAGGGAAAGTAGTATATATACAAGAACCTCTTAGTTACTTTAGACAACATAGTGGGCAAAATCAAAAGCAAATGC
RQWM01000005.1	CTTGGCTAGATATGATGAGGAAAGGGAAAGTAGTATATATACAAGAACCTCTTAGTTACTTTAGACAACATAGTGGGCAAAATCAAAAGCAAATGC
LGCD01000038.1	CTTGGCTAGATATGATGAGGAAAGGGAAAGTAGTATATATACAAGAACCTCTTAGTTACTTTAGACAACATAGTGGGCAAAATCAAAAGCAAATGC
LGCC01000044.1	CTTGGCTAGATATGATGAGGAAAGGGAAAGTAGTATATATACAAGAACCTCTTAGTTACTTTAGACAACATAGTGGGCAAAATCAAAAGCAAATGC
Consensus	CTTGGCTAGATATGATGAGGAAAGGGAAAGTAGTATATATACAAGAACCTCTTAGTTACTTTAGACAACATAGTGGGCAAAATCAAAAGCAAATGC
Conservation	

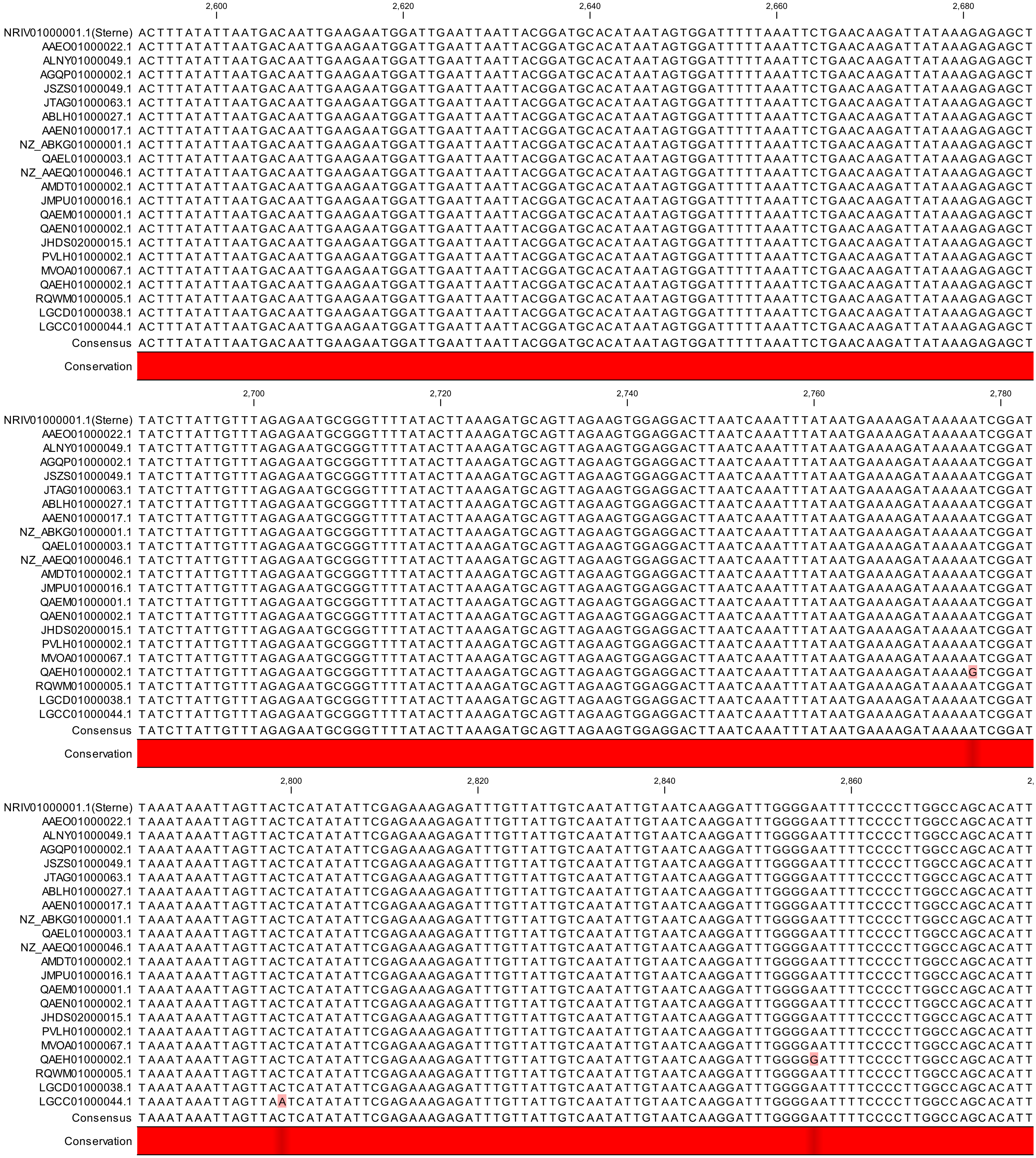
2,600 2,620 2,640 2,660 2,680
NRIV01000001.1(Sterne) ACTTTATATTAATGACAATTGAAGAATGGATTGAATTAATTACGGATGCACATAATAGTGGATTTTTAAATTCTGAACAAGATTATAAAGAGAGCT
AAEO01000022.1 ACTTTATATTAATGACAATTGAAGAATGGATTGAATTAATTACGGATGCACATAATAGTGGATTTTTAAATTCTGAACAAGATTATAAAGAGAGCT
ALNY01000049.1 ACTTTATATTAATGACAATTGAAGAATGGATTGAATTAATTACGGATGCACATAATAGTGGATTTTTAAATTCTGAACAAGATTATAAAGAGAGCT
AGQP01000002.1 ACTTTATATTAATGACAATTGAAGAATGGATTGAATTAATTACGGATGCACATAATAGTGGATTTTTAAATTCTGAACAAGATTATAAAGAGAGCT
JSZS01000049.1 ACTTTATATTAATGACAATTGAAGAATGGATTGAATTAATTACGGATGCACATAATAGTGGATTTTTAAATTCTGAACAAGATTATAAAGAGAGCT
JTAG01000063.1 ACTTTATATTAATGACAATTGAAGAATGGATTGAATTAATTACGGATGCACATAATAGTGGATTTTTAAATTCTGAACAAGATTATAAAGAGAGCT
ABLH01000027.1 ACTTTATATTAATGACAATTGAAGAATGGATTGAATTAATTACGGATGCACATAATAGTGGATTTTTAAATTCTGAACAAGATTATAAAGAGAGCT
AAEN01000017.1 ACTTTATATTAATGACAATTGAAGAATGGATTGAATTAATTACGGATGCACATAATAGTGGATTTTTAAATTCTGAACAAGATTATAAAGAGAGCT
NZ_ABKG01000001.1 ACTTTATATTAATGACAATTGAAGAATGGATTGAATTAATTACGGATGCACATAATAGTGGATTTTTAAATTCTGAACAAGATTATAAAGAGAGCT
QAEL01000003.1 ACTTTATATTAATGACAATTGAAGAATGGATTGAATTAATTACGGATGCACATAATAGTGGATTTTTAAATTCTGAACAAGATTATAAAGAGAGCT
NZ_AAEQ01000046.1 ACTTTATATTAATGACAATTGAAGAATGGATTGAATTAATTACGGATGCACATAATAGTGGATTTTTAAATTCTGAACAAGATTATAAAGAGAGCT
AMDT01000002.1 ACTTTATATTAATGACAATTGAAGAATGGATTGAATTAATTACGGATGCACATAATAGTGGATTTTTAAATTCTGAACAAGATTATAAAGAGAGCT
JMPU01000016.1 ACTTTATATTAATGACAATTGAAGAATGGATTGAATTAATTACGGATGCACATAATAGTGGATTTTTAAATTCTGAACAAGATTATAAAGAGAGCT
QAEM01000001.1 ACTTTATATTAATGACAATTGAAGAATGGATTGAATTAATTACGGATGCACATAATAGTGGATTTTTAAATTCTGAACAAGATTATAAAGAGAGCT
QAEN01000002.1 ACTTTATATTAATGACAATTGAAGAATGGATTGAATTAATTACGGATGCACATAATAGTGGATTTTTAAATTCTGAACAAGATTATAAAGAGAGCT
JHDS02000015.1 ACTTTATATTAATGACAATTGAAGAATGGATTGAATTAATTACGGATGCACATAATAGTGGATTTTTAAATTCTGAACAAGATTATAAAGAGAGCT
PVLH01000002.1 ACTTTATATTAATGACAATTGAAGAATGGATTGAATTAATTACGGATGCACATAATAGTGGATTTTTAAATTCTGAACAAGATTATAAAGAGAGCT
MVOA01000067.1 ACTTTATATTAATGACAATTGAAGAATGGATTGAATTAATTACGGATGCACATAATAGTGGATTTTTAAATTCTGAACAAGATTATAAAGAGAGCT
QAEH01000002.1 ACTTTATATTAATGACAATTGAAGAATGGATTGAATTAATTACGGATGCACATAATAGTGGATTTTTAAATTCTGAACAAGATTATAAAGAGAGCT
RQWM01000005.1 ACTTTATATTAATGACAATTGAAGAATGGATTGAATTAATTACGGATGCACATAATAGTGGATTTTTAAATTCTGAACAAGATTATAAAGAGAGCT
LGCD01000038.1 ACTTTATATTAATGACAATTGAAGAATGGATTGAATTAATTACGGATGCACATAATAGTGGATTTTTAAATTCTGAACAAGATTATAAAGAGAGCT
LGCC01000044.1 ACTTTATATTAATGACAATTGAAGAATGGATTGAATTAATTACGGATGCACATAATAGTGGATTTTTAAATTCTGAACAAGATTATAAAGAGAGCT
Consensus ACTTTATATTAATGACAATTGAAGAATGGATTGAATTAATTACGGATGCACATAATAGTGGATTTTTAAATTCTGAACAAGATTATAAAGAGAGCT
Conservation
2,700 2,720 2,740 2,760 2,780
NRIV01000001.1(Sterne) TATCTTATTGTTTAGAGAATGCGGGTTTTATACTTAAAGATGCAGTTAGAAGTGGAGGACTTAATCAAATTTATAATGAAAAGATAAAAATCGGAT
AAEO01000022.1 TATCTTATTGTTTAGAGAATGCGGGTTTTATACTTAAAGATGCAGTTAGAAGTGGAGGACTTAATCAAATTTATAATGAAAAGATAAAAATCGGAT
ALNY01000049.1 TATCTTATTGTTTAGAGAATGCGGGTTTTATACTTAAAGATGCAGTTAGAAGTGGAGGACTTAATCAAATTTATAATGAAAAGATAAAAATCGGAT
AGQP01000002.1 TATCTTATTGTTTAGAGAATGCGGGTTTTATACTTAAAGATGCAGTTAGAAGTGGAGGACTTAATCAAATTTATAATGAAAAGATAAAAATCGGAT
JSZS01000049.1 TATCTTATTGTTTAGAGAATGCGGGTTTTATACTTAAAGATGCAGTTAGAAGTGGAGGACTTAATCAAATTTATAATGAAAAGATAAAAATCGGAT
JTAG01000063.1 TATCTTATTGTTTAGAGAATGCGGGTTTTATACTTAAAGATGCAGTTAGAAGTGGAGGACTTAATCAAATTTATAATGAAAAGATAAAAATCGGAT
ABLH01000027.1 TATCTTATTGTTTAGAGAATGCGGGTTTTATACTTAAAGATGCAGTTAGAAGTGGAGGACTTAATCAAATTTATAATGAAAAGATAAAAATCGGAT
AAEN01000017.1 TATCTTATTGTTTAGAGAATGCGGGTTTTATACTTAAAGATGCAGTTAGAAGTGGAGGACTTAATCAAATTTATAATGAAAAGATAAAAATCGGAT
NZ_ABKG01000001.1 TATCTTATTGTTTAGAGAATGCGGGTTTTATACTTAAAGATGCAGTTAGAAGTGGAGGACTTAATCAAATTTATAATGAAAAGATAAAAATCGGAT
QAEL01000003.1 TATCTTATTGTTTAGAGAATGCGGGTTTTATACTTAAAGATGCAGTTAGAAGTGGAGGACTTAATCAAATTTATAATGAAAAGATAAAAATCGGAT
NZ_AAEQ01000046.1 TATCTTATTGTTTAGAGAATGCGGGTTTTATACTTAAAGATGCAGTTAGAAGTGGAGGACTTAATCAAATTTATAATGAAAAGATAAAAATCGGAT
AMDT01000002.1 TATCTTATTGTTTAGAGAATGCGGGTTTTATACTTAAAGATGCAGTTAGAAGTGGAGGACTTAATCAAATTTATAATGAAAAGATAAAAATCGGAT
JMPU01000016.1 TATCTTATTGTTTAGAGAATGCGGGTTTTATACTTAAAGATGCAGTTAGAAGTGGAGGACTTAATCAAATTTATAATGAAAAGATAAAAATCGGAT
QAEM01000001.1 TATCTTATTGTTTAGAGAATGCGGGTTTTATACTTAAAGATGCAGTTAGAAGTGGAGGACTTAATCAAATTTATAATGAAAAGATAAAAATCGGAT
QAEN01000002.1 TATCTTATTGTTTAGAGAATGCGGGTTTTATACTTAAAGATGCAGTTAGAAGTGGAGGACTTAATCAAATTTATAATGAAAAGATAAAAATCGGAT
JHDS02000015.1 TATCTTATTGTTTAGAGAATGCGGGTTTTATACTTAAAGATGCAGTTAGAAGTGGAGGACTTAATCAAATTTATAATGAAAAGATAAAAATCGGAT
PVLH01000002.1 TATCTTATTGTTTAGAGAATGCGGGTTTTATACTTAAAGATGCAGTTAGAAGTGGAGGACTTAATCAAATTTATAATGAAAAGATAAAAATCGGAT
MVOA01000067.1 TATCTTATTGTTTAGAGAATGCGGGTTTTATACTTAAAGATGCAGTTAGAAGTGGAGGACTTAATCAAATTTATAATGAAAAGATAAAAATCGGAT
QAEH01000002.1 TATCTTATTGTTTAGAGAATGCGGGTTTTATACTTAAAGATGCAGTTAGAAGTGGAGGACTTAATCAAATTTATAATGAAAAGATAAAAGTCGGAT
RQWM01000005.1 TATCTTATTGTTTAGAGAATGCGGGTTTTATACTTAAAGATGCAGTTAGAAGTGGAGGACTTAATCAAATTTATAATGAAAAGATAAAAATCGGAT
LGCD01000038.1 TATCTTATTGTTTAGAGAATGCGGGTTTTATACTTAAAGATGCAGTTAGAAGTGGAGGACTTAATCAAATTTATAATGAAAAGATAAAAATCGGAT
LGCC01000044.1 TATCTTATTGTTTAGAGAATGCGGGTTTTATACTTAAAGATGCAGTTAGAAGTGGAGGACTTAATCAAATTTATAATGAAAAGATAAAAATCGGAT
Consensus TATCTTATTGTTTAGAGAATGCGGGTTTTATACTTAAAGATGCAGTTAGAAGTGGAGGACTTAATCAAATTTATAATGAAAAGATAAAAATCGGAT
Conservation
2,800 2,820 2,840 2,860 2,
NRIV01000001.1(Sterne) TAAATAAATTAGTTACTCATATATTCGAGAAAGAGATTTGTTATTGTCAATATTGTAATCAAGGATTTGGGGAATTTTCCCCTTGGCCAGCACATT
AAEO01000022.1 TAAATAAATTAGTTACTCATATATTCGAGAAAGAGATTTGTTATTGTCAATATTGTAATCAAGGATTTGGGGAATTTTCCCCTTGGCCAGCACATT
ALNY01000049.1 TAAATAAATTAGTTACTCATATATTCGAGAAAGAGATTTGTTATTGTCAATATTGTAATCAAGGATTTGGGGAATTTTCCCCTTGGCCAGCACATT
AGQP01000002.1 TAAATAAATTAGTTACTCATATATTCGAGAAAGAGATTTGTTATTGTCAATATTGTAATCAAGGATTTGGGGAATTTTCCCCTTGGCCAGCACATT
JSZS01000049.1 TAAATAAATTAGTTACTCATATATTCGAGAAAGAGATTTGTTATTGTCAATATTGTAATCAAGGATTTGGGGAATTTTCCCCTTGGCCAGCACATT
JTAG01000063.1 TAAATAAATTAGTTACTCATATATTCGAGAAAGAGATTTGTTATTGTCAATATTGTAATCAAGGATTTGGGGAATTTTCCCCTTGGCCAGCACATT
ABLH01000027.1 TAAATAAATTAGTTACTCATATATTCGAGAAAGAGATTTGTTATTGTCAATATTGTAATCAAGGATTTGGGGAATTTTCCCCTTGGCCAGCACATT
AAEN01000017.1 TAAATAAATTAGTTACTCATATATTCGAGAAAGAGATTTGTTATTGTCAATATTGTAATCAAGGATTTGGGGAATTTTCCCCTTGGCCAGCACATT
NZ_ABKG01000001.1 TAAATAAATTAGTTACTCATATATTCGAGAAAGAGATTTGTTATTGTCAATATTGTAATCAAGGATTTGGGGAATTTTCCCCTTGGCCAGCACATT
QAEL01000003.1 TAAATAAATTAGTTACTCATATATTCGAGAAAGAGATTTGTTATTGTCAATATTGTAATCAAGGATTTGGGGAATTTTCCCCTTGGCCAGCACATT
NZ_AAEQ01000046.1 TAAATAAATTAGTTACTCATATATTCGAGAAAGAGATTTGTTATTGTCAATATTGTAATCAAGGATTTGGGGAATTTTCCCCTTGGCCAGCACATT
AMDT01000002.1 TAAATAAATTAGTTACTCATATATTCGAGAAAGAGATTTGTTATTGTCAATATTGTAATCAAGGATTTGGGGAATTTTCCCCTTGGCCAGCACATT
JMPU01000016.1 TAAATAAATTAGTTACTCATATATTCGAGAAAGAGATTTGTTATTGTCAATATTGTAATCAAGGATTTGGGGAATTTTCCCCTTGGCCAGCACATT
QAEM01000001.1 TAAATAAATTAGTTACTCATATATTCGAGAAAGAGATTTGTTATTGTCAATATTGTAATCAAGGATTTGGGGAATTTTCCCCTTGGCCAGCACATT
QAEN01000002.1 TAAATAAATTAGTTACTCATATATTCGAGAAAGAGATTTGTTATTGTCAATATTGTAATCAAGGATTTGGGGAATTTTCCCCTTGGCCAGCACATT
JHDS02000015.1 TAAATAAATTAGTTACTCATATATTCGAGAAAGAGATTTGTTATTGTCAATATTGTAATCAAGGATTTGGGGAATTTTCCCCTTGGCCAGCACATT
PVLH01000002.1 TAAATAAATTAGTTACTCATATATTCGAGAAAGAGATTTGTTATTGTCAATATTGTAATCAAGGATTTGGGGAATTTTCCCCTTGGCCAGCACATT
MVOA01000067.1 TAAATAAATTAGTTACTCATATATTCGAGAAAGAGATTTGTTATTGTCAATATTGTAATCAAGGATTTGGGGAATTTTCCCCTTGGCCAGCACATT
QAEH01000002.1 TAAATAAATTAGTTACTCATATATTCGAGAAAGAGATTTGTTATTGTCAATATTGTAATCAAGGATTTGGGGGATTTTCCCCTTGGCCAGCACATT
RQWM01000005.1 TAAATAAATTAGTTACTCATATATTCGAGAAAGAGATTTGTTATTGTCAATATTGTAATCAAGGATTTGGGGAATTTTCCCCTTGGCCAGCACATT
LGCD01000038.1 TAAATAAATTAGTTACTCATATATTCGAGAAAGAGATTTGTTATTGTCAATATTGTAATCAAGGATTTGGGGAATTTTCCCCTTGGCCAGCACATT
LGCC01000044.1 TAAATAAATTAGTTAATCATATATTCGAGAAAGAGATTTGTTATTGTCAATATTGTAATCAAGGATTTGGGGAATTTTCCCCTTGGCCAGCACATT
Consensus TAAATAAATTAGTTACTCATATATTCGAGAAAGAGATTTGTTATTGTCAATATTGTAATCAAGGATTTGGGGAATTTTCCCCTTGGCCAGCACATT
Conservation

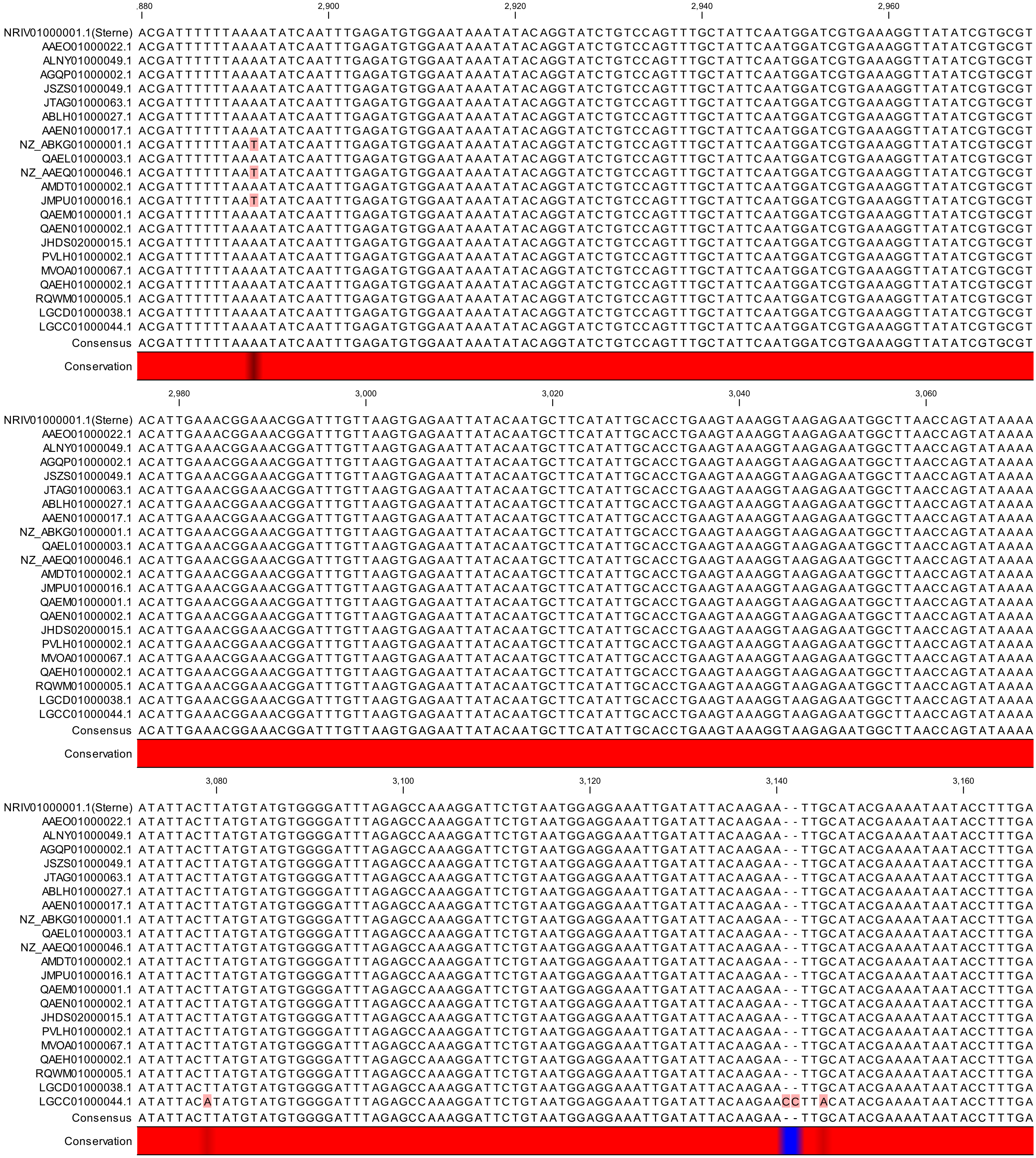
,880
2,900
2,920
2,940
2,960
NRIV01000001.1(Sterne), AAEO01000022.1, ALNY01000049.1, AGQP01000002.1, JSZS01000049.1, JTAG01000063.1, ABLH01000027.1, AAEN01000017.1, QAEL01000003.1, AMDT01000002.1, QAEM01000001.1, QAEN01000002.1, JHDS02000015.1, PVLH01000002.1, MVOA01000067.1, QAEH01000002.1, RQWM01000005.1, LGCD01000038.1, LGCC01000044.1: ACGATTTTTTAAAATATCAATTTGAGATGTGGAATAAATATACAGGTATCTGTCCAGTTTGCTATTCAATGGATCGTGAAAGGTTATATCGTGCGT
NZ_ABKG01000001.1, NZ_AAEQ01000046.1, JMPU01000016.1: ACGATTTTTTAATATATCAATTTGAGATGTGGAATAAATATACAGGTATCTGTCCAGTTTGCTATTCAATGGATCGTGAAAGGTTATATCGTGCGT
Consensus ACGATTTTTTAAAATATCAATTTGAGATGTGGAATAAATATACAGGTATCTGTCCAGTTTGCTATTCAATGGATCGTGAAAGGTTATATCGTGCGT
Conservation
2,980
3,000
3,020
3,040
3,060
All sequences (NRIV01000001.1(Sterne) through LGCC01000044.1): ACATTGAAACGGAAACGGATTTGTTAAGTGAGAATTATACAATGCTTCATATTGCACCTGAAGTAAAGGTAAGAGAATGGCTTAACCAGTATAAAA
Consensus ACATTGAAACGGAAACGGATTTGTTAAGTGAGAATTATACAATGCTTCATATTGCACCTGAAGTAAAGGTAAGAGAATGGCTTAACCAGTATAAAA
Conservation
3,080
3,100
3,120
3,140
3,160
NRIV01000001.1(Sterne) through LGCD01000038.1: ATATTACTTATGTATGTGGGGATTTAGAGCCAAAGGATTCTGTAATGGAGGAAATTGATATTACAAGAA--TTGCATACGAAAATAATACCTTTGA
LGCC01000044.1 ATATTACATATGTATGTGGGGATTTAGAGCCAAAGGATTCTGTAATGGAGGAAATTGATATTACAAGAACCTTACATACGAAAATAATACCTTTGA
Consensus ATATTACTTATGTATGTGGGGATTTAGAGCCAAAGGATTCTGTAATGGAGGAAATTGATATTACAAGAA--TTGCATACGAAAATAATACCTTTGA
Conservation

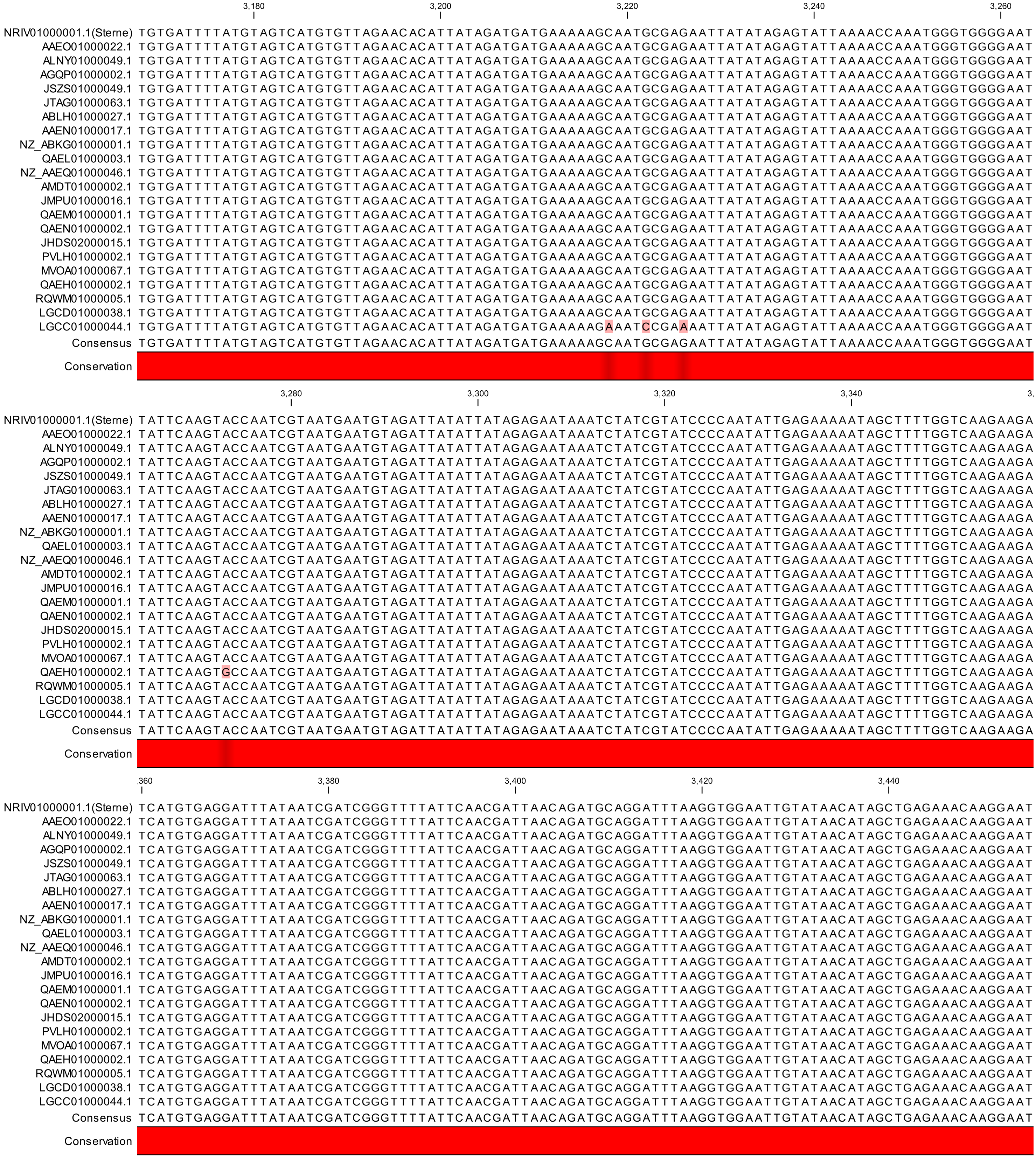
3,180 3,200 3,220 3,240 3,260
NRIV01000001.1(Sterne) TGTGATTTTATGTAGTCATGTGTTAGAACACATTATAGATGATGAAAAAGCAATGCGAGAATTATATAGAGTATTAAAACCAAATGGGTGGGGAAT
AAEO01000022.1 TGTGATTTTATGTAGTCATGTGTTAGAACACATTATAGATGATGAAAAAGCAATGCGAGAATTATATAGAGTATTAAAACCAAATGGGTGGGGAAT
ALNY01000049.1 TGTGATTTTATGTAGTCATGTGTTAGAACACATTATAGATGATGAAAAAGCAATGCGAGAATTATATAGAGTATTAAAACCAAATGGGTGGGGAAT
AGQP01000002.1 TGTGATTTTATGTAGTCATGTGTTAGAACACATTATAGATGATGAAAAAGCAATGCGAGAATTATATAGAGTATTAAAACCAAATGGGTGGGGAAT
JSZS01000049.1 TGTGATTTTATGTAGTCATGTGTTAGAACACATTATAGATGATGAAAAAGCAATGCGAGAATTATATAGAGTATTAAAACCAAATGGGTGGGGAAT
JTAG01000063.1 TGTGATTTTATGTAGTCATGTGTTAGAACACATTATAGATGATGAAAAAGCAATGCGAGAATTATATAGAGTATTAAAACCAAATGGGTGGGGAAT
ABLH01000027.1 TGTGATTTTATGTAGTCATGTGTTAGAACACATTATAGATGATGAAAAAGCAATGCGAGAATTATATAGAGTATTAAAACCAAATGGGTGGGGAAT
AAEN01000017.1 TGTGATTTTATGTAGTCATGTGTTAGAACACATTATAGATGATGAAAAAGCAATGCGAGAATTATATAGAGTATTAAAACCAAATGGGTGGGGAAT
NZ_ABKG01000001.1 TGTGATTTTATGTAGTCATGTGTTAGAACACATTATAGATGATGAAAAAGCAATGCGAGAATTATATAGAGTATTAAAACCAAATGGGTGGGGAAT
QAEL01000003.1 TGTGATTTTATGTAGTCATGTGTTAGAACACATTATAGATGATGAAAAAGCAATGCGAGAATTATATAGAGTATTAAAACCAAATGGGTGGGGAAT
NZ_AAEQ01000046.1 TGTGATTTTATGTAGTCATGTGTTAGAACACATTATAGATGATGAAAAAGCAATGCGAGAATTATATAGAGTATTAAAACCAAATGGGTGGGGAAT
AMDT01000002.1 TGTGATTTTATGTAGTCATGTGTTAGAACACATTATAGATGATGAAAAAGCAATGCGAGAATTATATAGAGTATTAAAACCAAATGGGTGGGGAAT
JMPU01000016.1 TGTGATTTTATGTAGTCATGTGTTAGAACACATTATAGATGATGAAAAAGCAATGCGAGAATTATATAGAGTATTAAAACCAAATGGGTGGGGAAT
QAEM01000001.1 TGTGATTTTATGTAGTCATGTGTTAGAACACATTATAGATGATGAAAAAGCAATGCGAGAATTATATAGAGTATTAAAACCAAATGGGTGGGGAAT
QAEN01000002.1 TGTGATTTTATGTAGTCATGTGTTAGAACACATTATAGATGATGAAAAAGCAATGCGAGAATTATATAGAGTATTAAAACCAAATGGGTGGGGAAT
JHDS02000015.1 TGTGATTTTATGTAGTCATGTGTTAGAACACATTATAGATGATGAAAAAGCAATGCGAGAATTATATAGAGTATTAAAACCAAATGGGTGGGGAAT
PVLH01000002.1 TGTGATTTTATGTAGTCATGTGTTAGAACACATTATAGATGATGAAAAAGCAATGCGAGAATTATATAGAGTATTAAAACCAAATGGGTGGGGAAT
MVOA01000067.1 TGTGATTTTATGTAGTCATGTGTTAGAACACATTATAGATGATGAAAAAGCAATGCGAGAATTATATAGAGTATTAAAACCAAATGGGTGGGGAAT
QAEH01000002.1 TGTGATTTTATGTAGTCATGTGTTAGAACACATTATAGATGATGAAAAAGCAATGCGAGAATTATATAGAGTATTAAAACCAAATGGGTGGGGAAT
RQWM01000005.1 TGTGATTTTATGTAGTCATGTGTTAGAACACATTATAGATGATGAAAAAGCAATGCGAGAATTATATAGAGTATTAAAACCAAATGGGTGGGGAAT
LGCD01000038.1 TGTGATTTTATGTAGTCATGTGTTAGAACACATTATAGATGATGAAAAAGCAATGCGAGAATTATATAGAGTATTAAAACCAAATGGGTGGGGAAT
LGCC01000044.1 TGTGATTTTATGTAGTCATGTGTTAGAACACATTATAGATGATGAAAAAGAAATCCGAAAATTATATAGAGTATTAAAACCAAATGGGTGGGGAAT
Consensus TGTGATTTTATGTAGTCATGTGTTAGAACACATTATAGATGATGAAAAAGCAATGCGAGAATTATATAGAGTATTAAAACCAAATGGGTGGGGAAT
Conservation
3,280 3,300 3,320 3,340 3,
NRIV01000001.1(Sterne) TATTCAAGTACCAATCGTAATGAATGTAGATTATATTATAGAGAATAAATCTATCGTATCCCCAATATTGAGAAAAATAGCTTTTGGTCAAGAAGA
AAEO01000022.1 TATTCAAGTACCAATCGTAATGAATGTAGATTATATTATAGAGAATAAATCTATCGTATCCCCAATATTGAGAAAAATAGCTTTTGGTCAAGAAGA
ALNY01000049.1 TATTCAAGTACCAATCGTAATGAATGTAGATTATATTATAGAGAATAAATCTATCGTATCCCCAATATTGAGAAAAATAGCTTTTGGTCAAGAAGA
AGQP01000002.1 TATTCAAGTACCAATCGTAATGAATGTAGATTATATTATAGAGAATAAATCTATCGTATCCCCAATATTGAGAAAAATAGCTTTTGGTCAAGAAGA
JSZS01000049.1 TATTCAAGTACCAATCGTAATGAATGTAGATTATATTATAGAGAATAAATCTATCGTATCCCCAATATTGAGAAAAATAGCTTTTGGTCAAGAAGA
JTAG01000063.1 TATTCAAGTACCAATCGTAATGAATGTAGATTATATTATAGAGAATAAATCTATCGTATCCCCAATATTGAGAAAAATAGCTTTTGGTCAAGAAGA
ABLH01000027.1 TATTCAAGTACCAATCGTAATGAATGTAGATTATATTATAGAGAATAAATCTATCGTATCCCCAATATTGAGAAAAATAGCTTTTGGTCAAGAAGA
AAEN01000017.1 TATTCAAGTACCAATCGTAATGAATGTAGATTATATTATAGAGAATAAATCTATCGTATCCCCAATATTGAGAAAAATAGCTTTTGGTCAAGAAGA
NZ_ABKG01000001.1 TATTCAAGTACCAATCGTAATGAATGTAGATTATATTATAGAGAATAAATCTATCGTATCCCCAATATTGAGAAAAATAGCTTTTGGTCAAGAAGA
QAEL01000003.1 TATTCAAGTACCAATCGTAATGAATGTAGATTATATTATAGAGAATAAATCTATCGTATCCCCAATATTGAGAAAAATAGCTTTTGGTCAAGAAGA
NZ_AAEQ01000046.1 TATTCAAGTACCAATCGTAATGAATGTAGATTATATTATAGAGAATAAATCTATCGTATCCCCAATATTGAGAAAAATAGCTTTTGGTCAAGAAGA
AMDT01000002.1 TATTCAAGTACCAATCGTAATGAATGTAGATTATATTATAGAGAATAAATCTATCGTATCCCCAATATTGAGAAAAATAGCTTTTGGTCAAGAAGA
JMPU01000016.1 TATTCAAGTACCAATCGTAATGAATGTAGATTATATTATAGAGAATAAATCTATCGTATCCCCAATATTGAGAAAAATAGCTTTTGGTCAAGAAGA
QAEM01000001.1 TATTCAAGTACCAATCGTAATGAATGTAGATTATATTATAGAGAATAAATCTATCGTATCCCCAATATTGAGAAAAATAGCTTTTGGTCAAGAAGA
QAEN01000002.1 TATTCAAGTACCAATCGTAATGAATGTAGATTATATTATAGAGAATAAATCTATCGTATCCCCAATATTGAGAAAAATAGCTTTTGGTCAAGAAGA
JHDS02000015.1 TATTCAAGTACCAATCGTAATGAATGTAGATTATATTATAGAGAATAAATCTATCGTATCCCCAATATTGAGAAAAATAGCTTTTGGTCAAGAAGA
PVLH01000002.1 TATTCAAGTACCAATCGTAATGAATGTAGATTATATTATAGAGAATAAATCTATCGTATCCCCAATATTGAGAAAAATAGCTTTTGGTCAAGAAGA
MVOA01000067.1 TATTCAAGTACCAATCGTAATGAATGTAGATTATATTATAGAGAATAAATCTATCGTATCCCCAATATTGAGAAAAATAGCTTTTGGTCAAGAAGA
QAEH01000002.1 TATTCAAGTGCCAATCGTAATGAATGTAGATTATATTATAGAGAATAAATCTATCGTATCCCCAATATTGAGAAAAATAGCTTTTGGTCAAGAAGA
RQWM01000005.1 TATTCAAGTACCAATCGTAATGAATGTAGATTATATTATAGAGAATAAATCTATCGTATCCCCAATATTGAGAAAAATAGCTTTTGGTCAAGAAGA
LGCD01000038.1 TATTCAAGTACCAATCGTAATGAATGTAGATTATATTATAGAGAATAAATCTATCGTATCCCCAATATTGAGAAAAATAGCTTTTGGTCAAGAAGA
LGCC01000044.1 TATTCAAGTACCAATCGTAATGAATGTAGATTATATTATAGAGAATAAATCTATCGTATCCCCAATATTGAGAAAAATAGCTTTTGGTCAAGAAGA
Consensus TATTCAAGTACCAATCGTAATGAATGTAGATTATATTATAGAGAATAAATCTATCGTATCCCCAATATTGAGAAAAATAGCTTTTGGTCAAGAAGA
Conservation
,360 3,380 3,400 3,420 3,440
NRIV01000001.1(Sterne) TCATGTGAGGATTTATAATCGATCGGGTTTTATTCAACGATTAACAGATGCAGGATTTAAGGTGGAATTGTATAACATAGCTGAGAAACAAGGAAT
AAEO01000022.1 TCATGTGAGGATTTATAATCGATCGGGTTTTATTCAACGATTAACAGATGCAGGATTTAAGGTGGAATTGTATAACATAGCTGAGAAACAAGGAAT
ALNY01000049.1 TCATGTGAGGATTTATAATCGATCGGGTTTTATTCAACGATTAACAGATGCAGGATTTAAGGTGGAATTGTATAACATAGCTGAGAAACAAGGAAT
AGQP01000002.1 TCATGTGAGGATTTATAATCGATCGGGTTTTATTCAACGATTAACAGATGCAGGATTTAAGGTGGAATTGTATAACATAGCTGAGAAACAAGGAAT
JSZS01000049.1 TCATGTGAGGATTTATAATCGATCGGGTTTTATTCAACGATTAACAGATGCAGGATTTAAGGTGGAATTGTATAACATAGCTGAGAAACAAGGAAT
JTAG01000063.1 TCATGTGAGGATTTATAATCGATCGGGTTTTATTCAACGATTAACAGATGCAGGATTTAAGGTGGAATTGTATAACATAGCTGAGAAACAAGGAAT
ABLH01000027.1 TCATGTGAGGATTTATAATCGATCGGGTTTTATTCAACGATTAACAGATGCAGGATTTAAGGTGGAATTGTATAACATAGCTGAGAAACAAGGAAT
AAEN01000017.1 TCATGTGAGGATTTATAATCGATCGGGTTTTATTCAACGATTAACAGATGCAGGATTTAAGGTGGAATTGTATAACATAGCTGAGAAACAAGGAAT
NZ_ABKG01000001.1 TCATGTGAGGATTTATAATCGATCGGGTTTTATTCAACGATTAACAGATGCAGGATTTAAGGTGGAATTGTATAACATAGCTGAGAAACAAGGAAT
QAEL01000003.1 TCATGTGAGGATTTATAATCGATCGGGTTTTATTCAACGATTAACAGATGCAGGATTTAAGGTGGAATTGTATAACATAGCTGAGAAACAAGGAAT
NZ_AAEQ01000046.1 TCATGTGAGGATTTATAATCGATCGGGTTTTATTCAACGATTAACAGATGCAGGATTTAAGGTGGAATTGTATAACATAGCTGAGAAACAAGGAAT
AMDT01000002.1 TCATGTGAGGATTTATAATCGATCGGGTTTTATTCAACGATTAACAGATGCAGGATTTAAGGTGGAATTGTATAACATAGCTGAGAAACAAGGAAT
JMPU01000016.1 TCATGTGAGGATTTATAATCGATCGGGTTTTATTCAACGATTAACAGATGCAGGATTTAAGGTGGAATTGTATAACATAGCTGAGAAACAAGGAAT
QAEM01000001.1 TCATGTGAGGATTTATAATCGATCGGGTTTTATTCAACGATTAACAGATGCAGGATTTAAGGTGGAATTGTATAACATAGCTGAGAAACAAGGAAT
QAEN01000002.1 TCATGTGAGGATTTATAATCGATCGGGTTTTATTCAACGATTAACAGATGCAGGATTTAAGGTGGAATTGTATAACATAGCTGAGAAACAAGGAAT
JHDS02000015.1 TCATGTGAGGATTTATAATCGATCGGGTTTTATTCAACGATTAACAGATGCAGGATTTAAGGTGGAATTGTATAACATAGCTGAGAAACAAGGAAT
PVLH01000002.1 TCATGTGAGGATTTATAATCGATCGGGTTTTATTCAACGATTAACAGATGCAGGATTTAAGGTGGAATTGTATAACATAGCTGAGAAACAAGGAAT
MVOA01000067.1 TCATGTGAGGATTTATAATCGATCGGGTTTTATTCAACGATTAACAGATGCAGGATTTAAGGTGGAATTGTATAACATAGCTGAGAAACAAGGAAT
QAEH01000002.1 TCATGTGAGGATTTATAATCGATCGGGTTTTATTCAACGATTAACAGATGCAGGATTTAAGGTGGAATTGTATAACATAGCTGAGAAACAAGGAAT
RQWM01000005.1 TCATGTGAGGATTTATAATCGATCGGGTTTTATTCAACGATTAACAGATGCAGGATTTAAGGTGGAATTGTATAACATAGCTGAGAAACAAGGAAT
LGCD01000038.1 TCATGTGAGGATTTATAATCGATCGGGTTTTATTCAACGATTAACAGATGCAGGATTTAAGGTGGAATTGTATAACATAGCTGAGAAACAAGGAAT
LGCC01000044.1 TCATGTGAGGATTTATAATCGATCGGGTTTTATTCAACGATTAACAGATGCAGGATTTAAGGTGGAATTGTATAACATAGCTGAGAAACAAGGAAT
Consensus TCATGTGAGGATTTATAATCGATCGGGTTTTATTCAACGATTAACAGATGCAGGATTTAAGGTGGAATTGTATAACATAGCTGAGAAACAAGGAAT
Conservation

```
                          3,460               3,480               3,500               3,520               3,540
NRIV01000001.1(Sterne) GAAAATTGCTAGGAAATTTGGATTATCTAAAACGGATATGCTGTATATTGTCCGAAAATGATAGAAAGGAATAGAAAAAATATGAATAGTATTCCTT
AAEO01000022.1 GAAAATTGCTAGGAAATTTGGATTATCTAAAACGGATATGCTGTATATTGTCCGAAAATGATAGAAAGGAATAGAAAAAATATGAATAGTATTCCTT
ALNY01000049.1 GAAAATTGCTAGGAAATTTGGATTATCTAAAACGGATATGCTGTATATTGTCCGAAAATGATAGAAAGGAATAGAAAAAATATGAATAGTATTCCTT
AGQP01000002.1 GAAAATTGCTAGGAAATTTGGATTATCTAAAACGGATATGCTGTATATTGTCCGAAAATGATAGAAAGGAATAGAAAAAATATGAATAGTATTCCTT
JSZS01000049.1 GAAAATTGCTAGGAAATTTGGATTATCTAAAACGGATATGCTGTATATTGTCCGAAAATGATAGAAAGGAATAGAAAAAATATGAATAGTATTCCTT
JTAG01000063.1 GAAAATTGCTAGGAAATTTGGATTATCTAAAACGGATATGCTGTATATTGTCCGAAAATGATAGAAAGGAATAGAAAAAATATGAATAGTATTCCTT
ABLH01000027.1 GAAAATTGCTAGGAAATTTGGATTATCTAAAACGGATATGCTGTATATTGTCCGAAAATGATAGAAAGGAATAGAAAAAATATGAATAGTATTCCTT
AAEN01000017.1 GAAAATTGCTAGGAAATTTGGATTATCTAAAACGGATATGCTGTATATTGTCCGAAAATGATAGAAAGGAATAGAAAAAATATGAATAGTATTCCTT
NZ_ABKG01000001.1 GAAAATTGCTAGGAAATTTGGATTATCTAAAACGGATATGCTGTATATTGTCCGAAAATGATAGAAAGGAATAGAAAAAATATGAATAGTATTCCTT
QAEL01000003.1 GAAAATTGCTAGGAAATTTGGATTATCTAAAACGGATATGCTGTATATTGTCCGAAAATGATAGAAAGGAATAGAAAAAATATGAATAGTATTCCTT
NZ_AAEQ01000046.1 GAAAATTGCTAGGAAATTTGGATTATCTAAAACGGATATGCTGTATATTGTCCGAAAATGATAGAAAGGAATAGAAAAAATATGAATAGTATTCCTT
AMDT01000002.1 GAAAATTGCTAGGAAATTTGGATTATCTAAAACGGATATGCTGTATATTGTCCGAAAATGATAGAAAGGAATAGAAAAAATATGAATAGTATTCCTT
JMPU01000016.1 GAAAATTGCTAGGAAATTTGGATTATCTAAAACGGATATGCTGTATATTGTCCGAAAATGATAGAAAGGAATAGAAAAAATATGAATAGTATTCCTT
QAEM01000001.1 GAAAATTGCTAGGAAATTTGGATTATCTAAAACGGATATGCTGTATATTGTCCGAAAATGATAGAAAGGAATAGAAAAAATATGAATAGTATTCCTT
QAEN01000002.1 GAAAATTGCTAGGAAATTTGGATTATCTAAAACGGATATGCTGTATATTGTCCGAAAATGATAGAAAGGAATAGAAAAAATATGAATAGTATTCCTT
JHDS02000015.1 GAAAATTGCTAGGAAATTTGGATTATCTAAAACGGATATGCTGTATATTGTCCGAAAATGATAGAAAGGAATAGAAAAAATATGAATAGTATTCCTT
PVLH01000002.1 GAAAATTGCTAGGAAATTTGGATTATCTAAAACGGATATGCTGTATATTGTCCGAAAATGATAGAAAGGAATAGAAAAAATATGAATAGTATTCCTT
MVOA01000067.1 GAAAATTGCTAGGAAATTTGGATTATCTAAAACGGATATGCTGTATATTGTCCGAAAGTGATAGAAAGGAATAGAAAAAATATGAATAGTATTCCTT
QAEH01000002.1 GAAAATTGCTAGGAAATTTGGATTATCTAAAACGGATATGCTGTATATTGTCCGAAAATGATAGAAAGGAATAGAAAAAATATGAATAGTATTCCTT
RQWM01000005.1 GAAAATTGCTAGGAAATTTGGATTATCTAAAACGGATATGCTGTATATTGTCCGAAAATGATAGAAAGGAATAGAAAAAATATGAATAGTATTCCTT
LGCD01000038.1 GAAAATTGCTAGGAAATTTGGATTATCTAAAACGGATATGCTGTATATTGTCCGAAAATGATAGAAAGGAATAGAAAAAATATGAATAGTATTCCTT
LGCC01000044.1 GAAAATTGCTAGGAAATTTGGATTATCTAAAACGGATATGCTGTATATTGTCCGAAAATGATAGAAAGGAATAGAAAAAATATGAATAGTATTCCTT
Consensus GAAAATTGCTAGGAAATTTGGATTATCTAAAACGGATATGCTGTATATTGTCCGAAAATGATAGAAAGGAATAGAAAAAATATGAATAGTATTCCTT
Conservation
```

```
                          3,560               3,580               3,600               3,620               3,640
NRIV01000001.1(Sterne) TTTTACGTGCGTCAATTGTACCTGTGAATGAGTATTTAGGTGAATTGGAGAAAATTGATATATCCCACATATATACGAATTATGGACCTTTAAATC
AAEO01000022.1 TTTTACGTGCGTCAATTGTACCTGTGAATGAGTATTTAGGTGAATTGGAGAAAATGGATATATCCCACATATATACGAATTATGGACCTTTAAATC
ALNY01000049.1 TTTTACGTGCGTCAATTGTACCTGTGAATGAGTATTTAGGTGAATTGGAGAAAATTGATATATCCCACATATATACGAATTATGGACCTTTAAATC
AGQP01000002.1 TTTTACGTGCGTCAATTGTACCTGTGAATGAGTATTTAGGTGAATTGGAGAAAATTGATATATCCCACATATATACGAATTATGGACCTTTAAATC
JSZS01000049.1 TTTTACGTGCGTCAATTGTACCTGTGAATGAGTATTTAGGTGAATTGGAGAAAATGGATATATCCCACATATATACGAATTATGGACCTTTAAATC
JTAG01000063.1 TTTTACGTGCGTCAATTGTACCTGTGAATGAGTATTTAGGTGAATTGGAGAAAATGGATATATCCCACATATATACGAATTATGGACCTTTAAATC
ABLH01000027.1 TTTTACGTGCGTCAATTGTACCTGTGAATGAGTATTTAGGTGAATTGGAGAAAATTGATATATCCCACATATATACGAATTATGGACCTTTAAATC
AAEN01000017.1 TTTTACGTGCGTCAATTGTACCTGTGAATGAGTATTTAGGTGAATTGGAGAAAATTGATATATCCCACATATATACGAATTATGGACCTTTAAATC
NZ_ABKG01000001.1 TTTTACGTGCGTCAATTGTACCTGTGAATGAGTATTTAGGTGAATTGGAGAAAATTGATATATCCCACATATATACGAATTATGGACCTTTAAATC
QAEL01000003.1 TTTTACGTGCGTCAATTGTACCTGTGAATGAGTATTTAGGTGAATTGGAGAAAATTGATATATCCCACATATATACGAATTATGGACCTTTAAATC
NZ_AAEQ01000046.1 TTTTACGTGCGTCAATTGTACCTGTGAATGAGTATTTAGGTGAATTGGAGAAAATTGATATATCCCACATATATACGAATTATGGACCTTTAAATC
AMDT01000002.1 TTTTACGTGCGTCAATTGTACCTGTGAATGAGTATTTAGGTGAATTGGAGAAAATTGATATATCCCACATATATACGAATTATGGACCTTTAAATC
JMPU01000016.1 TTTTACGTGCGTCAATTGTACCTGTGAATGAGTATTTAGGTGAATTGGAGAAAATTGATATATCCCACATATATACGAATTATGGACCTTTAAATC
QAEM01000001.1 TTTTACGTGCGTCAATTGTACCTGTGAATGAGTATTTAGGTGAATTGGAGAAAATTGATATATCCCACATATATACGAATTATGGACCTTTAAATC
QAEN01000002.1 TTTTACGTGCGTCAATTGTACCTGTGAATGAGTATTTAGGTGAATTGGAGAAAATTGATATATCCCACATATATACGAATTATGGACCTTTAAATC
JHDS02000015.1 TTTTACGTGCGTCAATTGTACCTGTGAATGAGTATTTAGGTGAATTGGAGAAAATTGATATATCCCACATATATACGAATTATGGACCTTTAAATC
PVLH01000002.1 TTTTACGTGCGTCAATTGTACCTGTGAATGAGTATTTAGGTGAATTGGAGAAAATTGATATATCCCACATATATACGAATTATGGACCTTTAAATC
MVOA01000067.1 TTTTACGTGCGTCAATTGTACCTGTGAATGAGTATTTAGGTGAATTGGAGAAAATTGATATATCCCACATATATACGAATTATGGACCTTTAAATC
QAEH01000002.1 TTTTACGTGCGTCAATTGTACCTGTGAATGAGTATTTAGGTGAATTGGAGAAAATGGATATATCCCACATATATACGAATTATGGACCTTTAAATC
RQWM01000005.1 TTTTACGTGCGTCAATTGTACCTGTGAATGAGTATTTAGGTGAATTGGAGAAAATTGATATATCCCACATATATACGAATTATGGACCTTTAAATC
LGCD01000038.1 TTTTACGTGCGTCAATTGTACCTGTGAATGAGTATTTAGGTGAATTGGAGAAAATTGATATATCCCACATATATACGAATTATGGACCTTTAAATC
LGCC01000044.1 TTTTACGTGCGTCAATTGTACCTGTGAATGAGTATTTAGGTGAATTGGAGAAAATTGATATATCCCACATATATACGAATTATGGACCTTTAAATC
Consensus TTTTACGTGCGTCAATTGTACCTGTGAATGAGTATTTAGGTGAATTGGAGAAAATTGATATATCCCACATATATACGAATTATGGACCTTTAAATC
Conservation
```

```
                          3,660               3,680               3,700               3,720               3,740
NRIV01000001.1(Sterne) AACACTTTGAAGAAAAAATTATGTCGGAATTTTTCGATAGTAAAGGGGCTGTTACAACAGTAGCAAACGCAACACTGGGGTTAATGGCAGCAATTC
AAEO01000022.1 AACACTTTGAAGAAAAAATTATGTCGGAATTTTTCGATAGTAAAGGGGCTGTTACAACAGTAGCAAACGCAACACTGGGGTTAATGGCAGCAATTC
ALNY01000049.1 AACACTTTGAAGAAAAAATTATGTCGGAATTTTTCGATAGTAAAGGGGCTGTTACAACAGTAGCAAACGCAACACTGGGGTTAATGGCAGCAATTC
AGQP01000002.1 AACACTTTGAAGAAAAAATTATGTCGGAATTTTTCGATAGTAAAGGGGCTGTTACAACAGTAGCAAACGCAACACTGGGGTTAATGGCAGCAATTC
JSZS01000049.1 AACACTTTGAAGAAAAAATTATGTCGGAATTTTTCGATAGTAAAGGGGCTGTTACAACAGTAGCAAACGCAACACTGGGGTTAATGGCAGCAATTC
JTAG01000063.1 AACACTTTGAAGAAAAAATTATGTCGGAATTTTTCGATAGTAAAGGGGCTGTTACAACAGTAGCAAACGCAACACTGGGGTTAATGGCAGCAATTC
ABLH01000027.1 AACACTTTGAAGAAAAAATTATGTCGGAATTTTTCGATAGTAAAGGGGCTGTTACAACAGTAGCAAACGCAACACTGGGGTTAATGGCAGCAATTC
AAEN01000017.1 AACACTTTGAAGAAAAAATTATGTCGGAATTTTTCGATAGTAAAGGGGCTGTTACAACAGTAGCAAACGCAACACTGGGGTTAATGGCAGCAATTC
NZ_ABKG01000001.1 AACACTTTGAAGAAAAAATTATGTCGGAATTTTTCGATAGTAAAGGGGCTGTTACAACAGTAGCAAACGCAACACTGGGGTTAATGGCAGCAATTC
QAEL01000003.1 AACACTTTGAAGAAAAAATTATGTCGGAATTTTTCGATAGTAAAGGGGCTGTTACAACAGTAGCAAACGCAACACTGGGGTTAATGGCAGCAATTC
NZ_AAEQ01000046.1 AACACTTTGAAGAAAAAATTATGTCGGAATTTTTCGATAGTAAAGGGGCTGTTACAACAGTAGCAAACGCAACACTGGGGTTAATGGCAGCAATTC
AMDT01000002.1 AACACTTTGAAGAAAAAATTATGTCGGAATTTTTCGATAGTAAAGGGGCTGTTACAACAGTAGCAAACGCAACACTGGGGTTAATGGCAGCAATTC
JMPU01000016.1 AACACTTTGAAGAAAAAATTATGTCGGAATTTTTCGATAGTAAAGGGGCTGTTACAACAGTAGCAAACGCAACACTGGGGTTAATGGCAGCAATTC
QAEM01000001.1 AACACTTTGAAGAAAAAATTATGTCGGAATTTTTCGATAGTAAAGGGGCTGTTACAACAGTAGCAAACGCAACACTGGGGTTAATGGCAGCAATTC
QAEN01000002.1 AACACTTTGAAGAAAAAATTATGTCGGAATTTTTCGATAGTAAAGGGGCTGTTACAACAGTAGCAAACGCAACACTGGGGTTAATGGCAGCAATTC
JHDS02000015.1 AACACTTTGAAGAAAAAATTATGTCGGAATTTTTCGATAGTAAAGGGGCTGTTACAACAGTAGCAAACGCAACACTGGGGTTAATGGCAGCAATTC
PVLH01000002.1 AACACTTTGAAGAAAAAATTATGTCGGAATTTTTCGATAGTAAAGGGGCTGTTACAACAGTAGCAAACGCAACACTGGGGTTAATGGCAGCAATTC
MVOA01000067.1 AACACTTTGAAGAAAAAATTATGTCGGAATTTTTCGATAGTAAAGGGGCTGTTACAACAGTAGCAAACGCAACACTGGGGTTAATGGCAGCAATTC
QAEH01000002.1 AACACTTTGAAGAAAAAATTATGTCGGAATTTTTCGATAGTAAAGGGGCTGTTACAACAGTAGCAAACGCAACACTGGGGTTAATGGCAGCAATTC
RQWM01000005.1 AACACTTTGAAGAAAAAATTATGTCGGAATTTTTCGATAGTAAAGGGGCTGTTACAACAGTAGCAAACGCAACACTGGGGTTAATGGCAGCAATTC
LGCD01000038.1 AACACTTTGAAGAAAAAATTATGTCCGAATTTTTCGATAGTAAAGGGGCTGTTACAACAGTAGCAAACGCAACACTGGGGTTAATGGCAGCAATTC
LGCC01000044.1 AACACTTTGAAGAAAAAATTATGTCCGAATTTTTCGATAGTAAAGGGGCTGTTACAACAGTAGCAAACGCAACACTGGGGTTAATGGCAGCAATTC
Consensus AACACTTTGAAGAAAAAATTATGTCGGAATTTTTCGATAGTAAAGGGGCTGTTACAACAGTAGCAAACGCAACACTGGGGTTAATGGCAGCAATTC
Conservation
```

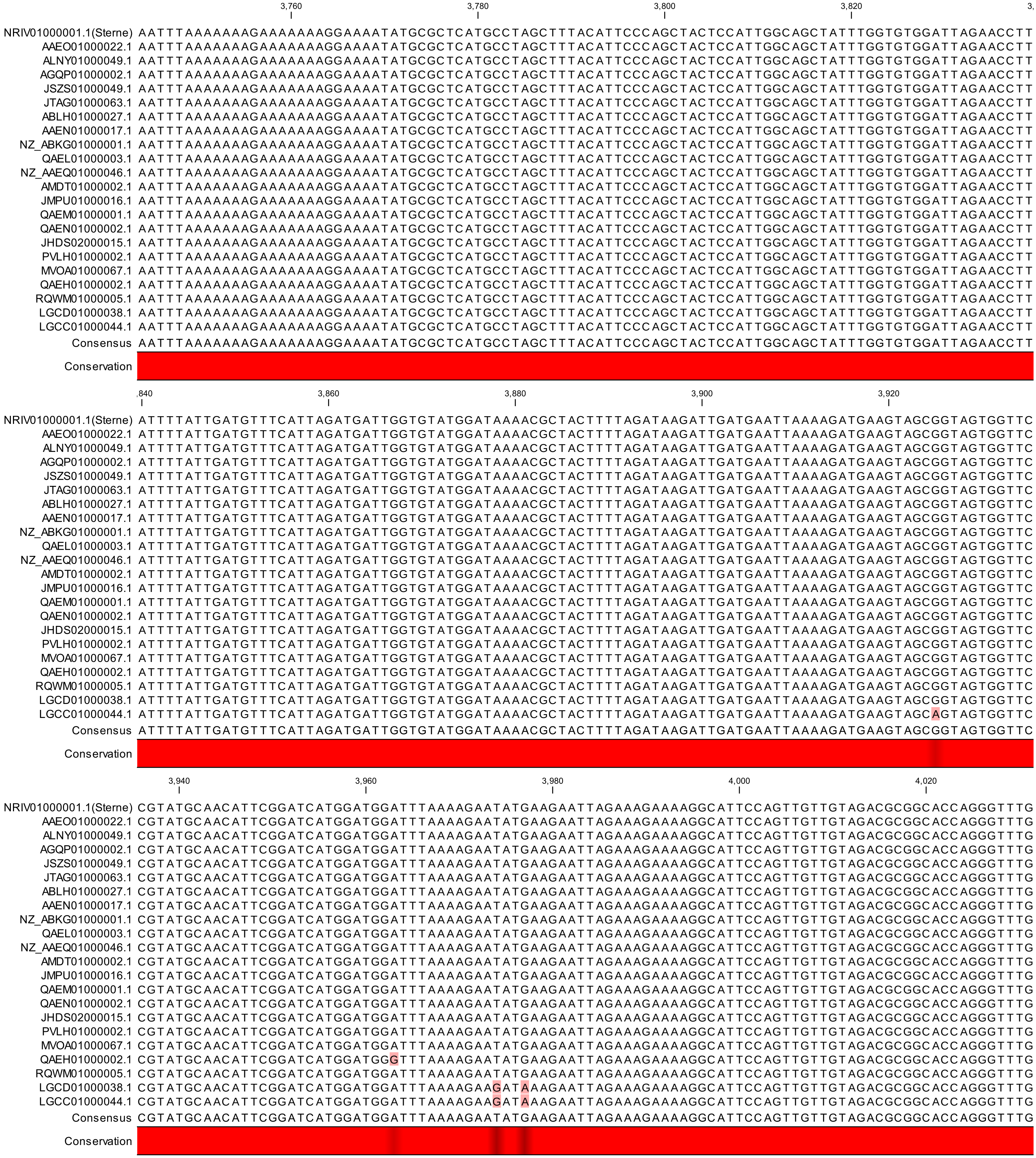

3,760
3,780
3,800
3,820
3,
NRIV01000001.1(Sterne) AATTTAAAAAAAGAAAAAAAAGGAAAATATGCGCTCATGCCTAGCTTTACATTCCCAGCTACTCCATTGGCAGCTATTTGGTGTGGATTAGAACCTT
AAEO01000022.1 AATTTAAAAAAAGAAAAAAAAGGAAAATATGCGCTCATGCCTAGCTTTACATTCCCAGCTACTCCATTGGCAGCTATTTGGTGTGGATTAGAACCTT
ALNY01000049.1 AATTTAAAAAAAGAAAAAAAAGGAAAATATGCGCTCATGCCTAGCTTTACATTCCCAGCTACTCCATTGGCAGCTATTTGGTGTGGATTAGAACCTT
AGQP01000002.1 AATTTAAAAAAAGAAAAAAAAGGAAAATATGCGCTCATGCCTAGCTTTACATTCCCAGCTACTCCATTGGCAGCTATTTGGTGTGGATTAGAACCTT
JSZS01000049.1 AATTTAAAAAAAGAAAAAAAAGGAAAATATGCGCTCATGCCTAGCTTTACATTCCCAGCTACTCCATTGGCAGCTATTTGGTGTGGATTAGAACCTT
JTAG01000063.1 AATTTAAAAAAAGAAAAAAAAGGAAAATATGCGCTCATGCCTAGCTTTACATTCCCAGCTACTCCATTGGCAGCTATTTGGTGTGGATTAGAACCTT
ABLH01000027.1 AATTTAAAAAAAGAAAAAAAAGGAAAATATGCGCTCATGCCTAGCTTTACATTCCCAGCTACTCCATTGGCAGCTATTTGGTGTGGATTAGAACCTT
AAEN01000017.1 AATTTAAAAAAAGAAAAAAAAGGAAAATATGCGCTCATGCCTAGCTTTACATTCCCAGCTACTCCATTGGCAGCTATTTGGTGTGGATTAGAACCTT
NZ_ABKG01000001.1 AATTTAAAAAAAGAAAAAAAAGGAAAATATGCGCTCATGCCTAGCTTTACATTCCCAGCTACTCCATTGGCAGCTATTTGGTGTGGATTAGAACCTT
QAEL01000003.1 AATTTAAAAAAAGAAAAAAAAGGAAAATATGCGCTCATGCCTAGCTTTACATTCCCAGCTACTCCATTGGCAGCTATTTGGTGTGGATTAGAACCTT
NZ_AAEQ01000046.1 AATTTAAAAAAAGAAAAAAAAGGAAAATATGCGCTCATGCCTAGCTTTACATTCCCAGCTACTCCATTGGCAGCTATTTGGTGTGGATTAGAACCTT
AMDT01000002.1 AATTTAAAAAAAGAAAAAAAAGGAAAATATGCGCTCATGCCTAGCTTTACATTCCCAGCTACTCCATTGGCAGCTATTTGGTGTGGATTAGAACCTT
JMPU01000016.1 AATTTAAAAAAAGAAAAAAAAGGAAAATATGCGCTCATGCCTAGCTTTACATTCCCAGCTACTCCATTGGCAGCTATTTGGTGTGGATTAGAACCTT
QAEM01000001.1 AATTTAAAAAAAGAAAAAAAAGGAAAATATGCGCTCATGCCTAGCTTTACATTCCCAGCTACTCCATTGGCAGCTATTTGGTGTGGATTAGAACCTT
QAEN01000002.1 AATTTAAAAAAAGAAAAAAAAGGAAAATATGCGCTCATGCCTAGCTTTACATTCCCAGCTACTCCATTGGCAGCTATTTGGTGTGGATTAGAACCTT
JHDS02000015.1 AATTTAAAAAAAGAAAAAAAAGGAAAATATGCGCTCATGCCTAGCTTTACATTCCCAGCTACTCCATTGGCAGCTATTTGGTGTGGATTAGAACCTT
PVLH01000002.1 AATTTAAAAAAAGAAAAAAAAGGAAAATATGCGCTCATGCCTAGCTTTACATTCCCAGCTACTCCATTGGCAGCTATTTGGTGTGGATTAGAACCTT
MVOA01000067.1 AATTTAAAAAAAGAAAAAAAAGGAAAATATGCGCTCATGCCTAGCTTTACATTCCCAGCTACTCCATTGGCAGCTATTTGGTGTGGATTAGAACCTT
QAEH01000002.1 AATTTAAAAAAAGAAAAAAAAGGAAAATATGCGCTCATGCCTAGCTTTACATTCCCAGCTACTCCATTGGCAGCTATTTGGTGTGGATTAGAACCTT
RQWM01000005.1 AATTTAAAAAAAGAAAAAAAAGGAAAATATGCGCTCATGCCTAGCTTTACATTCCCAGCTACTCCATTGGCAGCTATTTGGTGTGGATTAGAACCTT
LGCD01000038.1 AATTTAAAAAAAGAAAAAAAAGGAAAATATGCGCTCATGCCTAGCTTTACATTCCCAGCTACTCCATTGGCAGCTATTTGGTGTGGATTAGAACCTT
LGCC01000044.1 AATTTAAAAAAAGAAAAAAAAGGAAAATATGCGCTCATGCCTAGCTTTACATTCCCAGCTACTCCATTGGCAGCTATTTGGTGTGGATTAGAACCTT
Consensus AATTTAAAAAAAGAAAAAAAAGGAAAATATGCGCTCATGCCTAGCTTTACATTCCCAGCTACTCCATTGGCAGCTATTTGGTGTGGATTAGAACCTT
Conservation
,840
3,860
3,880
3,900
3,920
NRIV01000001.1(Sterne) ATTTTATTGATGTTTCATTAGATGATTGGTGTATGGATAAAACGCTACTTTTAGATAAGATTGATGAATTAAAAGATGAAGTAGCGGTAGTGGTTC
AAEO01000022.1 ATTTTATTGATGTTTCATTAGATGATTGGTGTATGGATAAAACGCTACTTTTAGATAAGATTGATGAATTAAAAGATGAAGTAGCGGTAGTGGTTC
ALNY01000049.1 ATTTTATTGATGTTTCATTAGATGATTGGTGTATGGATAAAACGCTACTTTTAGATAAGATTGATGAATTAAAAGATGAAGTAGCGGTAGTGGTTC
AGQP01000002.1 ATTTTATTGATGTTTCATTAGATGATTGGTGTATGGATAAAACGCTACTTTTAGATAAGATTGATGAATTAAAAGATGAAGTAGCGGTAGTGGTTC
JSZS01000049.1 ATTTTATTGATGTTTCATTAGATGATTGGTGTATGGATAAAACGCTACTTTTAGATAAGATTGATGAATTAAAAGATGAAGTAGCGGTAGTGGTTC
JTAG01000063.1 ATTTTATTGATGTTTCATTAGATGATTGGTGTATGGATAAAACGCTACTTTTAGATAAGATTGATGAATTAAAAGATGAAGTAGCGGTAGTGGTTC
ABLH01000027.1 ATTTTATTGATGTTTCATTAGATGATTGGTGTATGGATAAAACGCTACTTTTAGATAAGATTGATGAATTAAAAGATGAAGTAGCGGTAGTGGTTC
AAEN01000017.1 ATTTTATTGATGTTTCATTAGATGATTGGTGTATGGATAAAACGCTACTTTTAGATAAGATTGATGAATTAAAAGATGAAGTAGCGGTAGTGGTTC
NZ_ABKG01000001.1 ATTTTATTGATGTTTCATTAGATGATTGGTGTATGGATAAAACGCTACTTTTAGATAAGATTGATGAATTAAAAGATGAAGTAGCGGTAGTGGTTC
QAEL01000003.1 ATTTTATTGATGTTTCATTAGATGATTGGTGTATGGATAAAACGCTACTTTTAGATAAGATTGATGAATTAAAAGATGAAGTAGCGGTAGTGGTTC
NZ_AAEQ01000046.1 ATTTTATTGATGTTTCATTAGATGATTGGTGTATGGATAAAACGCTACTTTTAGATAAGATTGATGAATTAAAAGATGAAGTAGCGGTAGTGGTTC
AMDT01000002.1 ATTTTATTGATGTTTCATTAGATGATTGGTGTATGGATAAAACGCTACTTTTAGATAAGATTGATGAATTAAAAGATGAAGTAGCGGTAGTGGTTC
JMPU01000016.1 ATTTTATTGATGTTTCATTAGATGATTGGTGTATGGATAAAACGCTACTTTTAGATAAGATTGATGAATTAAAAGATGAAGTAGCGGTAGTGGTTC
QAEM01000001.1 ATTTTATTGATGTTTCATTAGATGATTGGTGTATGGATAAAACGCTACTTTTAGATAAGATTGATGAATTAAAAGATGAAGTAGCGGTAGTGGTTC
QAEN01000002.1 ATTTTATTGATGTTTCATTAGATGATTGGTGTATGGATAAAACGCTACTTTTAGATAAGATTGATGAATTAAAAGATGAAGTAGCGGTAGTGGTTC
JHDS02000015.1 ATTTTATTGATGTTTCATTAGATGATTGGTGTATGGATAAAACGCTACTTTTAGATAAGATTGATGAATTAAAAGATGAAGTAGCGGTAGTGGTTC
PVLH01000002.1 ATTTTATTGATGTTTCATTAGATGATTGGTGTATGGATAAAACGCTACTTTTAGATAAGATTGATGAATTAAAAGATGAAGTAGCGGTAGTGGTTC
MVOA01000067.1 ATTTTATTGATGTTTCATTAGATGATTGGTGTATGGATAAAACGCTACTTTTAGATAAGATTGATGAATTAAAAGATGAAGTAGCGGTAGTGGTTC
QAEH01000002.1 ATTTTATTGATGTTTCATTAGATGATTGGTGTATGGATAAAACGCTACTTTTAGATAAGATTGATGAATTAAAAGATGAAGTAGCGGTAGTGGTTC
RQWM01000005.1 ATTTTATTGATGTTTCATTAGATGATTGGTGTATGGATAAAACGCTACTTTTAGATAAGATTGATGAATTAAAAGATGAAGTAGCGGTAGTGGTTC
LGCD01000038.1 ATTTTATTGATGTTTCATTAGATGATTGGTGTATGGATAAAACGCTACTTTTAGATAAGATTGATGAATTAAAAGATGAAGTAGCGGTAGTGGTTC
LGCC01000044.1 ATTTTATTGATGTTTCATTAGATGATTGGTGTATGGATAAAACGCTACTTTTAGATAAGATTGATGAATTAAAAGATGAAGTAGCAGTAGTGGTTC
Consensus ATTTTATTGATGTTTCATTAGATGATTGGTGTATGGATAAAACGCTACTTTTAGATAAGATTGATGAATTAAAAGATGAAGTAGCGGTAGTGGTTC
Conservation
3,940
3,960
3,980
4,000
4,020
NRIV01000001.1(Sterne) CGTATGCAACATTCGGATCATGGATGGATTTAAAAGAATATGAAGAATTAGAAAGAAAAGGCATTCCAGTTGTTGTAGACGCGGCACCAGGGTTTG
AAEO01000022.1 CGTATGCAACATTCGGATCATGGATGGATTTAAAAGAATATGAAGAATTAGAAAGAAAAGGCATTCCAGTTGTTGTAGACGCGGCACCAGGGTTTG
ALNY01000049.1 CGTATGCAACATTCGGATCATGGATGGATTTAAAAGAATATGAAGAATTAGAAAGAAAAGGCATTCCAGTTGTTGTAGACGCGGCACCAGGGTTTG
AGQP01000002.1 CGTATGCAACATTCGGATCATGGATGGATTTAAAAGAATATGAAGAATTAGAAAGAAAAGGCATTCCAGTTGTTGTAGACGCGGCACCAGGGTTTG
JSZS01000049.1 CGTATGCAACATTCGGATCATGGATGGATTTAAAAGAATATGAAGAATTAGAAAGAAAAGGCATTCCAGTTGTTGTAGACGCGGCACCAGGGTTTG
JTAG01000063.1 CGTATGCAACATTCGGATCATGGATGGATTTAAAAGAATATGAAGAATTAGAAAGAAAAGGCATTCCAGTTGTTGTAGACGCGGCACCAGGGTTTG
ABLH01000027.1 CGTATGCAACATTCGGATCATGGATGGATTTAAAAGAATATGAAGAATTAGAAAGAAAAGGCATTCCAGTTGTTGTAGACGCGGCACCAGGGTTTG
AAEN01000017.1 CGTATGCAACATTCGGATCATGGATGGATTTAAAAGAATATGAAGAATTAGAAAGAAAAGGCATTCCAGTTGTTGTAGACGCGGCACCAGGGTTTG
NZ_ABKG01000001.1 CGTATGCAACATTCGGATCATGGATGGATTTAAAAGAATATGAAGAATTAGAAAGAAAAGGCATTCCAGTTGTTGTAGACGCGGCACCAGGGTTTG
QAEL01000003.1 CGTATGCAACATTCGGATCATGGATGGATTTAAAAGAATATGAAGAATTAGAAAGAAAAGGCATTCCAGTTGTTGTAGACGCGGCACCAGGGTTTG
NZ_AAEQ01000046.1 CGTATGCAACATTCGGATCATGGATGGATTTAAAAGAATATGAAGAATTAGAAAGAAAAGGCATTCCAGTTGTTGTAGACGCGGCACCAGGGTTTG
AMDT01000002.1 CGTATGCAACATTCGGATCATGGATGGATTTAAAAGAATATGAAGAATTAGAAAGAAAAGGCATTCCAGTTGTTGTAGACGCGGCACCAGGGTTTG
JMPU01000016.1 CGTATGCAACATTCGGATCATGGATGGATTTAAAAGAATATGAAGAATTAGAAAGAAAAGGCATTCCAGTTGTTGTAGACGCGGCACCAGGGTTTG
QAEM01000001.1 CGTATGCAACATTCGGATCATGGATGGATTTAAAAGAATATGAAGAATTAGAAAGAAAAGGCATTCCAGTTGTTGTAGACGCGGCACCAGGGTTTG
QAEN01000002.1 CGTATGCAACATTCGGATCATGGATGGATTTAAAAGAATATGAAGAATTAGAAAGAAAAGGCATTCCAGTTGTTGTAGACGCGGCACCAGGGTTTG
JHDS02000015.1 CGTATGCAACATTCGGATCATGGATGGATTTAAAAGAATATGAAGAATTAGAAAGAAAAGGCATTCCAGTTGTTGTAGACGCGGCACCAGGGTTTG
PVLH01000002.1 CGTATGCAACATTCGGATCATGGATGGATTTAAAAGAATATGAAGAATTAGAAAGAAAAGGCATTCCAGTTGTTGTAGACGCGGCACCAGGGTTTG
MVOA01000067.1 CGTATGCAACATTCGGATCATGGATGGATTTAAAAGAATATGAAGAATTAGAAAGAAAAGGCATTCCAGTTGTTGTAGACGCGGCACCAGGGTTTG
QAEH01000002.1 CGTATGCAACATTCGGATCATGGATGGGTTTAAAAGAATATGAAGAATTAGAAAGAAAAGGCATTCCAGTTGTTGTAGACGCGGCACCAGGGTTTG
RQWM01000005.1 CGTATGCAACATTCGGATCATGGATGGATTTAAAAGAATATGAAGAATTAGAAAGAAAAGGCATTCCAGTTGTTGTAGACGCGGCACCAGGGTTTG
LGCD01000038.1 CGTATGCAACATTCGGATCATGGATGGATTTAAAAGAAGATAAAGAATTAGAAAGAAAAGGCATTCCAGTTGTTGTAGACGCGGCACCAGGGTTTG
LGCC01000044.1 CGTATGCAACATTCGGATCATGGATGGATTTAAAAGAAGATAAAGAATTAGAAAGAAAAGGCATTCCAGTTGTTGTAGACGCGGCACCAGGGTTTG
Consensus CGTATGCAACATTCGGATCATGGATGGATTTAAAAGAATATGAAGAATTAGAAAGAAAAGGCATTCCAGTTGTTGTAGACGCGGCACCAGGGTTTG
Conservation

Sequence	4,040 · 4,060 · 4,080 · 4,100 · 4,120
NRIV01000001.1(Sterne)	GGTTAATGCATGAGGGTATACATTACGGTCAGAATTTTAGTGGAATGATGGTATATAGCTTTCATGCAACAAAGCCTTTCGGAATTGGTGAAGGTG
AAEO01000022.1	GGTTAATGCATGAGGGTATACATTACGGTCAGAATTTTAGTGGAATGATGGTATATAGCTTTCATGCAACAAAGCCTTTCGGAATTGGTGAAGGTG
ALNY01000049.1	GGTTAATGCATGAGGGTATACATTACGGTCAGAATTTTAGTGGAATGATGGTATATAGCTTTCATGCAACAAAGCCTTTCGGAATTGGTGAAGGTG
AGQP01000002.1	GGTTAATGCATGAGGGTATACATTACGGTCAGAATTTTAGTGGAATGATGGTATATAGCTTTCATGCAACAAAGCCTTTCGGAATTGGTGAAGGTG
JSZS01000049.1	GGTTAATGCATGAGGGTATACATTACGGTCAGAATTTTAGTGGAATGATGGTATATAGCTTTCATGCAACAAAGCCTTTCGGAATTGGTGAAGGTG
JTAG01000063.1	GGTTAATGCATGAGGGTATACATTACGGTCAGAATTTTAGTGGAATGATGGTATATAGCTTTCATGCAACAAAGCCTTTCGGAATTGGTGAAGGTG
ABLH01000027.1	GGTTAATGCATGAGGGTATACATTACGGTCAGAATTTTAGTGGAATGATGGTATATAGCTTTCATGCAACAAAGCCTTTCGGAATTGGTGAAGGTG
AAEN01000017.1	GGTTAATGCATGAGGGTATACATTACGGTCAGAATTTTAGTGGAATGATGGTATATAGCTTTCATGCAACAAAGCCTTTCGGAATTGGTGAAGGTG
NZ_ABKG01000001.1	GGTTAATGCATGAGGGTATACATTACGGTCAGAATTTTAGTGGAATGATGGTATATAGCTTTCATGCAACAAAGCCTTTCGGAATTGGTGAAGGTG
QAEL01000003.1	GGTTAATGCATGAGGGTATACATTACGGTCAGAATTTTAGTGGAATGATGGTATATAGCTTTCATGCAACAAAGCCTTTCGGAATTGGTGAAGGTG
NZ_AAEQ01000046.1	GGTTAATGCATGAGGGTATACATTACGGTCAGAATTTTAGTGGAATGATGGTATATAGCTTTCATGCAACAAAGCCTTTCGGAATTGGTGAAGGTG
AMDT01000002.1	GGTTAATGCATGAGGGTATACATTACGGTCAGAATTTTAGTGGAATGATGGTATATAGCTTTCATGCAACAAAGCCTTTCGGAATTGGTGAAGGTG
JMPU01000016.1	GGTTAATGCATGAGGGTATACATTACGGTCAGAATTTTAGTGGAATGATGGTATATAGCTTTCATGCAACAAAGCCTTTCGGAATTGGTGAAGGTG
QAEM01000001.1	GGTTAATGCATGAGGGTATACATTACGGTCAGAATTTTAGTGGAATGATGGTATATAGCTTTCATGCAACAAAGCCTTTCGGAATTGGTGAAGGTG
QAEN01000002.1	GGTTAATGCATGAGGGTATACATTACGGTCAGAATTTTAGTGGAATGATGGTATATAGCTTTCATGCAACAAAGCCTTTCGGAATTGGTGAAGGTG
JHDS02000015.1	GGTTAATGCATGAGGGTATACATTACGGTCAGAATTTTAGTGGAATGATGGTATATAGCTTTCATGCAACAAAGCCTTTCGGAATTGGTGAAGGTG
PVLH01000002.1	GGTTAATGCATGAGGGTATACATTACGGTCAGAATTTTAGTGGAATGATGGTATATAGCTTTCATGCAACAAAGCCTTTCGGAATTGGTGAAGGTG
MVOA01000067.1	GGTTAATGCATGAGGGTATACATTACGGTCAGAATTTTAGTGGAATGATGGTATATAGCTTTCATGCAACAAAGCCTTTCGGAATTGGTGAAGGTG
QAEH01000002.1	GGTTAATGCATGAGGGTATACATTACGGTCAGAATTTTAGTGGAATGATTGTATATAGCTTTCATGCAACAAAGCCTTTCGGAATTGGTGAAGGTG
RQWM01000005.1	GGTTAATGCATGAGGGTATACATTACGGTCAGAATTTTAGTGGAATGATGGTATATAGCTTTCATGCAACAAAGCCTTTCGGAATTGGTGAAGGTG
LGCD01000038.1	GGTTAATGCATGAGGGTATACATTACGGTCAGAATTTTAGTGGAATGATGGTATATAGCTTTCATGCAACAAAGCCTTTCGGAATTGGTGAAGGTG
LGCC01000044.1	GGTTAATGCATGAGGGTATACATTACGGTCAGAATTTTAGTGGAATGATGGTATATAGCTTTCATGCAACAAAGCCTTTCGGAATTGGTGAAGGTG
Consensus	GGTTAATGCATGAGGGTATACATTACGGTCAGAATTTTAGTGGAATGATGGTATATAGCTTTCATGCAACAAAGCCTTTCGGAATTGGTGAAGGTG
Conservation	

Sequence	4,140 · 4,160 · 4,180 · 4,200 · 4,220
NRIV01000001.1(Sterne)	GTATGATATATAGCAATAATGAAGAAGAGATAAATCAAATAAAAAGAATGGGTAATTTTGGATTTGATCATAATCGTGAATGTACGATGATGGGGT
AAEO01000022.1	GTATGATATATAGCAATAATGAAGAAGAGATAAATCAAATAAAAAGAATGGGTAATTTTGGATTTGATCATAATCGTGAATGTACGATGATGGGGT
ALNY01000049.1	GTATGATATATAGCAATAATGAAGAAGAGATAAATCAAATAAAAAGAATGGGTAATTTTGGATTTGATCATAATCGTGAATGTACGATGATGGGGT
AGQP01000002.1	GTATGATATATAGCAATAATGAAGAAGAGATAAATCAAATAAAAAGAATGGGTAATTTTGGATTTGATCATAATCGTGAATGTACGATGATGGGGT
JSZS01000049.1	GTATGATATATAGCAATAATGAAGAAGAGATAAATCAAATAAAAAGAATGGGTAATTTTGGATTTGATCATAATCGTGAATGTACGATGATGGGGT
JTAG01000063.1	GTATGATATATAGCAATAATGAAGAAGAGATAAATCAAATAAAAAGAATGGGTAATTTTGGATTTGATCATAATCGTGAATGTACGATGATGGGGT
ABLH01000027.1	GTATGATATATAGCAATAATGAAGAAGAGATAAATCAAATAAAAAGAATGGGTAATTTTGGATTTGATCATAATCGTGAATGTACGATGATGGGGT
AAEN01000017.1	GTATGATATATAGCAATAATGAAGAAGAGATAAATCAAATAAAAAGAATGGGTAATTTTGGATTTGATCATAATCGTGAATGTACGATGATGGGGT
NZ_ABKG01000001.1	GTATGATATATAGCAATAATGAAGAAGAGATAAATCAAATAAAAAGAATGGGTAATTTTGGATTTGATCATAATCGTGAATGTACGATGATGGGGT
QAEL01000003.1	GTATGATATATAGCAATAATGAAGAAGAGATAAATCAAATAAAAAGAATGGGTAATTTTGGATTTGATCATAATCGTGAATGTACGATGATGGGGT
NZ_AAEQ01000046.1	GTATGATATATAGCAATAATGAAGAAGAGATAAATCAAATAAAAAGAATGGGTAATTTTGGATTTGATCATAATCGTGAATGTACGATGATGGGGT
AMDT01000002.1	GTATGATATATAGCAATAATGAAGAAGAGATAAATCAAATAAAAAGAATGGGTAATTTTGGATTTGATCATAATCGTGAATGTACGATGATGGGGT
JMPU01000016.1	GTATGATATATAGCAATAATGAAGAAGAGATAAATCAAATAAAAAGAATGGGTAATTTTGGATTTGATCATAATCGTGAATGTACGATGATGGGGT
QAEM01000001.1	GTATGATATATAGCAATAATGAAGAAGAGATAAATCAAATAAAAAGAATGGGTAATTTTGGATTTGATCATAATCGTGAATGTACGATGATGGGGT
QAEN01000002.1	GTATGATATATAGCAATAATGAAGAAGAGATAAATCAAATAAAAAGAATGGGTAATTTTGGATTTGATCATAATCGTGAATGTACGATGATGGGGT
JHDS02000015.1	GTATGATATATAGCAATAATGAAGAAGAGATAAATCAAATAAAAAGAATGGGTAATTTTGGATTTGATCATAATCGTGAATGTACGATGATGGGGT
PVLH01000002.1	GTATGATATATAGCAATAATGAAGAAGAGATAAATCAAATAAAAAGAATGGGTAATTTTGGATTTGATCATAATCGTGAATGTACGATGATGGGGT
MVOA01000067.1	GTATGATATATAGCAATAATGAAGAAGAGATAAATCAAATAAAAAGAATGGGTAATTTTGGATTTGATCATAATCGTGAATGTACGATGATGGGGT
QAEH01000002.1	GTATGATATATAGCAATAATGAAGAAGAGATAAATCAAATAAAAAGAATGGGTAATTTTGGATTTGATCACAATCGTGAATGTACGATGATGGGGT
RQWM01000005.1	GTATGATATATAGCAATAATGAAGAAGAGATAAATCAAATAAAAAGAATGGGTAATTTTGGATTTGATCATAATCGTGAATGTACGATGATGGGGT
LGCD01000038.1	GTATGAAAGATAACAATAAGGAAGAAGAGATAAAACAAATAAAAAGAATGGGTAATTTTGGATTTGATCATAATCGTGAATGTACGATGATGGGGT
LGCC01000044.1	GTATGAAAGATAACAATAAGGAAGAAGAGATAAAACAAATAAAAAGAATGGGTAATTTTGGATTTGATCATAATCGTGAATGTACGATGATGGGGT
Consensus	GTATGATATATAGCAATAATGAAGAAGAGATAAATCAAATAAAAAGAATGGGTAATTTTGGATTTGATCATAATCGTGAATGTACGATGATGGGGT
Conservation	

Sequence	4,240 · 4,260 · 4,280 · 4,300 · 4,
NRIV01000001.1(Sterne)	TTAACTGCAAAATGACTGAGTATGCAGCAGCTATTGGAATCGCAACGATGAATAAATGGAATCAAAAAGTGCAAGAACGAAATCGTATCTCCGAAT
AAEO01000022.1	TTAACTGCAAAATGACTGAGTATGCAGCAGCTATTGGAATCGCAACGATGAATAAATGGAATCAAAAAGTGCAAGAACGAAATCGTATCTCCGAAT
ALNY01000049.1	TTAACTGCAAAATGACTGAGTATGCAGCAGCTATTGGAATCGCAACGATGAATAAATGGAATCAAAAAGTGCAAGAACGAAATCGTATCTCCGAAT
AGQP01000002.1	TTAACTGCAAAATGACTGAGTATGCAGCAGCTATTGGAATCGCAACGATGAATAAATGGAATCAAAAAGTGCAAGAACGAAATCGTATCTCCGAAT
JSZS01000049.1	TTAACTGCAAAATGACTGAGTATGCAGCAGCTATTGGAATCGCAACGATGAATAAATGGAATCAAAAAGTGCAAGAACGAAATCGTATCTCCGAAT
JTAG01000063.1	TTAACTGCAAAATGACTGAGTATGCAGCAGCTATTGGAATCGCAACGATGAATAAATGGAATCAAAAAGTGCAAGAACGAAATCGTATCTCCGAAT
ABLH01000027.1	TTAACTGCAAAATGACTGAGTATGCAGCAGCTATTGGAATCGCAACGATGAATAAATGGAATCAAAAAGTGCAAGAACGAAATCGTATCTCCGAAT
AAEN01000017.1	TTAACTGCAAAATGACTGAGTATGCAGCAGCTATTGGAATCGCAACGATGAATAAATGGAATCAAAAAGTGCAAGAACGAAATCGTATCTCCGAAT
NZ_ABKG01000001.1	TTAACTGCAAAATGACTGAGTATGCAGCAGCTATTGGAATCGCAACGATGAATAAATGGAATCAAAAAGTGCAAGAACGAAATCGTATCTCCGAAT
QAEL01000003.1	TTAACTGCAAAATGACTGAGTATGCAGCAGCTATTGGAATCGCAACGATGAATAAATGGAATCAAAAAGTGCAAGAACGAAATCGTATCTCCGAAT
NZ_AAEQ01000046.1	TTAACTGCAAAATGACTGAGTATGCAGCAGCTATTGGAATCGCAACGATGAATAAATGGAATCAAAAAGTGCAAGAACGAAATCGTATCTCCGAAT
AMDT01000002.1	TTAACTGCAAAATGACTGAGTATGCAGCAGCTATTGGAATCGCAACGATGAATAAATGGAATCAAAAAGTGCAAGAACGAAATCGTATCTCCGAAT
JMPU01000016.1	TTAACTGCAAAATGACTGAGTATGCAGCAGCTATTGGAATCGCAACGATGAATAAATGGAATCAAAAAGTGCAAGAACGAAATCGTATCTCCGAAT
QAEM01000001.1	TTAACTGCAAAATGACTGAGTATGCAGCAGCTATTGGAATCGCAACGATGAATAAATGGAATCAAAAAGTGCAAGAACGAAATCGTATCTCCGAAT
QAEN01000002.1	TTAACTGCAAAATGACTGAGTATGCAGCAGCTATTGGAATCGCAACGATGAATAAATGGAATCAAAAAGTGCAAGAACGAAATCGTATCTCCGAAT
JHDS02000015.1	TTAACTGCAAAATGACTGAGTATGCAGCAGCTATTGGAATCGCAACGATGAATAAATGGAATCAAAAAGTGCAAGAACGAAATCGTATCTCCGAAT
PVLH01000002.1	TTAACTGCAAAATGACTGAGTATGCAGCAGCTATTGGAATCGCAACGATGAATAAATGGAATCAAAAAGTGCAAGAACGAAATCGTATCTCCGAAT
MVOA01000067.1	TTAACTGCAAAATGACTGAGTATGCAGCAGCTATTGGAATCGCAACGATGAATAAATGGAATCAAAAAGTGCAAGAACGAAATCGTATCTCCGAAT
QAEH01000002.1	TTAACTGCAAAATGACTGAGTATGCAGCAGCTATTGGAATCGCAACGATGAATAAATGGAATCAAAAAGTGCAAGAACGAAATCGTATCTCCGAAT
RQWM01000005.1	TTAACTGCAAAATGACTGAGTATGCAGCAGCTATTGGAATCGCAACGATGAATAAATGGAATCAAAAAGTGCAAGAACGAAATCGTATCTCCGAAT
LGCD01000038.1	TTAACTGCAAAATGACTGAGTATGCAGCAGCTATTGGAATCGCAACGATGAATAAATGGAATCAAAAAGTGCAAGAACGAAATCGTATCTCCGAAT
LGCC01000044.1	TTAACTGCAAAATGACTGAGTATGCAGCAGCTATTGGAATCGCAACGATGAATAAATGGAATCAAAAAGTGCAAGAACGAAATCGTATCTCCGAAT
Consensus	TTAACTGCAAAATGACTGAGTATGCAGCAGCTATTGGAATCGCAACGATGAATAAATGGAATCAAAAAGTGCAAGAACGAAATCGTATCTCCGAAT
Conservation	

```
                          ,320                4,340               4,360               4,380               4,400
NRIV01000001.1(Sterne) GGTATACACAGTTATTACAAAGTACAGGATTAATGAAAAAAGGATGGGAAGTTCAAAAGACAGAAGCAGTTATTCATCAATTTATGCCTATTCTCT
AAEO01000022.1         GGTATACACAGTTATTACAAAGTACAGGATTAATGAAAAAAGGATGGGAAGTTCAAAAGACAGAAGCAGTTATTCATCAATTTATGCCTATTCTCT
ALNY01000049.1         GGTATACACAGTTATTACAAAGTACAGGATTAATGAAAAAAGGATGGGAAGTTCAAAAGACAGAAGCAGTTATTCATCAATTTATGCCTATTCTCT
AGQP01000002.1         GGTATACACAGTTATTACAAAGTACAGGATTAATGAAAAAAGGATGGGAAGTTCAAAAGACAGAAGCAGTTATTCATCAATTTATGCCTATTCTCT
JSZS01000049.1         GGTATACACAGTTATTACAAAGTACAGGATTAATGAAAAAAGGATGGGAAGTTCAAAAGACAGAAGCAGTTATTCATCAATTTATGCCTATTCTCT
JTAG01000063.1         GGTATACACAGTTATTACAAAGTACAGGATTAATGAAAAAAGGATGGGAAGTTCAAAAGACAGAAGCAGTTATTCATCAATTTATGCCTATTCTCT
ABLH01000027.1         GGTATACACAGTTATTACAAAGTACAGGATTAATGAAAAAAGGATGGGAAGTTCAAAAGACAGAAGCAGTTATTCATCAATTTATGCCTATTCTCT
AAEN01000017.1         GGTATACACAGTTATTACAAAGTACAGGATTAATGAAAAAAGGATGGGAAGTTCAAAAGACAGAAGCAGTTATTCATCAATTTATGCCTATTCTCT
NZ_ABKG01000001.1      GGTATACACAGTTATTACAAAGTACAGGATTAATGAAAAAAGGATGGGAAGTTCAAAAGACAGAAGCAGTTATTCATCAATTTATGCCTATTCTCT
QAEL01000003.1         GGTATACACAGTTATTACAAAGTACAGGATTAATGAAAAAAGGATGGGAAGTTCAAAAGACAGAAGCAGTTATTCATCAATTTATGCCTATTCTCT
NZ_AAEQ01000046.1      GGTATACACAGTTATTACAAAGTACAGGATTAATGAAAAAAGGATGGGAAGTTCAAAAGACAGAAGCAGTTATTCATCAATTTATGCCTATTCTCT
AMDT01000002.1         GGTATACACAGTTATTACAAAGTACAGGATTAATGAAAAAAGGATGGGAAGTTCAAAAGACAGAAGCAGTTATTCATCAATTTATGCCTATTCTCT
JMPU01000016.1         GGTATACACAGTTATTACAAAGTACAGGATTAATGAAAAAAGGATGGGAAGTTCAAAAGACAGAAGCAGTTATTCATCAATTTATGCCTATTCTCT
QAEM01000001.1         GGTATACACAGTTATTACAAAGTACAGGATTAATGAAAAAAGGATGGGAAGTTCAAAAGACAGAAGCAGTTATTCATCAATTTATGCCTATTCTCT
QAEN01000002.1         GGTATACACAGTTATTACAAAGTACAGGATTAATGAAAAAAGGATGGGAAGTTCAAAAGACAGAAGCAGTTATTCATCAATTTATGCCTATTCTCT
JHDS02000015.1         GGTATACACAGTTATTACAAAGTACAGGATTAATGAAAAAAGGATGGGAAGTTCAAAAGACAGAAGCAGTTATTCATCAATTTATGCCTATTCTCT
PVLH01000002.1         GGTATACACAGTTATTACAAAGTACAGGATTAATGAAAAAAGGATGGGAAGTTCAAAAGACAGAAGCAGTTATTCATCAATTTATGCCTATTCTCT
MVOA01000067.1         GGTATACACAGTTATTACAAAGTACAGGATTAATGAAAAAAGGATGGGAAGTTCAAAAGACAGAAGCAGTTATTCATCAATTTATGCCTATTCTCT
QAEH01000002.1         GGTATACACAGTTATTACAAAGTACAGGATTAATGAAAAAAGGATGGGAAGTTCAAAAGACAGAAGCAGTTATTCATCAATTTATGCCTATTCTCT
RQWM01000005.1         GGTATACACAGTTATTACAAAGTACAGGATTAATGAAAAAAGGATGGGAAGTTCAAAAGACAGAAGCAGTTATTCATCAATTTATGCCTATTCTCT
LGCD01000038.1         GGTATACACAGTTATTACAAAGTACAGGATTAATGAAAAAAGGATGGGAAGTTCAAAAGACAGAAGCAGTTATTCATCAATTTATGCCTATTCTCT
LGCC01000044.1         GGTATACACAGTTATTACAAAGTACAGGATTAATGAAAAAAGGATGGGAAGTTCAAAAGACAGAAGCAGTTATTCATCAATTTATGCCTATTCTCT
Consensus              GGTATACACAGTTATTACAAAGTACAGGATTAATGAAAAAAGGATGGGAAGTTCAAAAGACAGAAGCAGTTATTCATCAATTTATGCCTATTCTCT
Conservation
```

```
                          4,420               4,440               4,460               4,480               4,500
NRIV01000001.1(Sterne) GCCCAGAAAAAAATTCACAATACGCAAGTAATAGAAGTATTGAAAAAGCAAAATATAGAAGCTAGATTATATTTTTCACCATCTTGTCACCAACAAG
AAEO01000022.1         GCCCAGAAAAAAATTCACAATACGCAAGTAATAGAAGTATTGAAAAAGCAAAATATAGAAGCTAGATTATATTTTTCACCATCTTGTCACCAACAAG
ALNY01000049.1         GCCCAGAAAAAAATTCACAATACGCAAGTAATAGAAGTATTGAAAAAGCAAAATATAGAAGCTAGATTATATTTTTCACCATCTTGTCACCAACAAG
AGQP01000002.1         GCCCAGAAAAAAATTCACAATACGCAAGTAATAGAAGTATTGAAAAAGCAAAATATAGAAGCTAGATTATATTTTTCACCATCTTGTCACCAACAAG
JSZS01000049.1         GCCCAGAAAAAAATTCACAATACGCAAGTAATAGAAGTATTGAAAAAGCAAAATATAGAAGCTAGATTATATTTTTCACCATCTTGTCACCAACAAG
JTAG01000063.1         GCCCAGAAAAAAATTCACAATACGCAAGTAATAGAAGTATTGAAAAAGCAAAATATAGAAGCTAGATTATATTTTTCACCATCTTGTCACCAACAAG
ABLH01000027.1         GCCCAGAAAAAAATTCACAATACGCAAGTAATAGAAGTATTGAAAAAGCAAAATATAGAAGCTAGATTATATTTTTCACCATCTTGTCACCAACAAG
AAEN01000017.1         GCCCAGAAAAAAATTCACAATACGCAAGTAATAGAAGTATTGAAAAAGCAAAATATAGAAGCTAGATTATATTTTTCACCATCTTGTCACCAACAAG
NZ_ABKG01000001.1      GCCCAGAAAAAAATTCACAATACGCAAGTAATAGAAGTATTGAAAAAGCAAAATATAGAAGCTAGATTATATTTTTCACCATCTTGTCACCAACAAG
QAEL01000003.1         GCCCAGAAAAAAATTCACAATACGCAAGTAATAGAAGTATTGAAAAAGCAAAATATAGAAGCTAGATTATATTTTTCACCATCTTGTCACCAACAAG
NZ_AAEQ01000046.1      GCCCAGAAAAAAATTCACAATACGCAAGTAATAGAAGTATTGAAAAAGCAAAATATAGAAGCTAGATTATATTTTTCACCATCTTGTCACCAACAAG
AMDT01000002.1         GCCCAGAAAAAAATTCACAATACGCAAGTAATAGAAGTATTGAAAAAGCAAAATATAGAAGCTAGATTATATTTTTCACCATCTTGTCACCAACAAG
JMPU01000016.1         GCCCAGAAAAAAATTCACAATACGCAAGTAATAGAAGTATTGAAAAAGCAAAATATAGAAGCTAGATTATATTTTTCACCATCTTGTCACCAACAAG
QAEM01000001.1         GCCCAGAAAAAAATTCACAATACGCAAGTAATAGAAGTATTGAAAAAGCAAAATATAGAAGCTAGATTATATTTTTCACCATCTTGTCACCAACAAG
QAEN01000002.1         GCCCAGAAAAAAATTCACAATACGCAAGTAATAGAAGTATTGAAAAAGCAAAATATAGAAGCTAGATTATATTTTTCACCATCTTGTCACCAACAAG
JHDS02000015.1         GCCCAGAAAAAAATTCACAATACGCAAGTAATAGAAGTATTGAAAAAGCAAAATATAGAAGCTAGATTATATTTTTCACCATCTTGTCACCAACAAG
PVLH01000002.1         GCCCAGAAAAAAATTCACAATACGCAAGTAATAGAAGTATTGAAAAAGCAAAATATAGAAGCTAGATTATATTTTTCACCATCTTGTCACCAACAAG
MVOA01000067.1         GCCCAGAAAAAAATTCACAATACGCAAGTAATAGAAGTATTGAAAAAGCAAAATATAGAAGCTAGATTATATTTTTCACCATCTTGTCACCAACAAG
QAEH01000002.1         GCCCAGAAAAAAATTCACAATACGCAAGTAATAGAAGTATTGAAAAAGCAAAATATAGAAGCTAGATTATATTTTTCCCCATCTTGTCACCAACAAG
RQWM01000005.1         GCCCAGAAAAAAATTCACAATACGCAAGTAATAGAAGTATTGAAAAAGCAAAATATAGAAGCTAGATTATATTTTTCACCATCTTGTCACCAACAAG
LGCD01000038.1         GCCCAGAAAAAAATTCACAATACGCAAGTAATAGAAGTATTGAAAAAGCAAAATATAGAAGCTAGATTATATTTTTCACCATCTTGTCACCAACAAG
LGCC01000044.1         GCCCAGAAAAAAATTCACAATACGCAAGTAATAGAAGTATTGAAAAAGCAAAATATAGAAGCTAGATTATATTTTTCACCATCTTGTCACCAACAAG
Consensus              GCCCAGAAAAAAATTCACAATACGCAAGTAATAGAAGTATTGAAAAAGCAAAATATAGAAGCTAGATTATATTTTTCACCATCTTGTCACCAACAAG
Conservation
```

```
                              4,520               4,540               4,560               4,580               4,600
NRIV01000001.1(Sterne) ATTTCTTTAAACACTATAAATCTACAGATTTAACAAATACAAATAAAATAGCAAAACAAATAATTAGTCTACCTTTATGGGAAGGAATGACGCAAG
AAEO01000022.1         ATTTCTTTAAACACTATAAATCTACAGATTTAACAAATACAAATAAAATAGCAAAACAAATAATTAGTCTACCTTTATGGGAAGGAATGACGCAAG
ALNY01000049.1         ATTTCTTTAAACACTATAAATCTACAGATTTAACAAATACAAATAAAATAGCAAAACAAATAATTAGTCTACCTTTATGGGAAGGAATGACGCAAG
AGQP01000002.1         ATTTCTTTAAACACTATAAATCTACAGATTTAACAAATACAAATAAAATAGCAAAACAAATAATTAGTCTACCTTTATGGGAAGGAATGACGCAAG
JSZS01000049.1         ATTTCTTTAAACACTATAAATCTACAGATTTAACAAATACAAATAAAATAGCAAAACAAATAATTAGTCTACCTTTATGGGAAGGAATGACGCAAG
JTAG01000063.1         ATTTCTTTAAACACTATAAATCTACAGATTTAACAAATACAAATAAAATAGCAAAACAAATAATTAGTCTACCTTTATGGGAAGGAATGACGCAAG
ABLH01000027.1         ATTTCTTTAAACACTATAAATCTACAGATTTAACAAATACAAATAAAATAGCAAAACAAATAATTAGTCTACCTTTATGGGAAGGAATGACGCAAG
AAEN01000017.1         ATTTCTTTAAACACTATAAATCTACAGATTTAACAAATACAAATAAAATAGCAAAACAAATAATTAGTCTACCTTTATGGGAAGGAATGACGCAAG
NZ_ABKG01000001.1      ATTTCTTTAAACACTATAAATCTACAGATTTAACAAATACAAATAAAATAGCAAAACAAATAATTAGTCTACCTTTATGGGAAGGAATGACGCAAG
QAEL01000003.1         ATTTCTTTAAACACTATAAATCTACAGATTTAACAAATACAAATAAAATAGCAAAACAAATAATTAGTCTACCTTTATGGGAAGGAATGACGCAAG
NZ_AAEQ01000046.1      ATTTCTTTAAACACTATAAATCTACAGATTTAACAAATACAAATAAAATAGCAAAACAAATAATTAGTCTACCTTTATGGGAAGGAATGACGCAAG
AMDT01000002.1         ATTTCTTTAAACACTATAAATCTACAGATTTAACAAATACAAATAAAATAGCAAAACAAATAATTAGTCTACCTTTATGGGAAGGAATGACGCAAG
JMPU01000016.1         ATTTCTTTAAACACTATAAATCTACAGATTTAACAAATACAAATAAAATAGCAAAACAAATAATTAGTCTACCTTTATGGGAAGGAATGACGCAAG
QAEM01000001.1         ATTTCTTTAAACACTATAAATCTACAGATTTAACAAATACAAATAAAATAGCAAAACAAATAATTAGTCTACCTTTATGGGAAGGAATGACGCAAG
QAEN01000002.1         ATTTCTTTAAACACTATAAATCTACAGATTTAACAAATACAAATAAAATAGCAAAACAAATAATTAGTCTACCTTTATGGGAAGGAATGACGCAAG
JHDS02000015.1         ATTTCTTTAAACACTATAAATCTACAGATTTAACAAATACAAATAAAATAGCAAAACAAATAATTAGTCTACCTTTATGGGAAGGAATGACGCAAG
PVLH01000002.1         ATTTCTTTAAACACTATAAATCTACAGATTTAACAAATACAAATAAAATAGCAAAACAAATAATTAGTCTACCTTTATGGGAAGGAATGACGCAAG
MVOA01000067.1         ATTTCTTTAAACACTATAAATCTACAGATTTAACAAATACAAATAAAATAGCAAAACAAATAATTAGTCTACCTTTATGGGAAGGAATGACGCAAG
QAEH01000002.1         ATTTCTTTAAACACTATAAATCTACAGATTTAACAAATACAAATAAAATAGCAAAACAAATAATTAGTCTACCTTTATGGGAAGGAATGACGCAAG
RQWM01000005.1         ATTTCTTTAAACACTATAAATCTACAGATTTAACAAATACAAATAAAATAGCAAAACAAATAATTAGTCTACCTTTATGGGAAGGAATGACGCAAG
LGCD01000038.1         ATTTCTTTAAACACTATAAATATACAGATTTAACAAATACAAATAAAATAGCAAAACAAATAATTAGTCTACCTTTATGGGAAGGAATGACGCAAG
LGCC01000044.1         ATTTCTTTAAACACTATAAATATACAGATTTAACAAATACAAATAAAATAGCAAAACAAATAATTAGTCTACCTTTATGGGAAGGAATGACGCAAG
Consensus              ATTTCTTTAAACACTATAAATCTACAGATTTAACAAATACAAATAAAATAGCAAAACAAATAATTAGTCTACCTTTATGGGAAGGAATGACGCAAG
Conservation
```

```
                                 4,620                4,640                4,660                4,680                4,700
NRIV01000001.1(Sterne) AAATAGTGGAGCAAATTATAATGTGTTTAATGCAGAAGGTGGTGTCGACAGATGAATAGTTTTTATAGTCAAGAAGAGCTAAAGAAAATTGGATTT
AAEO01000022.1         AAATAGTGGAGCAAATTATAATGTGTTTAATGCAGAAGGTGGTGTCGACAGATGAATAGTTTTTATAGTCAAGAAGAGCTAAAGAAAATTGGATTT
ALNY01000049.1         AAATAGTGGAGCAAATTATAATGTGTTTAATGCAGAAGGTGGTGTCGACAGATGAATAGTTTTTATAGTCAAGAAGAGCTAAAGAAAATTGGATTT
AGQP01000002.1         AAATAGTGGAGCAAATTATAATGTGTTTAATGCAGAAGGTGGTGTCGACAGATGAATAGTTTTTATAGTCAAGAAGAGCTAAAGAAAATTGGATTT
JSZS01000049.1         AAATAGTGGAGCAAATTATAATGTGTTTAATGCAGAAGGTGGTGTCGACAGATGAATAGTTTTTATAGTCAAGAAGAGCTAAAGAAAATTGGATTT
JTAG01000063.1         AAATAGTGGAGCAAATTATAATGTGTTTAATGCAGAAGGTGGTGTCGACAGATGAATAGTTTTTATAGTCAAGAAGAGCTAAAGAAAATTGGATTT
ABLH01000027.1         AAATAGTGGAGCAAATTATAATGTGTTTAATGCAGAAGGTGGTGTCGACAGATGAATAGTTTTTATAGTCAAGAAGAGCTAAAGAAAATTGGATTT
AAEN01000017.1         AAATAGTGGAGCAAATTATAATGTGTTTAATGCAGAAGGTGGTGTCGACAGATGAATAGTTTTTATAGTCAAGAAGAGCTAAAGAAAATTGGATTT
NZ_ABKG01000001.1      AAATAGTGGAGCAAATTATAATGTGTTTAATGCAGAAGGTGGTGTCGACAGATGAATAGTTTTTATAGTCAAGAAGAGCTAAAGAAAATTGGATTT
QAEL01000003.1         AAATAGTGGAGCAAATTATAATGTGTTTAATGCAGAAGGTGGTGTCGACAGATGAATAGTTTTTATAGTCAAGAAGAGCTAAAGAAAATTGGATTT
NZ_AAEQ01000046.1      AAATAGTGGAGCAAATTATAATGTGTTTAATGCAGAAGGTGGTGTCGACAGATGAATAGTTTTTATAGTCAAGAAGAGCTAAAGAAAATTGGATTT
AMDT01000002.1         AAATAGTGGAGCAAATTATAATGTGTTTAATGCAGAAGGTGGTGTCGACAGATGAATAGTTTTTATAGTCAAGAAGAGCTAAAGAAAATTGGATTT
JMPU01000016.1         AAATAGTGGAGCAAATTATAATGTGTTTAATGCAGAAGGTGGTGTCGACAGATGAATAGTTTTTATAGTCAAGAAGAGCTAAAGAAAATTGGATTT
QAEM01000001.1         AAATAGTGGAGCAAATTATAATGTGTTTAATGCAGAAGGTGGTGTCGACAGATGAATAGTTTTTATAGTCAAGAAGAGCTAAAGAAAATTGGATTT
QAEN01000002.1         AAATAGTGGAGCAAATTATAATGTGTTTAATGCAGAAGGTGGTGTCGACAGATGAATAGTTTTTATAGTCAAGAAGAGCTAAAGAAAATTGGATTT
JHDS02000015.1         AAATAGTGGAGCAAATTATAATGTGTTTAATGCAGAAGGTGGTGTCGACAGATGAATAGTTTTTATAGTCAAGAAGAGCTAAAGAAAATTGGATTT
PVLH01000002.1         AAATAGTGGAGCAAATTATAATGTGTTTAATGCAGAAGGTGGTGTCGACAGATGAATAGTTTTTATAGTCAAGAAGAGCTAAAGAAAATTGGATTT
MVOA01000067.1         AAATAGTGGAGCAAATTATAATGTGTTTAATGCAGAAGGTGGTGTCGACAGATGAATAGTTTTTATAGTCAAGAAGAGCTAAAGAAAATTGGATTT
QAEH01000002.1         AAATAGTGGAGCAAATTATAATGTGTTTAATGCAGAAGGTGGTGTCGACAGATGAATAGTTTTTATAGTCAAGAAGAGCTAAAGAAAATTGGATTT
RQWM01000005.1         AAATAGTGGAGCAAATTATAATGTGTTTAATGCAGAAGGTGGTGTCGACAGATGAATAGTTTTTATAGTCAAGAAGAGCTAAAGAAAATTGGATTT
LGCD01000038.1         AAATAGTGGAGCAAATTATAATGTGTTTAATGCAGAAGGTGGTGTCGACAGATGAATAGTTTTTATAGTCAAGAAGAGCTAAAGAAAATTGGATTT
LGCC01000044.1         AAATAGTGGAGCAAATTATAATGTGTTTAATGCAGAAGGTGGTGTCGACAGATGAATAGTTTTTATAGTCAAGAAGAGCTAAAGAAAATTGGATTT
Consensus              AAATAGTGGAGCAAATTATAATGTGTTTAATGCAGAAGGTGGTGTCGACAGATGAATAGTTTTTATAGTCAAGAAGAGCTAAAGAAAATTGGATTT
Conservation
```

```
                                     4,720                4,740                4,760                4,780                4,
NRIV01000001.1(Sterne) TTATCTGTTGGGAAAAATGTATTGATTAGTAAAAAAGCAAGTATATATAATCCAGGTGTTATATCAGTTGGTAATAATGTAAGGATTGATGACTTT
AAEO01000022.1         TTATCTGTTGGGAAAAATGTATTGATTAGTAAAAAAGCAAGTATATATAATCCAGGTGTTATATCAGTTGGTAATAATGTAAGGATTGATGACTTT
ALNY01000049.1         TTATCTGTTGGGAAAAATGTATTGATTAGTAAAAAAGCAAGTATATATAATCCAGGTGTTATATCAGTTGGTAATAATGTAAGGATTGATGACTTT
AGQP01000002.1         TTATCTGTTGGGAAAAATGTATTGATTAGTAAAAAAGCAAGTATATATAATCCAGGTGTTATATCAGTTGGTAATAATGTAAGGATTGATGACTTT
JSZS01000049.1         TTATCTGTTGGGAAAAATGTATTGATTAGTAAAAAAGCAAGTATATATAATCCAGGTGTTATATCAGTTGGTAATAATGTAAGGATTGATGACTTT
JTAG01000063.1         TTATCTGTTGGGAAAAATGTATTGATTAGTAAAAAAGCAAGTATATATAATCCAGGTGTTATATCAGTTGGTAATAATGTAAGGATTGATGACTTT
ABLH01000027.1         TTATCTGTTGGGAAAAATGTATTGATTAGTAAAAAAGCAAGTATATATAATCCAGGTGTTATATCAGTTGGTAATAATGTAAGGATTGATGACTTT
AAEN01000017.1         TTATCTGTTGGGAAAAATGTATTGATTAGTAAAAAAGCAAGTATATATAATCCAGGTGTTATATCAGTTGGTAATAATGTAAGGATTGATGACTTT
NZ_ABKG01000001.1      TTATCTGTTGGGAAAAATGTATTGATTAGTAAAAAAGCAAGTATATATAATCCAGGTGTTATATCAGTTGGTAATAATGTAAGGATTGATGACTTT
QAEL01000003.1         TTATCTGTTGGGAAAAATGTATTGATTAGTAAAAAAGCAAGTATATATAATCCAGGTGTTATATCAGTTGGTAATAATGTAAGGATTGATGACTTT
NZ_AAEQ01000046.1      TTATCTGTTGGGAAAAATGTATTGATTAGTAAAAAAGCAAGTATATATAATCCAGGTGTTATATCAGTTGGTAATAATGTAAGGATTGATGACTTT
AMDT01000002.1         TTATCTGTTGGGAAAAATGTATTGATTAGTAAAAAAGCAAGTATATATAATCCAGGTGTTATATCAGTTGGTAATAATGTAAGGATTGATGACTTT
JMPU01000016.1         TTATCTGTTGGGAAAAATGTATTGATTAGTAAAAAAGCAAGTATATATAATCCAGGTGTTATATCAGTTGGTAATAATGTAAGGATTGATGACTTT
QAEM01000001.1         TTATCTGTTGGGAAAAATGTATTGATTAGTAAAAAAGCAAGTATATATAATCCAGGTGTTATATCAGTTGGTAATAATGTAAGGATTGATGACTTT
QAEN01000002.1         TTATCTGTTGGGAAAAATGTATTGATTAGTAAAAAAGCAAGTATATATAATCCAGGTGTTATATCAGTTGGTAATAATGTAAGGATTGATGACTTT
JHDS02000015.1         TTATCTGTTGGGAAAAATGTATTGATTAGTAAAAAAGCAAGTATATATAATCCAGGTGTTATATCAGTTGGTAATAATGTAAGGATTGATGACTTT
PVLH01000002.1         TTATCTGTTGGGAAAAATGTATTGATTAGTAAAAAAGCAAGTATATATAATCCAGGTGTTATATCAGTTGGTAATAATGTAAGGATTGATGACTTT
MVOA01000067.1         TTATCTGTTGGGAAAAATGTATTGATTAGTAAAAAAGCAAGTATATATAATCCAGGTGTTATATCAGTTGGTAATAATGTAAGGATTGATGACTTT
QAEH01000002.1         TTATCTGTTGGGAAAAATGTATTGATTAGTAAAAAAGCAAGTATATATAATCCAGGTGTTATATCAGTTGGTAATAATGTAAGGATTGATGACTTT
RQWM01000005.1         TTATCTGTTGGGAAAAATGTATTGATTAGTAAAAAAGCAAGTATATATAATCCAGGTGTTATATCAGTTGGTAATAATGTAAGGATTGATGACTTT
LGCD01000038.1         TTATCTGTTGGGAAAAATGTATTGATTAGTAAAAAAGCAAGTATATATAATCCAGGTGTTATATCAGTTGGTAATAATGTAAGGATTGATGACTTT
LGCC01000044.1         TTATCTGTTGGGAAAAATGTATTGATTAGTAAAAAAGCAAGTATATATAATCCAGGTGCTACATCAGTTCATAAGAATGTAAGGATTGATGACTTT
Consensus              TTATCTGTTGGGAAAAATGTATTGATTAGTAAAAAAGCAAGTATATATAATCCAGGTGTTATATCAGTTGGTAATAATGTAAGGATTGATGACTTT
Conservation
```

```
                       ,800                 4,820                4,840                4,860                4,880
NRIV01000001.1(Sterne) TGTATTTTAAGTGGCAAAGTAACGATTGGAAGTTACTCGCATATAGCAGCGTATACAGCGTTATATGGAGGGGAAGTTGGGATTGAAATGTATGAC
AAEO01000022.1         TGTATTTTAAGTGGCAAAGTAACGATTGGAAGTTACTCGCATATAGCAGCGTATACAGCGTTATATGGAGGGGAAGTTGGGATTGAAATGTATGAC
ALNY01000049.1         TGTATTTTAAGTGGCAAAGTAACGATTGGAAGTTACTCGCATATAGCAGCGTATACAGCGTTATATGGAGGGGAAGTTGGGATTGAAATGTATGAC
AGQP01000002.1         TGTATTTTAAGTGGCAAAGTAACGATTGGAAGTTACTCGCATATAGCAGCGTATACAGCGTTATATGGAGGGGAAGTTGGGATTGAAATGTATGAC
JSZS01000049.1         TGTATTTTAAGTGGCAAAGTAACGATTGGAAGTTACTCGCATATAGCAGCGTATACAGCGTTATATGGAGGGGAAGTTGGGATTGAAATGTATGAC
JTAG01000063.1         TGTATTTTAAGTGGCAAAGTAACGATTGGAAGTTACTCGCATATAGCAGCGTATACAGCGTTATATGGAGGGGAAGTTGGGATTGAAATGTATGAC
ABLH01000027.1         TGTATTTTAAGTGGCAAAGTAACGATTGGAAGTTACTCGCATATAGCAGCGTATACAGCGTTATATGGAGGGGAAGTTGGGATTGAAATGTATGAC
AAEN01000017.1         TGTATTTTAAGTGGCAAAGTAACGATTGGAAGTTACTCGCATATAGCAGCGTATACAGCGTTATATGGAGGGGAAGTTGGGATTGAAATGTATGAC
NZ_ABKG01000001.1      TGTATTTTAAGTGGCAAAGTAACGATTGGAAGTTACTCGCATATAGCAGCGTATACAGCGTTATATGGAGGGGAAGTTGGGATTGAAATGTATGAC
QAEL01000003.1         TGTATTTTAAGTGGCAAAGTAACGATTGGAAGTTACTCGCATATAGCAGCGTATACAGCGTTATATGGAGGGGAAGTTGGGATTGAAATGTATGAC
NZ_AAEQ01000046.1      TGTATTTTAAGTGGCAAAGTAACGATTGGAAGTTACTCGCATATAGCAGCGTATACAGCGTTATATGGAGGGGAAGTTGGGATTGAAATGTATGAC
AMDT01000002.1         TGTATTTTAAGTGGCAAAGTAACGATTGGAAGTTACTCGCATATAGCAGCGTATACAGCGTTATATGGAGGGGAAGTTGGGATTGAAATGTATGAC
JMPU01000016.1         TGTATTTTAAGTGGCAAAGTAACGATTGGAAGTTACTCGCATATAGCAGCGTATACAGCGTTATATGGAGGGGAAGTTGGGATTGAAATGTATGAC
QAEM01000001.1         TGTATTTTAAGTGGCAAAGTAACGATTGGAAGTTACTCGCATATAGCAGCGTATACAGCGTTATATGGAGGGGAAGTTGGGATTGAAATGTATGAC
QAEN01000002.1         TGTATTTTAAGTGGCAAAGTAACGATTGGAAGTTACTCGCATATAGCAGCGTATACAGCGTTATATGGAGGGGAAGTTGGGATTGAAATGTATGAC
JHDS02000015.1         TGTATTTTAAGTGGCAAAGTAACGATTGGAAGTTACTCGCATATAGCAGCGTATACAGCGTTATATGGAGGGGAAGTTGGGATTGAAATGTATGAC
PVLH01000002.1         TGTATTTTAAGTGGCAAAGTAACGATTGGAAGTTACTCGCATATAGCAGCGTATACAGCGTTATATGGAGGGGAAGTTGGGATTGAAATGTATGAC
MVOA01000067.1         TGTATTTTAAGTGGCAAAGTAACGATTGGAAGTTACTCGCATATAGCAGCGTATACAGCGTTATATGGAGGGGAAGTTGGGATTGAAATGTATGAC
QAEH01000002.1         TGTATTTTAAGTGGCAAAGTAACGATTGGAAGTTACTCGCATATAGCAGCGTATACAGCGTTATATGGAGGGGAAGTTGGGATTGAAATGTATGAC
RQWM01000005.1         TGTATTTTAAGTGGCAAAGTAACGATTGGAAGTTACTCGCATATAGCAGCGTATACAGCGTTATATGGAGGGGAAGTTGGGATTGAAATGTATGAC
LGCD01000038.1         TGTATTTTAAGTGGCAAAGTAACGATTGGAAGTTACTCGCATATAGCAGCGTATACAGCGTTATATGGAGGGGAAGTTGGGATTGAAATGTATGAC
LGCC01000044.1         TGTATTTTAAGTGGCAAAGTAACGATTGGAAGTTACTCGCATATAGCAGCGTATACAGCGTTATATGGAGGGGAAGTTGGGATTGAAATGTATGAC
Consensus              TGTATTTTAAGTGGCAAAGTAACGATTGGAAGTTACTCGCATATAGCAGCGTATACAGCGTTATATGGAGGGGAAGTTGGGATTGAAATGTATGAC
Conservation
```

```
                       4,900                    4,920                    4,940                    4,960                    4,980
NRIV01000001.1(Sterne) TTTGCAAATATTTCATCGAGAACAATTGTATATGCAGCCATTGATGATTTTAGTGGAAATGCATTAATGGGGCCTACTATTCCAAATCAATATAAA
AAEO01000022.1         TTTGCAAATATTTCATCGAGAACAATTGTATATGCAGCCATTGATGATTTTAGTGGAAATGCATTAATGGGGCCTACTATTCCAAATCAATATAAA
ALNY01000049.1         TTTGCAAATATTTCATCGAGAACAATTGTATATGCAGCCATTGATGATTTTAGTGGAAATGCATTAATGGGGCCTACTATTCCAAATCAATATAAA
AGQP01000002.1         TTTGCAAATATTTCATCGAGAACAATTGTATATGCAGCCATTGATGATTTTAGTGGAAATGCATTAATGGGGCCTACTATTCCAAATCAATATAAA
JSZS01000049.1         TTTGCAAATATTTCATCGAGAACAATTGTATATGCAGCCATTGATGATTTTAGTGGAAATGCATTAATGGGGCCTACTATTCCAAATCAATATAAA
JTAG01000063.1         TTTGCAAATATTTCATCGAGAACAATTGTATATGCAGCCATTGATGATTTTAGTGGAAATGCATTAATGGGGCCTACTATTCCAAATCAATATAAA
ABLH01000027.1         TTTGCAAATATTTCATCGAGAACAATTGTATATGCAGCCATTGATGATTTTAGTGGAAATGCATTAATGGGGCCTACTATTCCAAATCAATATAAA
AAEN01000017.1         TTTGCAAATATTTCATCGAGAACAATTGTATATGCAGCCATTGATGATTTTAGTGGAAATGCATTAATGGGGCCTACTATTCCAAATCAATATAAA
NZ_ABKG01000001.1      TTTGCAAATATTTCATCGAGAACAATTGTATATGCAGCCATTGATGATTTTAGTGGAAATGCATTAATGGGGCCTACTATTCCAAATCAATATAAA
QAEL01000003.1         TTTGCAAATATTTCATCGAGAACAATTGTATATGCAGCCATTGATGATTTTAGTGGAAATGCATTAATGGGGCCTACTATTCCAAATCAATATAAA
NZ_AAEQ01000046.1      TTTGCAAATATTTCATCGAGAACAATTGTATATGCAGCCATTGATGATTTTAGTGGAAATGCATTAATGGGGCCTACTATTCCAAATCAATATAAA
AMDT01000002.1         TTTGCAAATATTTCATCGAGAACAATTGTATATGCAGCCATTGATGATTTTAGTGGAAATGCATTAATGGGGCCTACTATTCCAAATCAATATAAA
JMPU01000016.1         TTTGCAAATATTTCATCGAGAACAATTGTATATGCAGCCATTGATGATTTTAGTGGAAATGCATTAATGGGGCCTACTATTCCAAATCAATATAAA
QAEM01000001.1         TTTGCAAATATTTCATCGAGAACAATTGTATATGCAGCCATTGATGATTTTAGTGGAAATGCATTAATGGGGCCTACTATTCCAAATCAATATAAA
QAEN01000002.1         TTTGCAAATATTTCATCGAGAACAATTGTATATGCAGCCATTGATGATTTTAGTGGAAATGCATTAATGGGGCCTACTATTCCAAATCAATATAAA
JHDS02000015.1         TTTGCAAATATTTCATCGAGAACAATTGTATATGCAGCCATTGATGATTTTAGTGGAAATGCATTAATGGGGCCTACTATTCCAAATCAATATAAA
PVLH01000002.1         TTTGCAAATATTTCATCGAGAACAATTGTATATGCAGCCATTGATGATTTTAGTGGAAATGCATTAATGGGGCCTACTATTCCAAATCAATATAAA
MVOA01000067.1         TTTGCAAATATTTCATCGAGAACAATTGTATATGCAGCCATTGATGATTTTAGTGGAAATGCATTAATGGGGCCTACTATTCCAAATCAATATAAA
QAEH01000002.1         TTTGCAAATATTTCATCGAGAACAATTGTATATGCAGCCATTGATGATTTTAGTGGAAATGCATTAATGGGGCCTACTATTCCAAATCAATATAAA
RQWM01000005.1         TTTGCAAATATTTCATCGAGAACAATTGTATATGCAGCCATTGATGATTTTAGTGGAAATGCATTAATGGGGCCTACTATTCCAAATCAATATAAA
LGCD01000038.1         TTTGCAAATATTTCATCGAGAACAATTGTATATGCAGCCATTGATGATTTTAGTGGAAATGCATTAATGGGGCCTACTATTCCAAATCAATATAAA
LGCC01000044.1         TTTGCAAATATTTCATCGAGAACAATTGTATATGCAGCCATTGATGATTTTAGTGGAAATGCATTAATGGGGCCTACTATTCCAAATCAATATAAA
Consensus              TTTGCAAATATTTCATCGAGAACAATTGTATATGCAGCCATTGATGATTTTAGTGGAAATGCATTAATGGGGCCTACTATTCCAAATCAATATAAA
Conservation

                       5,000                    5,020                    5,040                    5,060                    5,080
NRIV01000001.1(Sterne) AATGTGAAAACTGGAAAAGTTATTTTGAAAAAGCATGTGATTATTGGTGCGCATTCTATTATTTTTCCAAATGTAGTGATAGGAGAAGGAGTGGCA
AAEO01000022.1         AATGTGAAAACTGGAAAAGTTATTTTGAAAAAGCATGTGATTATTGGTGCGCATTCTATTATTTTTCCAAATGTAGTGATAGGAGAAGGAGTGGCA
ALNY01000049.1         AATGTGAAAACTGGAAAAGTTATTTTGAAAAAGCATGTGATTATTGGTGCGCATTCTATTATTTTTCCAAATGTAGTGATAGGAGAAGGAGTGGCA
AGQP01000002.1         AATGTGAAAACTGGAAAAGTTATTTTGAAAAAGCATGTGATTATTGGTGCGCATTCTATTATTTTTCCAAATGTAGTGATAGGAGAAGGAGTGGCA
JSZS01000049.1         AATGTGAAAACTGGAAAAGTTATTTTGAAAAAGCATGTGATTATTGGTGCGCATTCTATTATTTTTCCAAATGTAGTGATAGGAGAAGGAGTGGCA
JTAG01000063.1         AATGTGAAAACTGGAAAAGTTATTTTGAAAAAGCATGTGATTATTGGTGCGCATTCTATTATTTTTCCAAATGTAGTGATAGGAGAAGGAGTGGCA
ABLH01000027.1         AATGTGAAAACTGGAAAAGTTATTTTGAAAAAGCATGTGATTATTGGTGCGCATTCTATTATTTTTCCAAATGTAGTGATAGGAGAAGGAGTGGCA
AAEN01000017.1         AATGTGAAAACTGGAAAAGTTATTTTGAAAAAGCATGTGATTATTGGTGCGCATTCTATTATTTTTCCAAATGTAGTGATAGGAGAAGGAGTGGCA
NZ_ABKG01000001.1      AATGTGAAAACTGGAAAAGTTATTTTGAAAAAGCATGTGATTATTGGTGCGCATTCTATTATTTTTCCAAATGTAGTGATAGGAGAAGGAGTGGCA
QAEL01000003.1         AATGTGAAAACTGGAAAAGTTATTTTGAAAAAGCATGTGATTATTGGTGCGCATTCTATTATTTTTCCAAATGTAGTGATAGGAGAAGGAGTGGCA
NZ_AAEQ01000046.1      AATGTGAAAACTGGAAAAGTTATTTTGAAAAAGCATGTGATTATTGGTGCGCATTCTATTATTTTTCCAAATGTAGTGATAGGAGAAGGAGTGGCA
AMDT01000002.1         AATGTGAAAACTGGAAAAGTTATTTTGAAAAAGCATGTGATTATTGGTGCGCATTCTATTATTTTTCCAAATGTAGTGATAGGAGAAGGAGTGGCA
JMPU01000016.1         AATGTGAAAACTGGAAAAGTTATTTTGAAAAAGCATGTGATTATTGGTGCGCATTCTATTATTTTTCCAAATGTAGTGATAGGAGAAGGAGTGGCA
QAEM01000001.1         AATGTGAAAACTGGAAAAGTTATTTTGAAAAAGCATGTGATTATTGGTGCGCATTCTATTATTTTTCCAAATGTAGTGATAGGAGAAGGAGTGGCA
QAEN01000002.1         AATGTGAAAACTGGAAAAGTTATTTTGAAAAAGCATGTGATTATTGGTGCGCATTCTATTATTTTTCCAAATGTAGTGATAGGAGAAGGAGTGGCA
JHDS02000015.1         AATGTGAAAACTGGAAAAGTTATTTTGAAAAAGCATGTGATTATTGGTGCGCATTCTATTATTTTTCCAAATGTAGTGATAGGAGAAGGAGTGGCA
PVLH01000002.1         AATGTGAAAACTGGAAAAGTTATTTTGAAAAAGCATGTGATTATTGGTGCGCATTCTATTATTTTTCCAAATGTAGTGATAGGAGAAGGAGTGGCA
MVOA01000067.1         AATGTGAAAACTGGAAAAGTTATTTTGAAAAAGCATGTGATTATTGGTGCGCATTCTATTATTTTTCCAAATGTAGTGATAGGAGAAGGAGTGGCA
QAEH01000002.1         AATGTGAAAACTGGAAAAGTTATTTTGAAAAAGCATGTGATTATTGGTGCGCATTCTATTATTTTTCCAAATGTAGTGATAGGAGAAGGAGTGGCA
RQWM01000005.1         AATGTGAAAACTGGAAAAGTTATTTTGAAAAAGCATGTGATTATTGGTGCGCATTCTATTATTTTTCCAAATGTAGTGATAGGAGAAGGAGTGGCA
LGCD01000038.1         AATGTGAAAACTGGAAAAGTTATTTTGAAAAAGCATGTGATTATTGGTGCGCATTCTATTATTTTTCCAAATGTAGTGATAGGAGAAGGAGTGGCA
LGCC01000044.1         AATGTGAAAACTGGAAAAGTTATTTTGAAAAAGCATGTGATTATTGGTGCGCATTCTATTATTTTTCCAAATGTAGTGATAGGAGAAGGAGTGGCA
Consensus              AATGTGAAAACTGGAAAAGTTATTTTGAAAAAGCATGTGATTATTGGTGCGCATTCTATTATTTTTCCAAATGTAGTGATAGGAGAAGGAGTGGCA
Conservation

                       5,100                    5,120                    5,140                    5,160                    5,180
NRIV01000001.1(Sterne) GTTGGTGCAATGAGCATGGTAAAAGAAAGTTTAGATGATTGGTATATTTATGTTGGGGTACCTGTAAGAAAAATAAAAGCCCGTAAAAGAAAGATA
AAEO01000022.1         GTTGGTGCAATGAGCATGGTAAAAGAAAGTTTAGATGATTGGTATATTTATGTTGGGGTACCTGTAAGAAAAATAAAAGCCCGTAAAAGAAAGATA
ALNY01000049.1         GTTGGTGCAATGAGCATGGTAAAAGAAAGTTTAGATGATTGGTATATTTATGTTGGGGTACCTGTAAGAAAAATAAAAGCCCGTAAAAGAAAGATA
AGQP01000002.1         GTTGGTGCAATGAGCATGGTAAAAGAAAGTTTAGATGATTGGTATATTTATGTTGGGGTACCTGTAAGAAAAATAAAAGCCCGTAAAAGAAAGATA
JSZS01000049.1         GTTGGTGCAATGAGCATGGTAAAAGAAAGTTTAGATGATTGGTATATTTATGTTGGGGTACCTGTAAGAAAAATAAAAGCCCGTAAAAGAAAGATA
JTAG01000063.1         GTTGGTGCAATGAGCATGGTAAAAGAAAGTTTAGATGATTGGTATATTTATGTTGGGGTACCTGTAAGAAAAATAAAAGCCCGTAAAAGAAAGATA
ABLH01000027.1         GTTGGTGCAATGAGCATGGTAAAAGAAAGTTTAGATGATTGGTATATTTATGTTGGGGTACCTGTAAGAAAAATAAAAGCCCGTAAAAGAAAGATA
AAEN01000017.1         GTTGGTGCAATGAGCATGGTAAAAGAAAGTTTAGATGATTGGTATATTTATGTTGGGGTACCTGTAAGAAAAATAAAAGCCCGTAAAAGAAAGATA
NZ_ABKG01000001.1      GTTGGTGCAATGAGCATGGTAAAAGAAAGTTTAGATGATTGGTATATTTATGTTGGGGTACCTGTAAGAAAAATAAAAGCCCGTAAAAGAAAGATA
QAEL01000003.1         GTTGGTGCAATGAGCATGGTAAAAGAAAGTTTAGATGATTGGTATATTTATGTTGGGGTACCTGTAAGAAAAATAAAAGCCCGTAAAAGAAAGATA
NZ_AAEQ01000046.1      GTTGGTGCAATGAGCATGGTAAAAGAAAGTTTAGATGATTGGTATATTTATGTTGGGGTACCTGTAAGAAAAATAAAAGCCCGTAAAAGAAAGATA
AMDT01000002.1         GTTGGTGCAATGAGCATGGTAAAAGAAAGTTTAGATGATTGGTATATTTATGTTGGGGTACCTGTAAGAAAAATAAAAGCCCGTAAAAGAAAGATA
JMPU01000016.1         GTTGGTGCAATGAGCATGGTAAAAGAAAGTTTAGATGATTGGTATATTTATGTTGGGGTACCTGTAAGAAAAATAAAAGCCCGTAAAAGAAAGATA
QAEM01000001.1         GTTGGTGCAATGAGCATGGTAAAAGAAAGTTTAGATGATTGGTATATTTATGTTGGGGTACCTGTAAGAAAAATAAAAGCCCGTAAAAGAAAGATA
QAEN01000002.1         GTTGGTGCAATGAGCATGGTAAAAGAAAGTTTAGATGATTGGTATATTTATGTTGGGGTACCTGTAAGAAAAATAAAAGCCCGTAAAAGAAAGATA
JHDS02000015.1         GTTGGTGCAATGAGCATGGTAAAAGAAAGTTTAGATGATTGGTATATTTATGTTGGGGTACCTGTAAGAAAAATAAAAGCCCGTAAAAGAAAGATA
PVLH01000002.1         GTTGGTGCAATGAGCATGGTAAAAGAAAGTTTAGATGATTGGTATATTTATGTTGGGGTACCTGTAAGAAAAATAAAAGCCCGTAAAAGAAAGATA
MVOA01000067.1         GTTGGTGCAATGAGCATGGTAAAAGAAAGTTTAGATGATTGGTATATTTATGTTGGGGTACCTGTAAGAAAAATAAAAGCCCGTAAAAGAAAGATA
QAEH01000002.1         GTTGGTGCAATGAGCATGGTAAAAGAAAGTTTAGATGATTGGTATATTTATGTTGGGGTACCTGTAAGAAAAATAAAAGCCCGTAAAAGAAAGATA
RQWM01000005.1         GTTGGTGCAATGAGCATGGTAAAAGAAAGTTTAGATGATTGGTATATTTATGTTGGGGTACCTGTAAGAAAAATAAAAGCCCGTAAAAGAAAGATA
LGCD01000038.1         GTTGGTGCAATGAGCATGGTAAAAGAAAGTTTAGATGATTGGTATATTTATGTTGGGGTACCTGTAAGAAAAATAAAAGCCCGTAAAAGAAAGATA
LGCC01000044.1         GTTGGTGCAATGAGCATGGTAAAAGAAAGTTTAGATGATTGGTATATTTATGTTGGGGTACCTGTAAGAAAAATAAAAGCCCGTAAAAGAAAGATA
Consensus              GTTGGTGCAATGAGCATGGTAAAAGAAAGTTTAGATGATTGGTATATTTATGTTGGGGTACCTGTAAGAAAAATAAAAGCCCGTAAAAGAAAGATA
Conservation
```

	5,200 / 5,220	
NRIV01000001.1(Sterne)	GTAGAATTAGAAAATGAATTTTTAAAAAGTATGAACTCTTGA	5210
AAEO01000022.1	GTAGAATTAGAAAATGAATTTTTAAAAAGTATGAACTCTTGA	5210
ALNY01000049.1	GTAGAATTAGAAAATGAATTTTTAAAAAGTATGAACTCTTGA	5210
AGQP01000002.1	GTAGAATTAGAAAATGAATTTTTAAAAAGTATGAACTCTTGA	5210
JSZS01000049.1	GTAGAATTAGAAAATGAATTTTTAAAAAGTATGAACTCTTGA	5210
JTAG01000063.1	GTAGAATTAGAAAATGAATTTTTAAAAAGTATGAACTCTTGA	5210
ABLH01000027.1	GTAGAATTAGAAAATGAATTTTTAAAAAGTATGAACTCTTGA	5210
AAEN01000017.1	GTAGAATTAGAAAATGAATTTTTAAAAAGTATGAACTCTTGA	5210
NZ_ABKG01000001.1	GTAGAATTAGAAAATGAATTTTTAAAAAGTATGAACTCTTGA	5210
QAEL01000003.1	GTAGAATTAGAAAATGAATTTTTAAAAAGTATGAACTCTTGA	5210
NZ_AAEQ01000046.1	GTAGAATTAGAAAATGAATTTTTAAAAAGTATGAACTCTTGA	5210
AMDT01000002.1	GTAGAATTAGAAAATGAATTTTTAAAAAGTATGAACTCTTGA	5210
JMPU01000016.1	GTAGAATTAGAAAATGAATTTTTAAAAAGTATGAACTCTTGA	5210
QAEM01000001.1	GTAGAATTAGAAAATGAATTTTTAAAAAGTATGAACTCTTGA	5210
QAEN01000002.1	GTAGAATTAGAAAATGAATTTTTAAAAAGTATGAACTCTTGA	5210
JHDS02000015.1	GTAGAATTAGAAAATGAATTTTTAAAAAGTATGAACTCTTGA	5210
PVLH01000002.1	GTAGAATTAGAAAATGAATTTTTAAAAAGTATGAACTCTTGA	5209
MVOA01000067.1	GTAGAATTAGAAAATGAATTTTTAAAAAGTATGAACTCTTGA	5210
QAEH01000002.1	GTAGAATTAGAAAATGAATTTTTAAAAAGTATGAACTCTTGA	5210
RQWM01000005.1	GTAGAATTAGAAAATGAATTTTTAAAAAGTATGAACTCTTGA	5211
LGCD01000038.1	GTAGAATTAGAAAATGAATTTTTAAAAAGTATGAACTCTTGA	5222
LGCC01000044.1	GTAGAATTAGAAAATGAATTTTTAAAAAGTATGAACTCTTGA	5217
Consensus	GTAGAATTAGAAAATGAATTTTTAAAAAGTATGAACTCTTGA	

Conservation

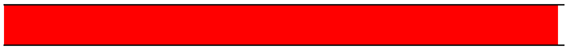

Supplement: Supplementary file 2 — Additional file 2: Figure S1. Alignment of all B. anthracis anthrose operons showing 97–100% to Sterne. The anthrose operon of all sequenced B. anthracis strains were aligned to sequences from Sterne using CLC viewer. [file 12866_2019_1693_MOESM2_ESM.pdf]
