# Supplementary material for: Nucleotide polymorphism assay for the identification of west African group Bacillus anthracis: a lineage lacking anthrose
Source: BMC Microbiol. 2020 Jan 7;20:6. doi: 10.1186/s12866-019-1693-2 (PMC6947953; doi:10.1186/s12866-019-1693-2)
Supplement: Supplementary file 3 — Additional file 3: Table S2. Cycle threshold values with 1 and 5 ng of DNA. Table S3. Cycle threshold values for dilution curve with Sterne DNA. [file 12866_2019_1693_MOESM3_ESM.pdf]

Table S2. Cycle threshold values with 1 and 5 ng of DNA

Assay	Strain	DNA Conc.	Mean C_T	Range	SD
892	Sterne	1 ng	20.3	20.0-20.7	0.31
892	UF 01063	1 ng	19.3	19.1-19.6	0.21
892	Sterne	5 ng	18.0	17.4-18.5	0.49
892	UF 01063	5 ng	16.9	16.8-16.9	0.06
1352	Sterne	1 ng	21.2	21.1-21.4	0.14
1352	UF 01063	1 ng	20.5	19.9-20.9	0.44
1352	Sterne	5 ng	19.0	18.8-19.2	0.19
1352	UF 01063	5 ng	17.8	17.0-18.1	0.51

*SD- Standard deviation

Table S3. Cycle threshold values for dilution curve with Sterne DNA

DNA Conc.	892 Mean C_T	892 SD*	1352 Mean C_T	1352 SD*
100 pg	22.6	0.06	23.5	0.05
10 pg	26.2	0.01	27.4	0.20
1 pg	29.6	0.07	31	0.04
100 fg	33.8	0.16	34.6	0.32
10 fg	37.1	0.13	39.1	0.14

*SD- Standard deviation
