# Supplementary figures and images for: Nucleotide polymorphism assay for the identification of west African group Bacillus anthracis: a lineage lacking anthrose
Source: BMC Microbiol. 2020 Jan 7;20:6. doi: 10.1186/s12866-019-1693-2 (PMC6947953; doi:10.1186/s12866-019-1693-2)

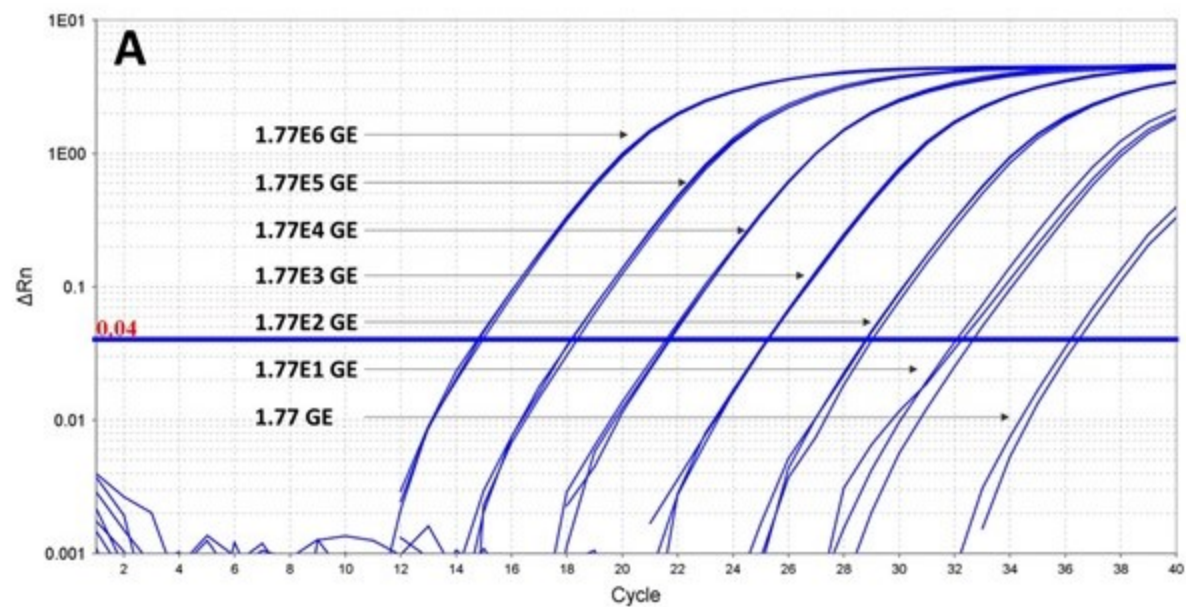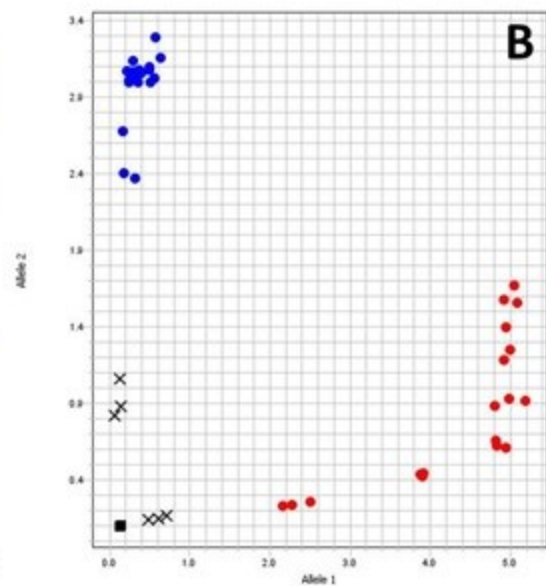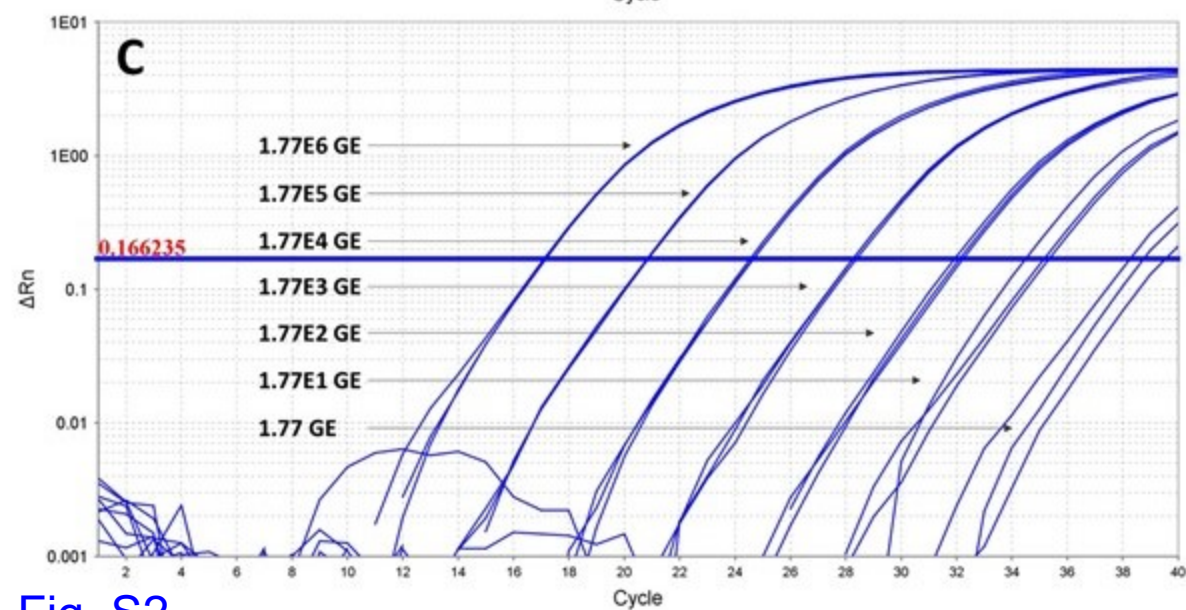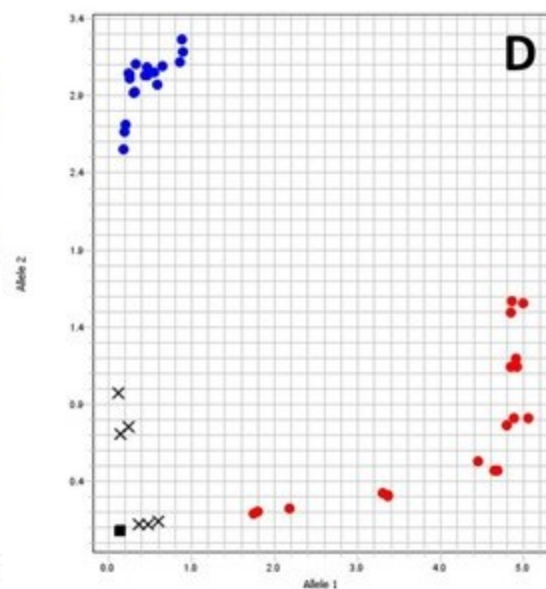

Fig. S2

Supplement: Supplementary file 4 — Additional file 4: Figure S2. Real-time amplification plots of the 892 (A) and 1352 (C) rhAmp genotyping assays showing dilution curves of anthrose control plasmids. Ten-fold serial dilutions ranging from 1.77E6 to 1.77 GE of both anthrose positive and negative plasmids were tested in triplicate (only fluorescence from Sterne specific allele primer is shown). The average CT values for C892T assay were as follows: 1.77E6 GE, 15.6; 1.77E5 GE, 18.9; 1.77E4 GE, 22.4; 1.77E3 GE, 25.9; 177 GE, 29.5; 17.7 GE, 33.3; and 1.77, 36.8. The standard curve displayed a slope of − 3.5578 and R2 of 0.9997. For the 1352 assay the average CT values were: 1.77E6 GE, 17.1; 1.77E5 GE, 20.8; 1.77E4 GE, 24.6; 1.77E3 GE, 28.3; 177 GE, 31.9; 17.7 GE, 35.0; and 1.77 GE, 38.3. The standard curve had a slope of − 3.5433 and R2 of 0.9988. Amplification at the 1.77-GE level was not consistent in either of the assays. The corresponding endpoint allelic discrimination plots for the 892 (B) and 1352 (D) assays depict the Sterne antABCD operon-carrying plasmid as red clusters and the WAG anthrose operon-carrying plasmid in blue. Undetermined calls corresponding to 1.77 GE of plasmid DNA are depicted as black Xs. Black squares near the plot origin represent negative controls. [file 12866_2019_1693_MOESM4_ESM.pdf]

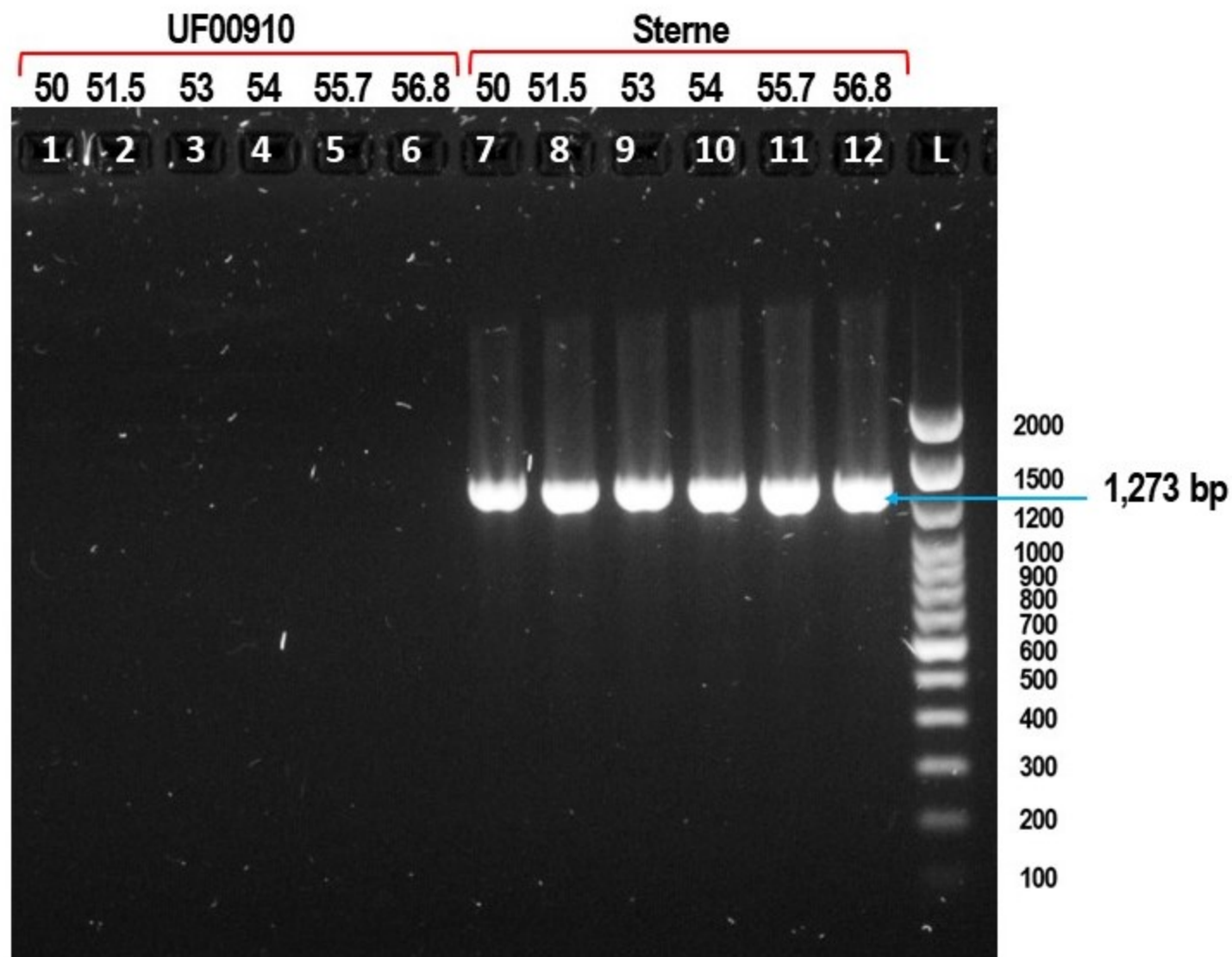

Fig. S3

Supplement: Supplementary file 5 — Additional file 5: Figure S3. Gradient PCR for amplification of the antC gene of the anthrose operon. A 1.2-kb amplicon was observed with Sterne DNA (lanes 7–12) after amplification with primers specific for the antC of the Sterne anthrose operon. No products were detected with DNA from UF00910 (lanes 1–6). [file 12866_2019_1693_MOESM5_ESM.pdf]

Fig. S4

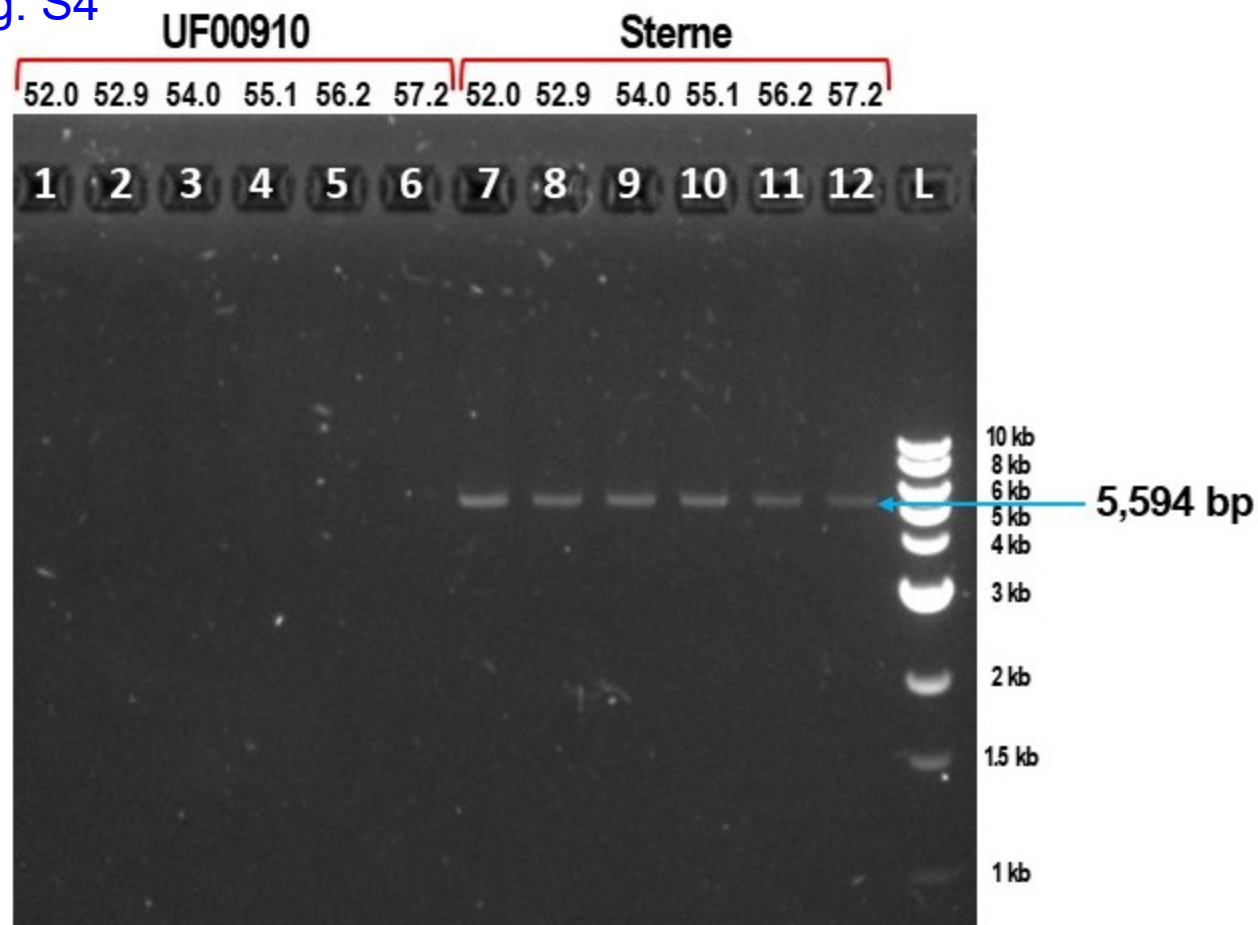

Supplement: Supplementary file 6 — Additional file 6: Figure S4. Gradient PCR for amplification of the antABCD operon. A 5.6-kb amplicon was observed with Sterne DNA (lanes 7–12) after amplification with primers specific for the anthrose operon of Sterne. No products were detected with DNA from UF00910 (lanes 1–6). [file 12866_2019_1693_MOESM6_ESM.pdf]
